# DNA Methylation Changes and Its Associated Genes in Mulberry (*Morus alba* L.) Yu-711 Response to Drought Stress Using MethylRAD Sequencing

**DOI:** 10.3390/plants11020190

**Published:** 2022-01-12

**Authors:** Michael Ackah, Liangliang Guo, Shaocong Li, Xin Jin, Charles Asakiya, Evans Tawiah Aboagye, Feng Yuan, Mengmeng Wu, Lionnelle Gyllye Essoh, Daniel Adjibolosoo, Thomas Attaribo, Qiaonan Zhang, Changyu Qiu, Qiang Lin, Weiguo Zhao

**Affiliations:** 1 Jiangsu Key Laboratory of Sericultural Biology and Biotechnology, School of Biotechnology, Jiangsu University of Science and Technology, Zhenjiang 212100, China; 192310021@stu.just.edu.cn (L.G.); ShaocongLi1@126.com (S.L.); jinxin9502@126.com (X.J.); fengyuan6181@126.com (F.Y.); 199310015@stu.just.edu.cn (M.W.); lionnelle.92@gmail.com (L.G.E.); Loer9725@126.com (Q.Z.); 2 Key Laboratory of Precision Nutrition and Food Quality, Department of Nutrition and Health, China Agricultural University, Beijing 100083, China; asakiya@cau.edu.cn; 3 Key Laboratory of Plant Pathology, College of Plant Protection, China Agricultural University, Beijing 100193, China; vanx@cau.edu.cn; 4 Key Laboratory of Cotton Genetics, Genomics and Breeding, College of Agronomy and Biotechnology, China Agricultural University, Beijing 100193, China; selaseiyaah@gmail.com; 5 School of Agriculture, C. K. Tedam University of Technology and Applied Sciences, Navrongo UK-0215-5321, Ghana; tattaribo@cktutas.edu.gh; 6 Sericultural Research Institute, Guangxi Zhuang Autonomous Region, Nanning 530007, China; Changyuqiu2008@163.com (C.Q.); gxlq67@163.com (Q.L.)

**Keywords:** MethylRAD, DNA methylation, mulberry, drought stress, gene regulation

## Abstract

Drought stress remains one of the most detrimental environmental cues affecting plant growth and survival. In this work, the DNA methylome changes in mulberry leaves under drought stress (EG) and control (CK) and their impact on gene regulation were investigated by MethylRAD sequencing. The results show 138,464 (37.37%) and 56,241 (28.81%) methylation at the CG and CWG sites (W = A or T), respectively, in the mulberry genome between drought stress and control. The distribution of the methylome was prevalent in the intergenic, exonic, intronic and downstream regions of the mulberry plant genome. In addition, we discovered 170 DMGs (129 in CG sites and 41 in CWG sites) and 581 DMS (413 in CG sites and 168 in CWG sites). Kyoto Encyclopedia of Genes and Genomes (KEGG) enrichment analysis indicates that phenylpropanoid biosynthesis, spliceosome, amino acid biosynthesis, carbon metabolism, RNA transport, plant hormone, signal transduction pathways, and quorum sensing play a crucial role in mulberry response to drought stress. Furthermore, the qRT-PCR analysis indicates that the selected 23 genes enriched in the KEGG pathways are differentially expressed, and 86.96% of the genes share downregulated methylation and 13.04% share upregulation methylation status, indicating the complex link between DNA methylation and gene regulation. This study serves as fundamentals in discovering the epigenomic status and the pathways that will significantly enhance mulberry breeding for adaptation to a wide range of environments.

## 1. Introduction

Plants are sessile organisms and are continually exposed to biotic and abiotic challenges, including heat, water deficit, and pathogens. For plants to adapt to these situations requires frequent and constant modifications at molecular and morphological levels. Epigenetic regulations provide these efficient and effective controls, which promote plant survival by increasing stress tolerance [1]. Epigenetics is the study of heritable changes in gene expression that do not occur due to changes in DNA sequence [2]. It is a defense mechanism used by plants to significantly facilitate genomic plasticity and functions in plant growth, development, and adaptation to stresses [2]. Therefore, epigenetic profiles connected to certain phenotypes and environmental cues become critical to comprehend their contribution to crop improvement [1]. Epigenetic regulatory mechanisms, including DNA methylation, histone modification, and RNA interference (RNAi), play significant roles in gene regulation and genome stability [1]. DNA methylation is one of the most frequent epigenetic changes found in all eukaryotic genomes. It is a chemical alteration mediated mainly by cytosine methyltransferase that involves a methyl group added to position 5 of the pyrimidine ring in the cytosine residue in the DNA sequence [1,3]. Its role is vital in many biological processes such as embryogenesis, cellular differentiation, X-chromosome inactivation, genomic imprinting, and transposon silencing [4].

DNA methylation patterns occur diversely in eukaryotic organisms. Its pattern is sequence-specific which occurs at CG, CHG, and CHH (H = A, C, or T) nucleotides of gene and transposable elements (TEs) [2]. In plants, all the three-sequences type occurs, but in mammals, the CpG sequence is mostly where DNA methylation occurs [5]. Different mechanisms involve these sequence contexts in establishing, maintaining, and removing methyl groups. In the Arabidopsis genome, DNA methylation levels at distinct positions have been reported to constitute 24%, 6.7%, 1.7% in the sequence contexts of CG, CHG, and CHH, respectively [1]. DNA methylation is classified into symmetrical or asymmetrical based on the target gene sequence. The CG and CHG contexts are symmetrical, while CHH conforms to the asymmetrical context [1].

Several reports on the impact of DNA methylation on plant growth and development, defense, biotic and abiotic responses, including exposure to drought, salt, and heavy metals, have been documented [6,7]. According to Fraga and colleagues, plant growth regulator (PGR) treatment increased global DNA methylation in *Araucaria angustifolia* during long-term subcultures and led to compromising genomic stability and changing gene expression [8]. The activation of the OsMYB91 gene in rice was associated with rapid demethylation of the gene’s promoter region and histone modification of the locus, indicating that dynamic methylation patterns may play a role in gene regulation [9].

Drought stress is a major environmental disaster affecting plant growth and development [10]. With the current global climate change, it is predicted that about 20% of the world’s land surface is expected to be in drought at any given time [11]. This condition will eventually result in agricultural production losses and will negatively affect the ecological environment. Drought stress induces a complex series of physiological, biochemical, and genetic reactions in plants. The methylation of DNA, gene expression, and metabolic networks play a role in these intricate processes [3]. According to studies, drought stress alters DNA methylation patterns in tissue-specific, variety-specific, and stress-specific mechanisms [12]. In the *Populus trichocarpa*, the magnitude to which genomic changes in DNA methylation occurred was related to the amplitude of drought-induced transcriptional changes, emphasizing the importance of epigenetic mechanisms in tress adaptation to environmental conditions and long-term survival [13]. In studying the DNA methylation levels in the sesame plant, it was revealed that drought stress strongly causes a lot of de novo methylation (DNM) in the *Sesamum indicum* genome; however, upon recovery phase, most of the methylated loci were demethylated (DM) [14].

A recent report discovered that drought stress strongly induced about 8.64% DNA methylation in the mulberry plant under drought stress than those irrigated daily [2]. Furthermore, in two wheat genotypes during drought stress, demethylation occurred more frequently in the drought-tolerant genotype (C306) than in the drought-sensitive genotype (HUW468), when methylation patterns were analyzed [15]. Thus, these studies establish a direct relationship between drought stress and DNA methylation patterns.

Given that changes in DNA methylation occur in a small fraction of the genome and have great regulatory potential, there is a need to develop high-throughput techniques for assessing changes in DNA methylation at these sites in a specific and accurate manner [16]. While profiling the complete methylome at single-base resolution with whole-genome bisulfite sequencing (WGBS) is ideal, it is extremely expensive for large numbers of samples [4]. Rather than that, the most generally used approaches address this issue by employing various ways to lower the cost of sequencing. These methods can be categorized into three broad categories based on their methodological principles: (i) bisulfite conversion-based methods (e.g., RRBS), (ii) immunoprecipitation-based methods (e.g., MeDIP-seq) and MethylCap-seq), and (iii) restriction enzyme-based methods (e.g., MethylSeq).

However, none of them provides the ‘ideal solution,’ with each having its own set of strengths and weaknesses [4]. Methylation-dependent restriction enzymes (MethylRAD) serve as a unique tool for determining the methylation status of bases. MethylRAD sequencing employs the methylation-dependent restriction enzyme FspEI, making 10–16 base pair (bps) cuts next to the methylated cytosine [16]. FspEI is a Mrr-like enzyme of type IIS that recognizes 5-methylcytosine (5 mC) and 5-hydroxymethylcytosine (5 hmC) in C^m^C and ^m^CDS sites (D = A or T; S = C or G) [16]. It produces a double-stranded cleavage on the 3’ ends of the modified cytosine at a predetermined distance (N12/N16), cutting bi-directionally to yield 32 base-pair fragments [16].

Mulberry (*Morus alba*) is a significant perennial economic tree native to China with a wide ecological spread. Besides its application in sericulture, the plant is economically and ecologically important [10]. Mulberry is extremely adaptable to unfavorable climatic environments such as drought, cold, excessive salt, waterlogging, and metal ion exposure [17,18]. The mulberry plant genome and transcriptome have been decoded under drought stress [19,20]. Due to the availability of high-quality reference genomes and developments in sequencing methods, whole-genome methylome analyses of plant genomes have become possible. For example, newly identified rice DNA methylation mechanisms have enabled whole-genome methylome analyses [21]. In apples, the cytosine methylation occurrence was associated with drought stress [22]. In a recent report, Li and colleagues found that cytosine methylation was associated with drought stress in mulberry [2]. The authors reveal that the methylation level accounted for 8.64% in the drought stress than in control [2]. In another report, single-base resolution methylomes of upland cotton (*Gossypium hirsutum* L.) reveal epigenome modifications associated with water deficit [23]. However, studies on the methylome of the mulberry plant concerning drought stress are rare, limiting the knowledge and evidence on mulberry methylome changes during drought stress.

In this present study, we generated genome-wide high-coverage DNA methylation maps using MethylRAD sequencing [4] in mulberry plants under drought stress and control treatment to investigate the methylome changes on the whole-genome epigenome reprogramming in mulberry. The study’s objective is to (a) determine mulberry methylome changes associated with water deficit; (b) evaluate whether the methylome changes with drought-stress affect changes in gene expression in mulberry. This study will significantly improve our understanding of the effect of drought stress on methylation levels and its relation to gene expression and provide a platform for investigating and facilitating the breeding of resistance mulberry.

## 2. Results

### 2.1. Physiological Changes in Mulberry (Yu-711) in Response to Drought Stress and Control Treatment

**Figure 1:**
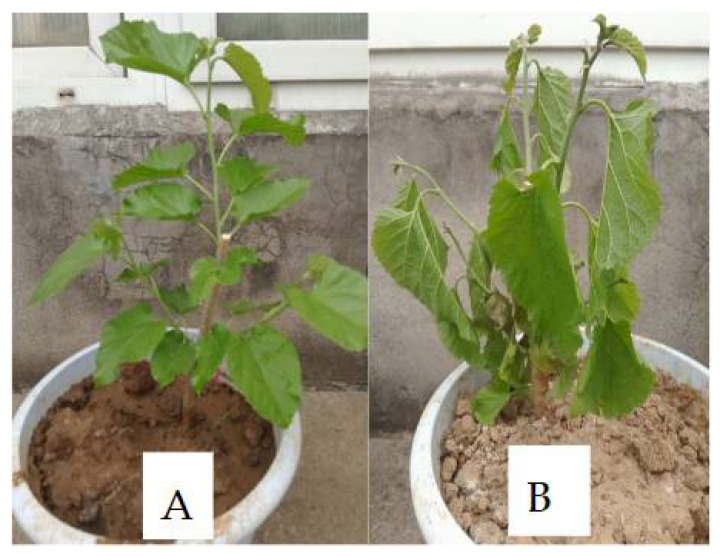
Physiological responses of mulberry leaves affected by drought and the control treatment. (**A**) Mulberry plant under control treatment. (**B**) Mulberry plant under drought stress treatment at the five-day time point.

Mulberry plant leaves exhibited morphological changes during drought stress when the plants were exposed to water deficits, as shown in Figure 1. The physiological parameters, including relative water content (RWC) and leaf lengths of plants under drought stress and control, were examined before DNA methylome examination to show how drought stress advances. More interpretation of the physiological parameter data can be found in our previous study [10].

### 2.2. Analysis of MethylRAD Data from Illumina Sequencing and QC

To examine the global distribution of methylation sites on the mulberry plant under drought stress, four (4) samples, two control samples (CK), and two drought stress samples (EG) MethylRAD libraries were constructed from genomic DNA isolated from mulberry leaves with different treatments (control and drought) and sequenced on the Illumina Hiseq X Ten Nova PE150 platform (OE Biotech Co., Ltd., Shanghai, China). The obtained methylomes were decoded and analyzed. Our data have been deposited at the NCBI Sequence Read Archive (SRA) (https://ncbi.nlm.nih.gov/subs/sra; available online since 30 December 2021) with an accession number PRJNA771759. An overview of the MethylRAD sequencing reads derived from the four libraries is listed in Table 1. In total, a range of 11,649,483 to 12,694,691 clean reads was obtained from the four samples (EG and CK), representing a percentage of 29.29% to 32.32% (Table 1). In addition, the base distribution and the base mass distribution of the clean reads and the proportion of A/C/G/T/N at each location and the sequencing base mass at each location were determined (Figure S1). After filtering and deletion of the tags that did not contain the expected enzyme restriction sites, the clean data with a range of 1,689,487 to 1,992,606 representing 14.28% to 15.70% (Table 2), was uniquely mapped to mulberry notabilis reference genome ASM41409v2, using bowtie2 (version 2.3.4.3) with the-no-unal parameters [24].

### 2.3. DNA Methylation Profiles of the Mulberry Seedlings Leaves

**Figure 2:**
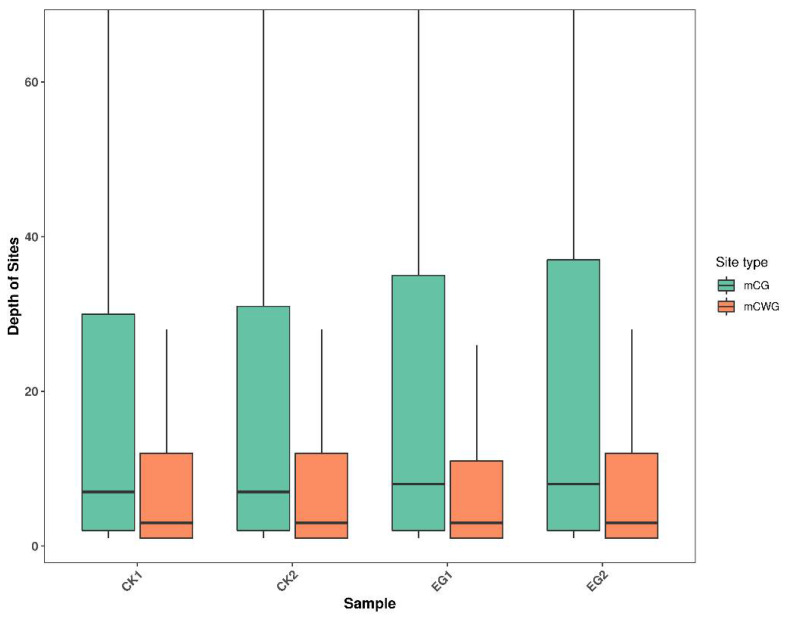
Distribution of MethylRAD sequencing data in mulberry samples. mCG is the methylation at the CG sites; mCWG is the methylation at the CWG sites, where W = A or T.

We employed uniquely mapped reads to locate methylated peaks to analyze the genome-wide DNA methylation profiles of mulberry leaves exposed to drought and control treatments. Under this, the sum of the CG and CWG methylation loci in each genome sample was determined. We obtained 370,598 mCG sites and 195,193 mCWG sites (Table S1). We further determined the reliable methylated sites by using a cut-off read coverage of not less than five reads for each site in each of the four libraries. On average, 34,269 CG and 13,744 CWG DNA methylation sites with an average depth of 33.02 and 26.64, respectively, were found in CK. On average, 34,964 mCG and 14,387 mCWG sites with an average depth of 36.99 and 25.71, respectively, were found in EG (Table 3, Figure 2). Thus, these results indicate that the level of DNA methylation at the CG sites was more significant than at the CWG sites in mulberry leaves under drought and control treatment.

Furthermore, DNA methylation at CG increased by 0.19%, whereas the methylation at the CWG also increased by 0.32% in the drought treatment compared to the control. In addition, the distribution of the methylation site on the chromosome on the whole genome was counted as a sliding window to determine how frequently the methylation site of CG and CWG occurred in the chromosome. We employed circos software (version 0.69.6) set at the default parameters [25] to draw a line chart of the frequency distribution (Figure S2). As a result, CG and CWG levels’ methylation site frequently occurred in the chromosomes NW_010362482.1 and NW_010367622.1. However, the occurrence of the CG sites was more frequent than that of the CWG levels in the genome (Figure S2).

### 2.4. Distribution of DNA Methylation Sites in the Different Functional Components of the Genome during Drought and Control Treatment

**Figure 3:**
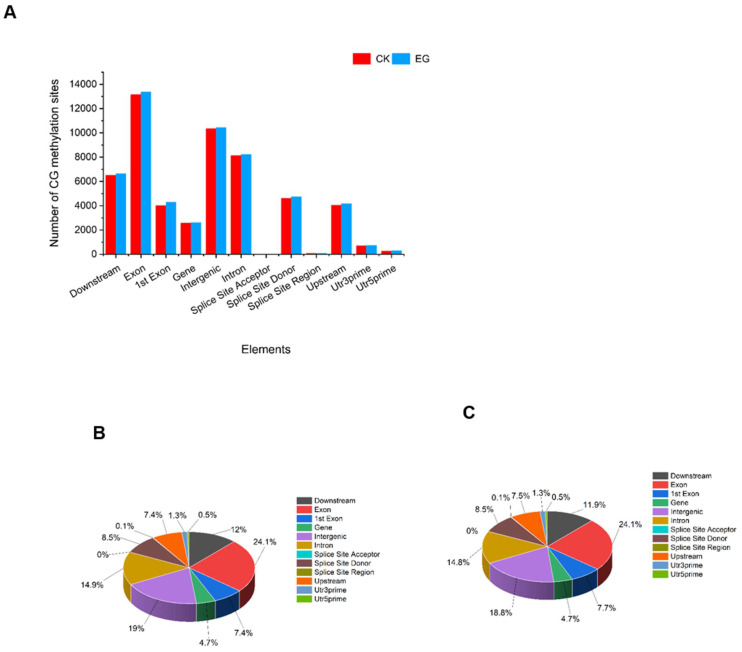
Distribution of methylation sites in different gene functional elements. (**A**) The distribution of mCG in elements count. (**B**) The proportion of the mCG distribution in the CK samples. (**C**) The proportion of the mCG distribution in the EG samples. CK is the control sample, and EG is the drought-stress sample.

We analyzed the distribution of the methylated site at the CG and CWG from the MethylRAD data in the different gene components of the genome using BEDTools software (v2.25.0) set at echo-count -delim parameter [26], after annotating the genome with SnpEff (v4.1g) software [27]. The various genetic components analyzed include utr3prime, utr5prime, exon, 1st exon, intron, intergenic, downstream, gene, splice site acceptor, splice site donor, splice site region, and upstream. At the CG level, the results show that the DNA methylation sites were mainly distributed in the exon, followed by intergenic regions, intron regions, and then downstream regions (Figure 3, Table S2). The regions with minimal CG sites distribution include splice site acceptor followed by splice site region, utr5prime, and utr3prime. Interestingly, there was a dynamic trend in the fractional distribution of the CG methylation sites when CK and EG treatment were compared. Some functional components had a substantial gain or loss of CG methylation site in the EG, while others remained unchanged from the CK (Figure 3B,C).

**Figure 4:**
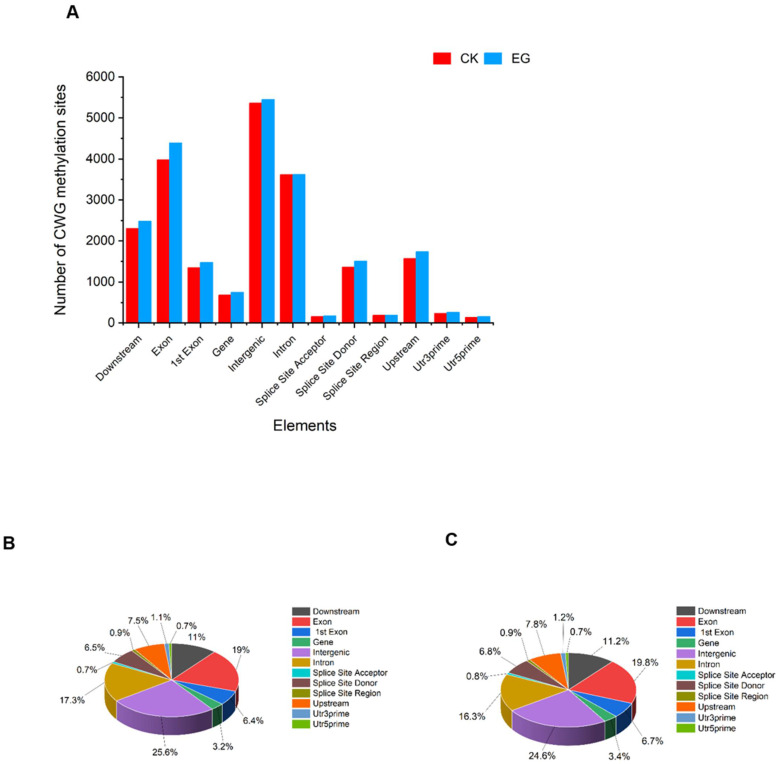
Distribution of methylation sites in different gene functional elements. (**A**) The distribution of mCWG in elements count. (**B**) The proportion of the mCWG (W = A or T) distribution in the CK samples. (**C**) The proportion of the mCWG distribution in the EG samples. CK is the control sample, and EG is the drought-stress sample.

At the CWG level, the DNA methylation site distribution lied mostly within the intergenic region, followed by the exon, intron, and downstream in the EG and CK (Figure 4, Table S2). The fewest CWG site distribution region includes splice site acceptor followed by utr5prime, splice site region, and utr3prime. We also observed a dynamic trend in the fractional distribution of the CWG methylation on the functional component of the genome. For instance, at the intron region, the distribution in the EG decreased (16.3%) compared to the CK (17.3%). However, the distribution at the downstream, exon, 1st exon, upstream, gene, utr3prime, splice site donor increased in the EG treatment compared to the CK. In addition, in the exon region, the distribution was 19% in the CK compared to 19.8% in the EG (Figure 4B,C). Thus, although the methylation sites of CG were more than the CWG sites, the patterns of CG and CWG distribution sites in all the samples (EG and CK) were similar. The CG and CWG methylated sites were concentrated in the genome’s exon, intergenic, intron, and downstream. Altogether, the drought stress caused overlapping changes in the CG and CWG sites distribution in the functional composition of the genome.

### 2.5. Distribution of DNA Methylation Sites in TSS, Gene Body, TTS Region

**Figure 5:**
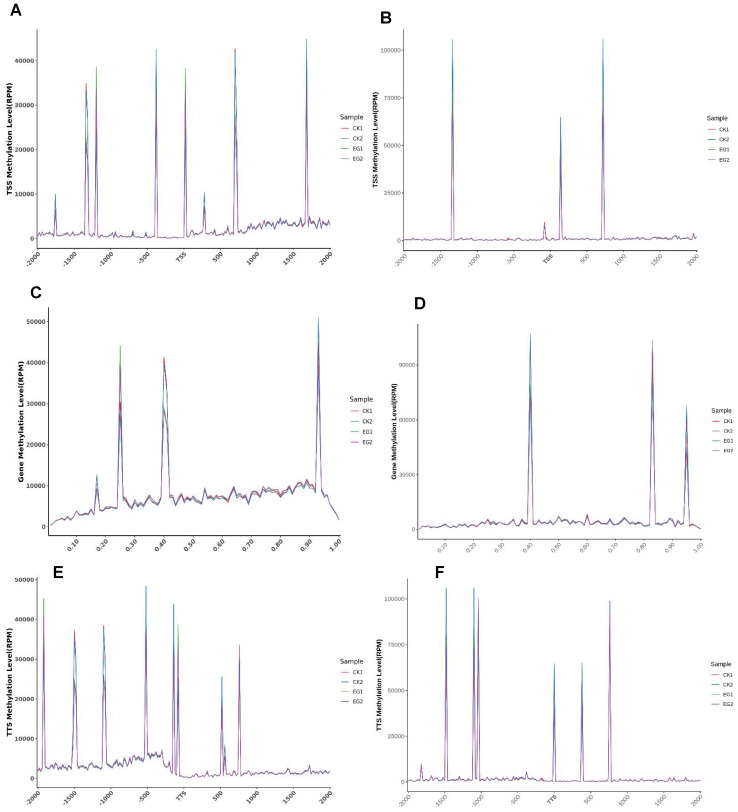
Distribution of methylation sites in transcription start site (TSS), gene body, and transcription termination site (TTS). (**A**,**B**) The distribution of CG and CWG methylation level in the TSS; (**C**,**D**) the distribution of CG and CWG methylation level in the gene body; (**E**,**F**) the distribution of CG and CWG methylation level in the TTS.

We analyzed the distribution of the CG and CWG within the 2-kb segment upstream and downstream of the gene transcription starting position (TSS; Figure 5A,B), the gene body (Figure 5C,D), and transcription termination site (TSS, Figure 5E,F). The results reveal that the DNA methylation levels were higher in the TSS and TTS than in the gene body. However, the distribution of the CG and CWG in EG was relatively higher than in the CK samples.

### 2.6. Analysis of DNA Methylation Level Correlation between Samples

The correlation analysis was performed on the samples to understand better the collinearity of the two data (to measure the consistency of the sample data between the CK and the EG samples (Figure S3).

### 2.7. Analysis of Differential Methylated Site (DMS) and Gene (DMGs)

**Figure 6:**
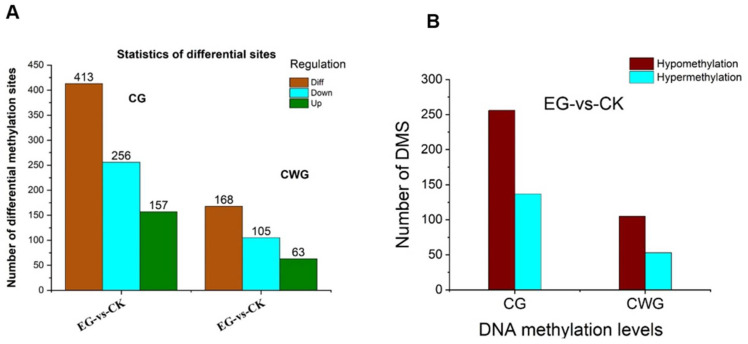

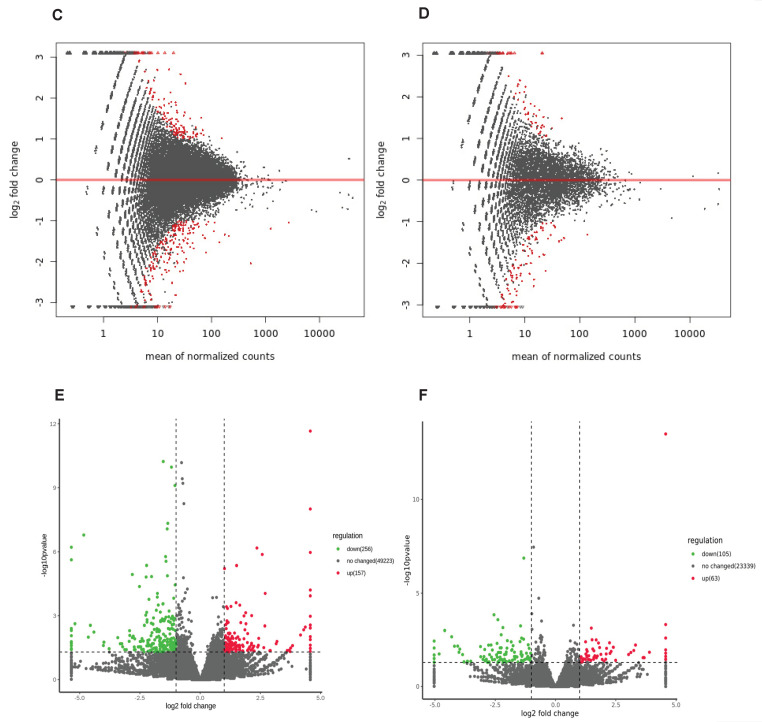
Differential methylation in CG and CWG sites between EG-vs.-CK. (**A**) The number of differentially methylated CG and CWG sites. (**B**) the number of hypo/hypermethylated CG and CWG sites. (**C**,**D**) MA plot of DMS in CG and CWG sites. (**E**,**F**) Volcano plot of the DMS in CG and CWG sites. The small red circle represents upregulated DMS. The blue and dark grey color means the downregulated DMS and non-significant methylated sites.

The sum of all methylation site levels within a gene represents the methylation level of the gene. Using DESeq v1.18.0 software [28], two screening criteria were used to evaluate the differential methylated sites and the differential methylated genes; first, fold-change (FC) greater than 2 or less than 0.5 and a *p* < 0.05. When the FC > 2, the expression is upregulated, and when FC < 0.5, the expression is downregulated. A total of 49,636 CG methylated sites and 23,507 CWG methylated sites were found between the drought-stress and the control plants (Table S3). However, 413 CG and 168 CWG DMS were identified in EG-vs.-CK (Figure 6A, Table S3). The results reveal that 157 mCG sites were up-and 256 downregulated in the EG samples compared to the control. Additionally, 63 mCWG sites were upregulated, and 105 were downregulated. We analyzed the hyper/hypomethylation of the DMS in both the CG and CWG levels in EG and CK. Remarkably, 256 DMS were hypomethylated, and 137 DMS were hypermethylated in the CG sites (Figure 6A). In the CWG sites, 105 DMS were hypomethylated, and 63 DMS were hypermethylated (Figure 6B). Further analysis reveals that 256 DMS (hypomethylated) were associated with 67 DEGs, whereas 137 DMS (hypermethylated) were associated with 53 DEGs at the CG levels. In addition, at the CWG sites, 105 DMS (hypomethylated) were associated with 22 DEGs, whereas 63 DMS (hypermethylated) were associated with 21 DEGs (Tables S3 and S4). The distribution pattern of the DMS was determined by employing an MA plot (Figure 6C,D), clustering heat map (Figure S4), and volcano plot method (Figure 6E,F) to survey the patterns of the overall DMS. The distribution of the DMS in the chromosomes is shown in Figure S5.

**Figure 7:**
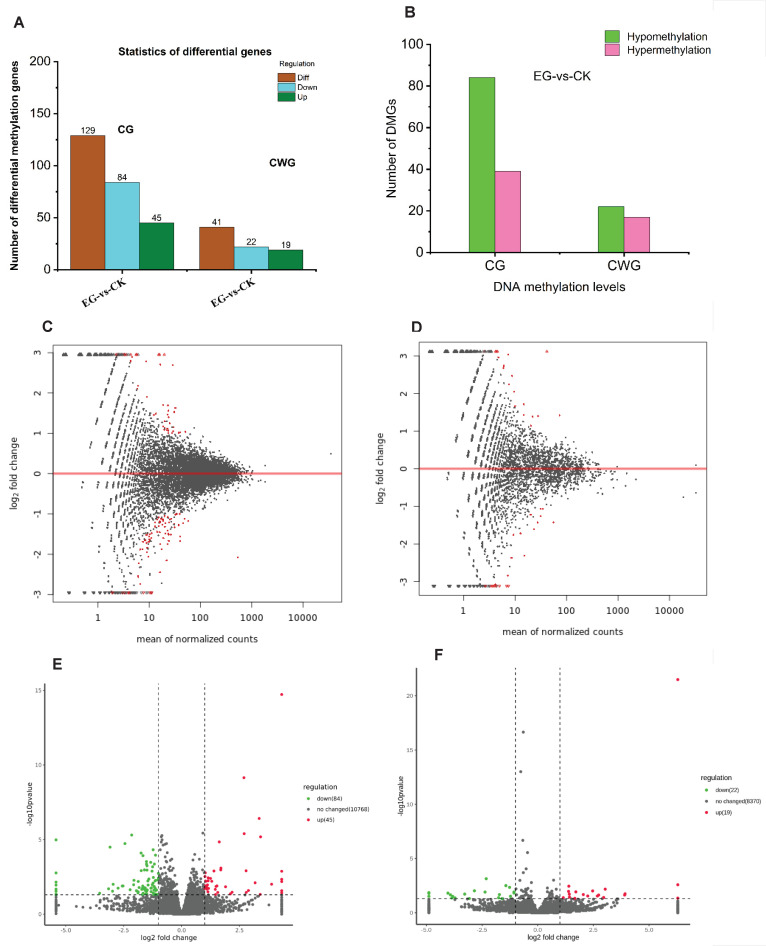
Differential methylation gene at CG and CWG level between EG-vs.-CK. (**A**) The number of differential methylated genes in CG and CWG sites; (**B**) the number of hypo/hypermethylated genes in the CG and CWG sites; (**C**,**D**) MA plot of DMGs in CG and CWG sites; (**E**,**F**) volcano plot of the DMGs in CG and CWG sites. The small red circle represents upregulated DMS. The blue and dark grey color means the downregulated DMS and non-significant methylated sites.

At the gene methylation level, a total of 10,897 and 8411 (Table S4) methylated genes were found in CG and CWG sites, respectively. There were 129 DMGs at the CG sites, comprising 84 downregulated and 45 upregulated. In the CWG sites, 41 DMGs, including 22 downregulated and 19 upregulated, were identified (Figure 7A, Table S4). Interestingly, our results reveal that 84 and 22 DMGs at the CG and CWG sites, respectively, in the EG were hypomethylated. Additionally, 39 and 17 DMGs at the CG and CWG levels, respectively, were hypermethylated (Figure 7B). The genes’ higher hypomethylated status could suggest that DNA methylation may regulate the expression of the genes involved in the mulberry plant growth and development during the drought stress condition. The distribution pattern of the DMGs was determined by employing an MA plot method (Figure 7C,D), volcano plot method (Figure 7E,F), and clustering heat map (Figure S6) to survey the overall DMGs pattern. The distribution of the DMGs on the chromosomes is shown in Figure S7.

### 2.8. The Distribution of DMS in Different Functional Components of the Genome and the Chromosome

**Figure 8:**
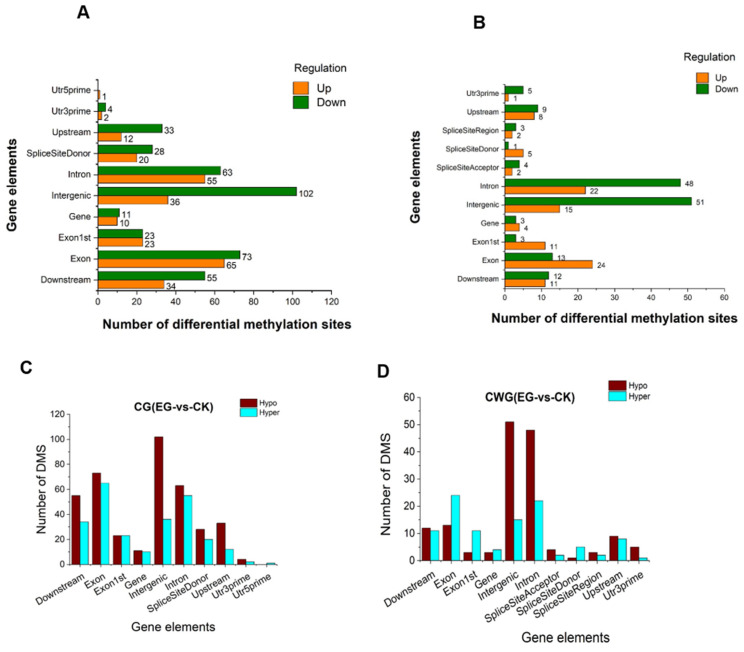
Distribution of differential methylation sites in different gene functional components. (**A**) CG bit with up and downregulation; (**B**) CWG bit with up and downregulation; the horizontal axis denotes the number of DMS; the vertical axis is the gene elements; (**C**,**D**) the differential hyper/hypomethylated site distribution in CG and CWG, respectively.

The distribution of the DMS at the CG and CWG sites in the different gene components of the genome was analyzed using BEDTools software (v2.25.0) set at echo-count -delim parameter [26]. The various genetic components analyzed include utr3prime, utr5prime, exon, intron, intergenic, downstream, 1st exon, gene, splice site acceptor, splice site donor, splice site region, and upstream. Although the distribution of DMS at the CG site in the functional component of the genome was higher than the distribution at the CWG sites, the distribution pattern of the DMS at both the CG and CWG levels was similar. Interestingly, most DMS distribution occurred in the intergenic, exon, downstream, and intron. However, the intergenic region was the most distributed (Figure 8A,B).

Remarkably, the distribution in the utr3prime, utr5prime, however, were significantly lower. The results reveal that most of the DMS at both the CG and CWG levels were downregulated compared to the upregulation. Next, we analyzed the hypomethylated and hypermethylated status of the DMS in the gene elements. Again, we observed that the DMS were more hypomethylated at the CG and CWG context than the hypermethylation (Figure 8C,D). Interestingly, the hypo/hypermethylated sites were abundant in the intergenic, exon, upstream, downstream, and intron regions at the CG sites (Figure 8C). At the CWG site, hypo/hypermethylated sites were abundant in the intergenic and intron regions. However, the DMS were more hypermethylated in the exon region than the hypomethylation (Figure 8D). The higher level of hypomethylated sites in the functional component of the genome during the drought stress suggests that the DNA methylation may have influenced the regulation of the expression of the associated genes for the growth and development in the mulberry plant.

### 2.9. Gene Ontology (GO) Enrichment Analysis of the Genes Associated with DMS

The GO functional enrichment analysis was carried out on the differential expression genes (the list of genes that need to be enriched) where the DMS were located. In total, 8587 coding genes were assigned in the GO terms in both CG and CWG methylation levels (Figure S8A,B; Table S3). Interestingly, 120 of the enriched genes were differentially expressed (DEGs). These 120 genes were significantly associated with the DMS at the CG site and were assigned to 39 highly enriched GO terms (Figure S9A). In addition, 67 of the DEGs enriched in the GO terms were downregulated, whereas 53 were upregulated (Figure S9B,C). On the other hand, 43 DEGs were significantly associated with the DMS at the CWG site and were assigned to 34 highly enriched GO terms (Figure S10A). Interestingly, 22 DEGs involved in the GO terms were downregulated and 21 upregulated (Figure S10B,C). The GO terms at both the CG and CWG sites were classified into three functions: biological process, cellular component, and molecular function.

**Figure 9:**
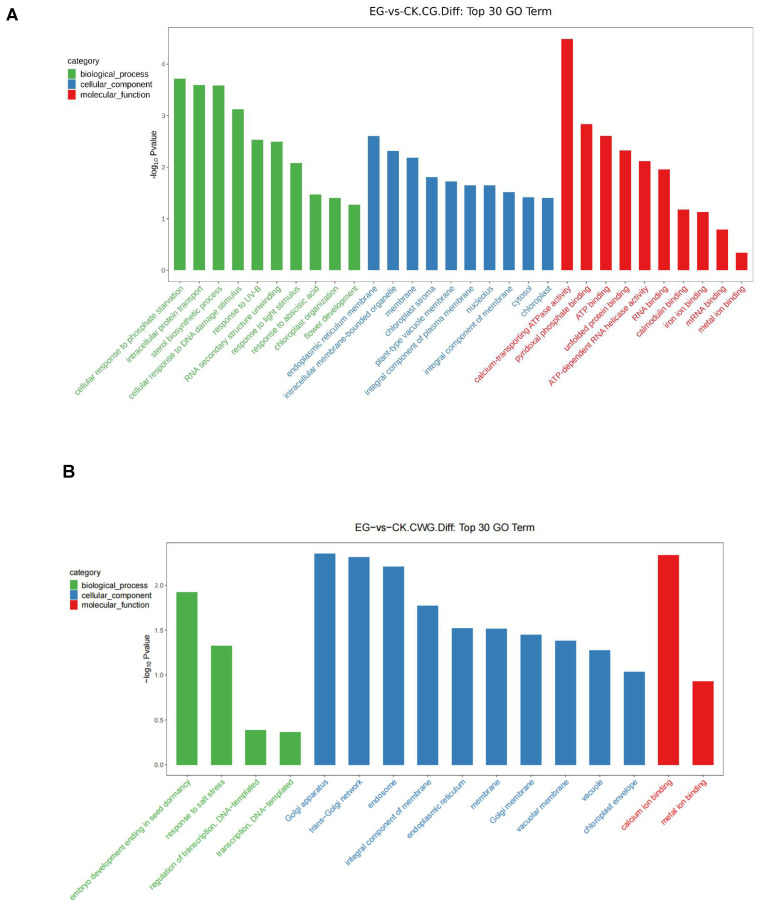
Bar chart of the top 30 GO functions of the genes where CG and CWG differential methylation sites are located. (**A**) The top 30 GO terms at the mCG sites; (**B**) the top 30 GO terms at the mCWG sites. The x coordinates are the name of the GO entry, and the y coordinates are-log10Pvalue. The green, blue and red colors represent biological process, cellular component, and molecular functions, respectively.

We further analyzed the top 30 most significantly enriched GO terms of these DEGs associated with the DMS in CG and CWG methylation levels. The analysis was based on screening GO entries with more than two different expression site-related genes in the three categories, 10 each sorted from large to small according to the corresponding -log10Pvalue for each entry and classified into biological process, cellular component, and molecular function. At the mCG, the most enriched GO terms in the biological process include cellular response to phosphate starvation (GO:0016036), intracellular protein transport (GO:0006886), and sterol biosynthetic process (GO:0016126). The most cellular component includes endoplasmic reticulum membrane (GO:0005789), intracellular membrane-bounded organelle (GO:0043231), and membrane (GO:0016020). In addition, calcium-transporting ATPase activity (GO:0005388), pyridoxal phosphate binding (GO:0030170), and ATP binding (GO:0005524) were the most significantly enriched GO terms in the molecular function (Figure 9A, Table S3). Analysis of up and down regulation reveals that intracellular protein transport (GO:0006886), chloroplast stroma (GO:0009570), and pyridoxal phosphate binding (GO:0030170) were the most significant enriched GO terms in biological process, cellular component, and molecular function, respectively, in the downregulation in the CG levels (Figure S11A, Table S3). In addition, response to abscisic acid (GO:0009737), intracellular membrane-bounded organelle (GO:0043231), and calcium-transporting ATPase activity (GO:0005388) were the most significant enriched GO terms in biological process, cellular component, and molecular function, respectively, in the upregulation of the CG levels (Figure S11B, Table S3).

At the mCWG sites, the most enriched GO terms in the biological process, cellular component, and molecular function were embryo development ending in seed dormancy (GO:0009793), Golgi apparatus (GO:0005794), and calcium ion binding (GO:0005509), respectively (Figure 9B, Table S3). On the other hand, in the downregulated, the most significant enriched GO terms include transcription, DNA-templated (GO:0006351), endoplasmic reticulum (GO:0005783), and metal ion binding (GO:0046872) in biological process, cellular component, and molecular function, respectively (Figure S11C). In addition, Golgi apparatus (GO:0005794) and calcium ion binding (GO:0005509) in cellular component and molecular function, respectively, were the most significantly enriched GO terms in the upregulation mCWG levels (Figure S11D). A comparison of the GO classification where the DEGs were at the up or downregulated in both CG and CWG sites is shown in Figure S12.

### 2.10. KEGG Pathway Analysis of the DEGs Associated with DMS

The KEGG pathway analysis was employed to explore the biological pathway and the signal transduction of the DEGs associated with the DMS at the CG and CWG methylation levels. Figure S13 shows the classification of all genes and the DEGs at the CG and CWG methylation level mainly involved in the KEGG pathway. The analysis of the KEGG pathways shows that 98 DEGs, including 35 in the upregulation mCG and 63 in the downregulation mCG sites, were highly involved in 21 pathways (Figure S14A). In the upregulated mCG sites, the DEGs were mainly involved in the 18 KEGG pathway and classified into cellular process, environmental information process, genetic information process, and metabolism. However, the DEGs were more involved in the metabolism pathway. The most significant pathways include lipid and carbohydrate metabolism (Figure S14B). furthermore, in the downregulated mCG sites, the DEGs were mostly involved in 17 KEGG pathways, and the most significant pathway involved metabolism (Figure S14C). At the CWG sites, 39 DEGs (13 in the upregulation mCWG and 26 in the downregulation mCWG sites) were highly involved in 17 KEGG pathways (Figure S15A). The most significant pathway involving the DEGs includes translation, signal transduction, and cell growth and death (Figure S15B,C).

**Figure 10:**
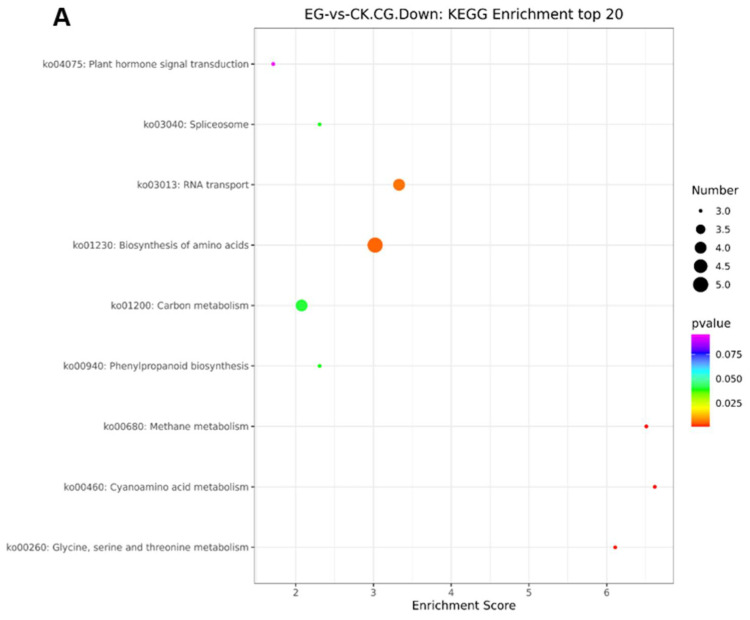

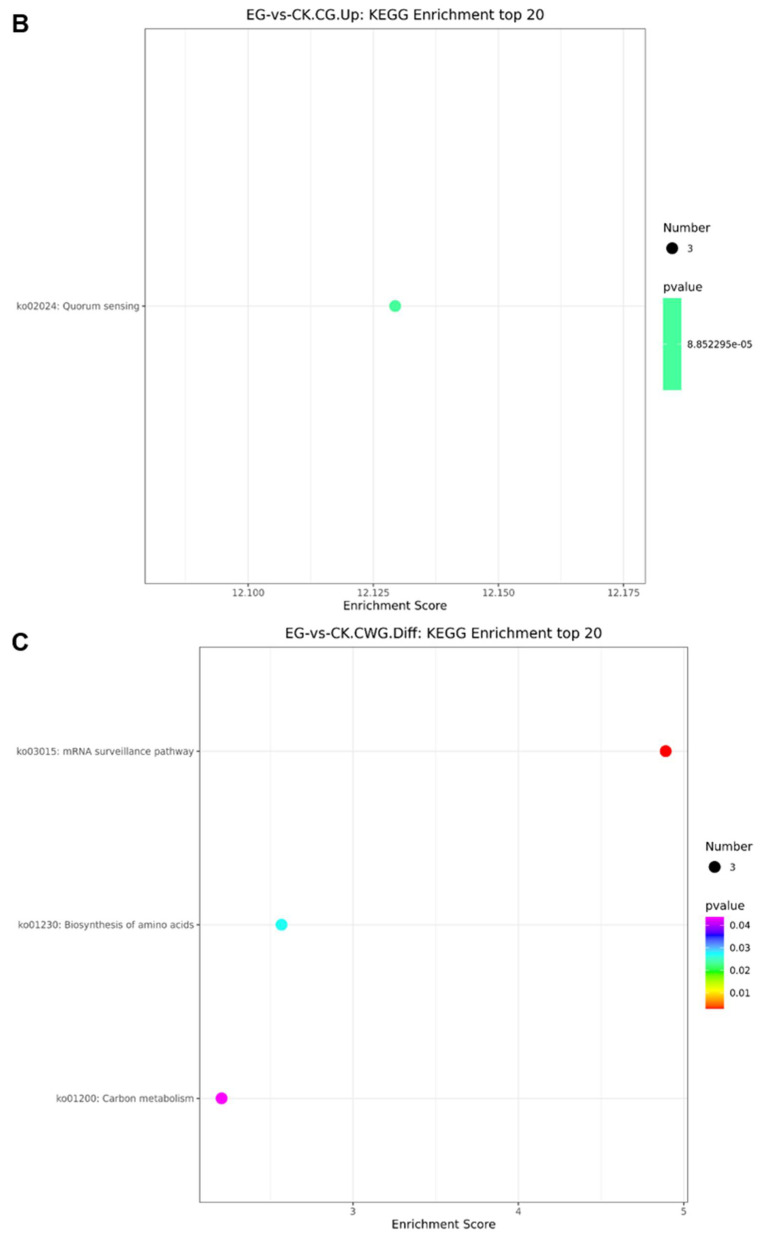
The top 20 KEGG enrichment analyses of the genes where the CG and CWG differential methylation sites are located. (**A**,**B**) Differential KEGG enrichment at the CG methylation level. (**C**) Differential KEGG enrichment at the CWG methylation level. The *x*-axis is the enrichment score, and the *y*-axis is the KEGG enrichment. The bubble represents gene entries; the larger the bubble, the more entries it contains and the more differential protein-coding genes. The bubble color changes from purple-blue-green-red, denoting a p-value and the smaller the enrichment value, the greater the significance.

The DEGs involved in the top 20 enrichment pathways at the CG and CWG sites are shown in Figure 10. The top 20 enrichment pathways screening was based on *p*-values less than 0.05. The results show that at the CG site, the pathways were mainly related to spliceosome (ko03040), carbon metabolism (ko01200), plant hormone signal transduction (ko04075), biosynthesis of amino acids (ko01230), RNA transport (ko03013), glycine, serine and threonine metabolism (ko00260), and quorum sensing (ko02024) (Figure 10A,B). At the CWG site, the top 20 KEGG enrichment pathways were mainly involved in the mRNA surveillance pathway (ko03015), biosynthesis of amino acids (ko01230), and carbon metabolism (ko01200) (Figure 10C).

### 2.11. Gene Ontology (GO) Enrichment Analysis of the DMGs

The DMGS function was analyzed in the EG-vs.-CK to determine the DMGs significantly enriched in the GO terms. The total coding genes and the DMGs enriched in the GO terms at the CG and CWG level are shown in Figure S16A,B. At the mCG sites, 49 DMGs were significantly assigned to 36 highly enriched GO terms classified into biological process, cellular component, and molecular function (Figure S17A, Table S4). Interestingly, 30 DMGs in the GO terms were at the downregulated mCG level, whereas 19 DMGs were at the upregulated mCG level (Figure S17B,C). At the mCWG sites, 16 DMGs were significantly assigned to 26 highly enriched GO terms (Figure S16D, Table S4). However, 8 DMGs were involved in the up and downregulated CWG methylation level (Figure S17E,F). The GO term classification where the DMGs were at the up and downregulated mCG and mCWG sites are shown in (Figure S18).

**Figure 11:**
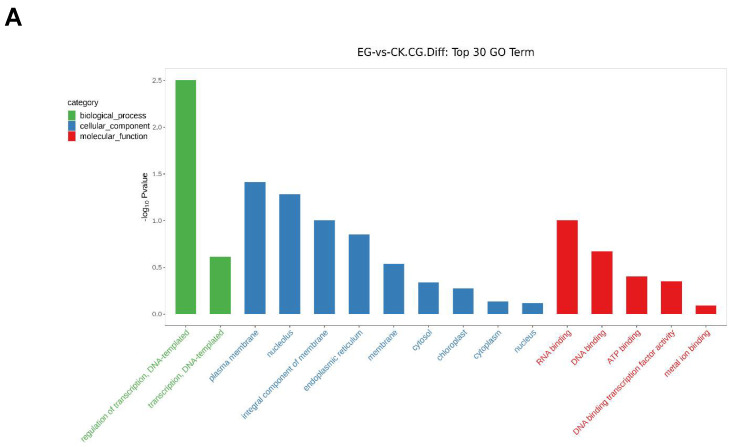

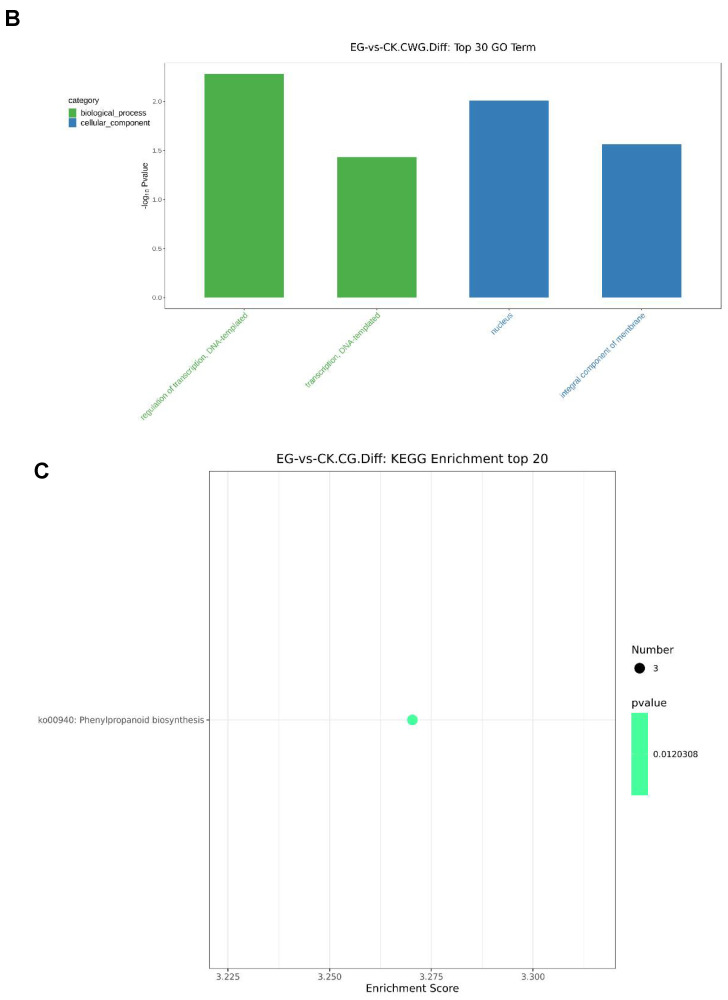
The top 30 GO functions and the top 20 KEGG of the DMGs at the mCG and mCWG sites. (**A**) The top 30 differentials GO terms at mCG sites; (**B**) the top 30 differentials GO terms at mCG sites; The X coordinates in the figure are the name of the GO entry, and the Y coordinates are −log10P value. The green, blue and red colors represent biological process, cellular component, and molecular functions, respectively, (**C**) the top 20 KEGG enrichment. The *x*-axis is the enrichment score, and the *y*-axis is the KEGG enrichment. The bubble represents gene entries; the larger the bubble, the more entries it contains and the more differential protein-coding genes. The bubble color green denotes the p-value, and the smaller the enrichment value, the greater the significance.

The top 30 GO term analysis shows that regulation of transcription, DNA-templated (GO:0006355), plasma membrane (GO:0005886), and RNA binding (GO:0003723) were the most significantly enriched GO terms in biological processes, cellular component, and molecular function, respectively at the mCG sites (Figure 11A). Interestingly, most of the DMGs involved in the GO terms were associated with downregulated mCG sites compared to the upregulated mCG sites (Figure S19A,B). At the mCWG sites, the DMGs significantly enriched in the GO terms were related to the regulation of transcription, DNA-templated (GO:0006355), and nucleus (GO:0005634) classified into biological processes and cellular component respectively (Figure 11B). Interestingly, at the downregulated mCWG sites, the DMGs were significantly enriched in the integral component of membrane in the cellular component of the GO terms, and in the mCWG upregulated sites, the DMGs were significantly enriched in regulation of transcription, DNA-templated, and nucleus in the biological process and cellular components, respectively (Figure S19C,D).

### 2.12. KEGG Pathway Analysis of the DMGs at the mCG and mCWG Sites

The KEGG pathway analysis involving the DMGs and all genes annotated to the KEGG pathways at the mCG and mCWG sites are shown in Figure S20. At the mCG site, 38 DMGs (25 in the downregulated mCG sites and 13 in the upregulated mCG sites) were significantly enriched in 20 highly KEGG pathways (Table S4). Remarkably, most of the DMGs were involved in metabolism pathway (including amino acid metabolism, metabolism of terpenoids, biosynthesis of other secondary metabolism, and carbohydrate metabolism), genetic information processing (including replication and repair, folding, sorting, and degradation), and environmental information processing including signal transduction and membrane transport (Figure S21A–C). On the other hand, 11 DMGs were enriched in 9 KEGG pathways and classified into environmental information, genetic information, and metabolism in the mCWG sites. Interestingly, all the DMGs involved in the KEGG pathway were at the downregulated mCWG sites and were mainly implicated in the metabolism pathway, including amino acids, carbohydrates (Figure S21D,E). The top 20 enrichment pathways analysis shows that the enrichment pathways were only related to the CG methylation site and the genes implicated in phenylpropanoid biosynthesis pathways (ko00940) (Figure 11C).

### 2.13. Analysis of Differential DNA Methylation at the Promoter Gene (DMPGs) Level

The promoter regions of genes affect transcriptional regulation, and thus, the differential methylation of promoters may affect transcriptional expression. To reveal the changes in the methylation status of the promoter genes in the mulberry subjected to drought stress and the control, we analyzed the mCG and mCWG at the promoter gene-level located at the 2-kb upstream level of the gene starting site. We found 3515 and 1887 methylated genes at the mCG and mCWG sites, respectively (Table S5). The promoter level presented a higher CG methylation level than the CWG level. Analysis of the differential methylation genes at the promoter identified 28 DMPGs (20 down- and 8 upregulated) and 17 DMPGs (7 down- and 10 upregulated) at mCG and mCWG sites, respectively between EG and CK (Figure S22, Table S5). In addition, we discovered that 6 DMPGs at the CG sites were hypermethylated, whereas 20 were hypomethylated.

On the other hand, 7 hypermethylated and 7 hypomethylated DMPGs were identified at the CWG sites. The GO function of the DMPGs was analyzed. Interesting 5 DMPGs were involved in the GO terms and classified into biological precesses (included; cellular process, biological regulation, and metabolic process), cellular component (included; cell, cell part, and organelle), and molecular function (included; binding, and catalytic activity) (Figure S23A). However, 3 DMPGs were mainly involved in cellular components at the downregulated mCG sites, whereas 2 were more into biological processes at the upregulated mCG sites (Figure S23B,C). At the mCWG sites, 6 DMPGs were enriched in the GO terms, and they were mainly assigned to biological processes, cellular components, and molecular functions, including cell parts and biological regulation and binding (Figure S23D). Only 1 DMPG in the downregulated mCWG sites was involved in the GO terms. In contrast, the remaining 5 were in the upregulated CWG GO terms (Figure S23E,F). The KEGG analysis reveals that the DMPGs were related to the carbohydrate metabolism pathway at the mCG level. In addition, the DMPGs were related to folding, sorting and degradation pathway, carbohydrate metabolism, and translation pathway at the mCWG level (Figure S24A,B).

### 2.14. Validation of DMGs and DEGs Associated with DMS by Quantitative Real-Time PCR

**Figure 12:**
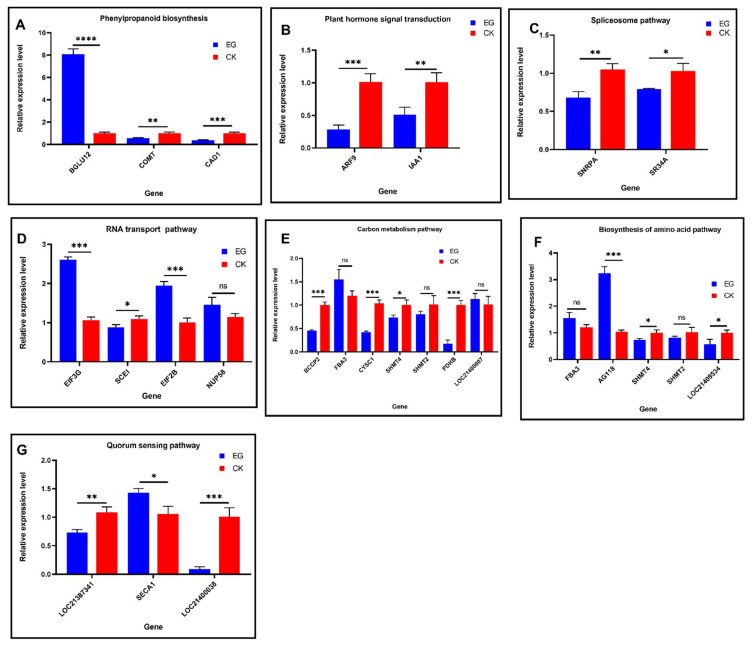
qRT-PCR results of DMGs and DEGs in the KEGG enrichment pathways. From (**A**–**G**), represent the various KEGG pathways. Mulberry actin3 gene was used as the internal control gene. The mean value ± SD was used to analyze the relative transcript level of each gene by the 2^−ΔΔCt^ method. The qRT-PCR reactions were run with three biological replicates in three technical replicates. Statistical analysis was performed using unpaired Student’s t-test by GraphPad Prism9 version 9.0.0 for Windows, GraphPad Software, San Diego, CA, USA. A *p* < 0.05 was considered significant. * *p* < 0.05, ** *p* < 0.01, *** *p* < 0.002, **** *p* < 0.0002, ns = not significant.

To confirm the correlation between the methylation variation of differentially methylated genes (DMGs) detected by MethylRAD-seq and the genes (that were enriched in the GO terms and KEGG) associated with the differentially methylated site, and their gene expression levels under drought stress, we performed qRT-PCR analysis using three replicates to assess 23 genes associated with the phenylpropanoid biosynthesis, spliceosome, biosynthesis of amino acid, carbon metabolism, RNA transport, plant hormone, signal transduction pathways, and quorum sensing. The results indicated that these genes were significantly differentially expressed, suggesting that the DNA methylation status might be regulating these genes under the drought stress condition (Figure 12).

## 3. Discussion

Studying DNA methylation patterns across plants’ entire genome has been significant. The ongoing development of high-throughput sequencing technologies and array-based methods allows studying DNA methylation patterns across entire genomes. DNA methylation is a significant epigenetic modification and has been widely implicated in plant development and stress responses [29]. MethylRAD sequencing is a powerful method that determines methylation patterns in plants [4]. In this study, the MethylRAD-seq method was performed to evaluate genome-wide DNA methylation patterns in mulberry leaves and identify important differentially methylated genes and differentially methylated sites and their associated genes in response to drought stress. DNA isolated from the drought stress and control leaves were used to construct four libraries and sequenced using the Illumina Hiseq X Ten on a Nova PE150 platform.

Our results indicate that CG methylation patterns occurred more (37.37%) than the CWG (28.81%) in the mulberry plant genome under the drought stress and control treatment. Proportionally, there was a slight increase in the mCG sites (9.44%) under the drought stress than the control (9.25%). In addition, a slight increase in the proportion of the mCWG occurred under the drought stress (7.37%) compared to the control (7.05%), suggesting that drought stress induces more DNA methylation in the mulberry genome. Earlier studies demonstrated that mCG sites show the highest levels among the various species, ranging from ~30.5% in Arabidopsis to ~92.5% in *Beta vulgaris*. mCHG methylation varied from ~9.3% in *Eutrema salsugineum* to ~81.2% in *Beta vulgaris*, and mCHH methylation ranged from ~1.1% in *Vitis vinifera* to ~18.8% in *Beta vulgaris* [2,22,30]. Our findings are consistent with prior methylome research, which suggested that mCG methylation is the most abundant of the three forms of methylation, while mCHG and mCHH methylation levels are often lower [2,31,32]. In addition, these findings indicate that drought stress increased methylation in two sequence contexts, mCG and mCWG, and that drought stress resulted in hyper- and hypomethylation patterns, which is consistent with earlier research [31].

DNA methylation level varies according to the region of the genome being examined. CG methylation is frequently detected within genes and repetitive regions and linked to gene expression regulation [33]. Apart from CG, mCHG and mCHH methylation are uncommon within genes and are more abundant in intergenic and repetitive regions of the genome [33]. Methylation within these sequence regions is crucial for transposon silencing [34]. In the present study, we observed that CG and CWG sites’ methylation level was higher in the intergenic, exon, intron, and downstream regions of the mulberry genome. However, the level of the methylome was lower in Utr3prime, Utr5prime, splice site region, and splice site acceptor region of the genome and gene region. Earlier works reported that the distribution of DNA methylation was predominant in the intergenic, exon, intron, downstream, and upstream regions, and lower at gene, 1st exon region of the genome [16,35]. Our data also support other research, which indicates that the mCG and mCHG were lower at the utr5prime [34]. In addition, further research reports that DNA methylation status is more common in the gene body [36,37]. However, in our study, the methylation level at the gene body was less than the TSS and the TTS.

It is reported that the CG methylation level occurs more at the gene promoter level than at the mCWG level [2]. Our results found that the methylation levels at the CG context are more than the CWG at the gene promoter, which is consistent with the earlier report [2]. Generally, DNA methylation in the promoters is associated with genes involved in transcriptional repression or silencing [38]. Nevertheless, more recent data suggest that highly methylated promoters can also be found in upregulated genes [39]. Our results discovered 3515 and 1887 DNA methylated genes at the promoter level in CG and CWG sites, respectively. However, only 28 DMGs at the promoter level were linked to 20 downregulated and 8 upregulated CG methylation sites, and 17 DMGs at the promoter level comprising 7 downregulated and 10 upregulated at the CWG methylation site were identified. These results imply that a small proportion of DMGs exhibited differentially significant methylation expression levels at the promoter level. These findings support prior research indicating that, in most situations, changes in gene expression levels are not associated with differences in DNA methylation [40,41]. Gene ontology analysis of DMGs at the promoter level found that these genes were primarily involved in cellular component organization or biogenesis, cell, and binding.

Abiotic stress responses in plants are mediated by changes in DNA methylation across the plant’s genome [36]. This work identified 170 DMGs (including 129 in CG sites and 41 in CWG sites) between the drought stress and control. The genes were mainly assigned to three categories of GO terms, including biological processes, cellular components, and molecular function. These functions include regulation of transcription, DNA templated, plasma membrane, and RNA/DNA binding. The result shows that drought stress-induced methylation variation resistance genes.

Further KEGG pathway enrichment analysis of the DMGs reveals that the phenylpropanoid biosynthesis pathway plays a crucial role in drought stress response in mulberry (Figure 11). Our previous study demonstrated that mulberry plant, Yu-711 exposure to drought stress altered phenylpropanoid metabolites, an important class of compounds that protect plants from various stressors [10]. In this present study, genes such as BGLU12, COMT, and CAD1 encode for beta-glucoside 12 like, caffeic acid 3-O methyltransferase and probable mannitol dehydrogenase protein, respectively, were implicated in the phenylpropanoid biosynthesis pathway.

The enzyme beta-glucosidase (BGLU) catalyzes the hydrolysis of beta-D-glucosidic bonds by releasing glucose, which is essential for the liberation of numerous physiologically significant molecules [42]. BGLUs gene family plays a role in various plant functions, including the timely response to biotic and abiotic stressors via the activation of phytohormones and defense compounds [42]. Plant caffeic acid 3-O-methyltransferase (COMT) has been involved in the lignin biosynthesis process by catalyzing the multi-step methylation of hydroxylated monomeric lignin precursors [43]. Lignin is a structural heteropolymer found in high concentrations in vascular plants. It protects plant tissues from biotic and abiotic stressors by providing intercellular hydrophobicity and mechanical support [44]. These authors indicate that the level of COMT expression decreased under drought stress conditions and led to an increase of lignin content in *Brassica napus* L. [44].

According to Wang and colleagues, COMT and CAD1 can potentially act in various branches of the phenylpropanoid pathway [43]. In this work, we found that BGLU12, COMT, and CAD1 shared decreased methylation status. However, qRT-PCR analysis reveals that the gene expression level of COMT and CAD1 decrease substantially under the drought stress condition (Figure 12A), which may indicate that COMT and CAD1 could be responsible for the synthesis of a specific subunit of lignin. Interestingly, the expression level of BGLU12 was significantly upregulated by about 8-folds compared to the control, implying that DNA methylation might have induced regulation of BGLU12 in the mulberry plant in response to drought stress by activating defense compounds and phytohormones [42]. Furthermore, the genes involved in the phenylpropanoid pathway agree with other studies [43,45].

The analysis of the DMS shows that the DMS was most prevalent between the control and drought stress conditions. The results show that 581 DMS was identified (including 413 in CG site and 168 in CWG site). Further analysis reveals that the hypo/hypermethylation shares 67/53 DEGs at the mCG site. On the other hand, hypo/hypermethylation at the mCWG site shares 22/21 DEGs. GO analysis reveals that these genes associated with the DMS are mainly classified into three GO terms; biological process, cellular component, and molecular function. These functions include cellular response to phosphate starvation, endoplasmic reticulum membrane, and calcium-transporting ATPase activity at the CG methylation site. In contrast, embryo development ending in seed dormancy, Golgi apparatus, and calcium ion binding are the GO functions at the CWG methylation site. Furthermore, KEGG pathway enrichment analysis on the DEGs associated with the DMS reveals that plant hormone signal transduction, spliceosome, carbon metabolism, RNA transport, biosynthesis of amino acid, and quorum sensing pathways play a key role in drought stress response in mulberry (Figure 10).

Hormone signaling is an important biological process as it induces specific transcriptional changes in eukaryotic organisms. Interestingly, in this work, genes such as ARF9, IAA1, and PP2C53 were implicated in plant hormone signal transduction. There has been evidence of a close link between epigenetic regulation and plant hormone transduction [35]. Phytohormones are involved in compacting chromatin, mediated by DNA methylation and histidine modification [35]. The plant’s most critical hormone signal pathway is auxin, which functions throughout plant development, acting embryonically, post-embryonically [46]. Auxin response factors (ARFs) are plant-specific transcription factors (TFs) that couple perceptions of the hormone auxin to gene expression programs via a series of functionally different ARFs that bind to DNA [46,47]. At low auxin concentrations, Aux/IAAs physically interact with specific ARFs, inhibiting their target gene expression; however, at high auxin concentrations, auxin promotes the binding of Aux/IAAs to SCFTIR1/AFB E3 ligases, resulting in the degradation of the transcriptionally repressive Aux/IAAs and allowing certain ARFs to activate downstream target genes [48]. In this work, ARF9, IAA1, and PP2C53, hormone signal transduction genes, shared downregulated DNA methylation levels. However, the qRT-PCR analysis reveals the expression level of the genes decreased significantly (Figure 12B), implying that Aux/IAAs physically might interact with specific ARFs, inhibiting their target gene expression under DNA methylation in response to drought stress [48].

Spliceosomes are large ribonucleoprotein complexes made up of numerous proteins and small nuclear RNAs found primarily in the nucleus of eukaryotic cells [49]. They remove introns from pre-mRNA to form a matured mRNA, a type of primary transcript after it has been transcribed [49]. Alternative splicing (AS) is a post-transcriptional regulatory mechanism for increasing proteome diversity by modulating gene expression [50]. Evidence reveals that under drought stress conditions, genes undergo AS to enable the formation of different mRNA isoforms due to alternative ways of pre-mRNA processing [50]. In this work, genes such as SR34A, ESP3, and SNRPA encode for serine/arginine-rich splicing, Pre-mRNA-splicing factor ATP-dependent RNA helicase DEAH1, and small nuclear ribonucleoprotein polypeptide A proteins, respectively, were significantly enriched in the spliceosome pathway. Furthermore, these genes shared a decreased DNA methylation status. However, the genes expression levels increased (Figure 12C), implying that drought stress enhances RNA splicing. These further suggest that drought stress conditions might have induced alternative splicing (AS) by decreasing the methylation state of genes involved in RNA splicing [32].

Translational regulation is a crucial phase in gene expression regulation. In plants, translation regulation is critical at all stages of development [51]. It serves as a prompt and versatile mechanism that modifies the global translation rate and controls the production of specific proteins during stress responses [51]. We found that genes including SCEI, NUP58, eIF3G, and eIF2B encode for SUMO-conjugate enzyme protein, eukaryotic translation initiation factor 3 subunit G-like protein, and eukaryotic translation initiation factor 2 subunit beta protein, respectively, were enriched in the RNA transport pathway. In addition, the nuclear pore complex (NPC), composed of separate nucleoporin (Nup) proteins, regulates the nucleo-cytoplasmic transport of RNA and protein and is critical for plant growth and development regulation [52].

Moreover, sumoylation played a critical role in stress responses in higher plants as a key regulatory mechanism of post-translational modifications [53]. Under drought and salt stress, SCEI expression level was reported to increase, modulating sumoylation levels, antioxidant capability, and stress defense gene expression to enhance plant growth and development [53]. While each translation step can be regulated, most regulatory mechanisms are concentrated in the initiation phase, where many translation initiation factors (eIFs) enable mRNA-ribosome connection, mRNA scanning, and start codon selection [51]. eIF2B has been reported to function as a small GTPase, forming a ternary complex with GTP and Met-tRNAi, and eIF3G functions as a pre-initiation complex (PIC), scanning and AUG recognition, mRNA joining [54]. In this work, SCEI, NUP58, eIF3G, and eIF2B shared downregulated methylation status. Relative expression analysis confirmed that these genes increased expression during the drought stress condition (Figure 12D), indicating that these genes were induced under drought stress and might have played a vital role in the post-translational modifications.

Carbon metabolism is essential for plants because it provides most of their energy and essential nutrients. However, environmental cues such as drought significantly impact carbon metabolism and thus plant growth [55]. Carbon metabolism encompasses photosynthetic carbon assimilation, sucrose, and starch metabolism, as well as carbohydrate transport and utilization [55]. The rate and efficiency of photosynthesis play a significant role in determining plant productivity. In this study, most of the genes in the KEGG enrichment analysis are involved in the carbon metabolism pathways. Genes such as FBA3, SHMT2, SHMT4, BCCP2, CYSC1, PDHB, and LOC21400607 were enriched in the carbon metabolism pathway. From the MethylRAD sequencing data, these genes share a decreased methylation.

Additionally, qRT-PCR analysis of these genes shows FBA3, SHMTs and LOC21400607 have an increased expression level, whereas BCCP2, CYSC1, PDHB expression levels decreased under the drought condition (Figure 12E). Fructose-1, 6-bisphosphate aldolase (FBA), is an essential plant enzyme that participates in glycolysis, gluconeogenesis, and the Calvin cycle. These reactions are necessary for carbon fixation and sucrose metabolism in green plants, chloroplast stroma, and cytosol and are involved in biotic and abiotic stress responses [56]. The expression level of FBA under various stress conditions such as cold, drought, and salt has been reported to induce both up and downregulation [56]. In this work, the FBA3 gene was significantly upregulated under drought stress, suggesting that DNA methylation may have induced the expression level of the gene to increase the energy level of the mulberry plant during the stress period. Our results agree with the findings by these authors [56]. While one-carbon metabolism produces essential cellular components such as nucleotides, lipids, and proteins for cell development, it also generates glutathione and S-adenosylmethione, which are required to maintain cells’ cellular redox and epigenetic status [57]. Serine hydroxymethyltransferase (SHMTs) are key serine/glycine conversion enzymes.

SHMTs enzyme catalyzes the reversible conversion of serine to glycine by transferring the β-carbon of serine to tetrahydrofolate (THF), resulting in the synthesis of 5,10-methylene-THF and glycine; both of these compounds are engaged in the folate cycle [57]. The SHMT2 gene mediates the conversion of serine and glycine in the human genome, and the expression level was upregulated [57]. In plants, SHMT has been reported to be downregulated in response to drought stress in buckwheat [58]. This study found that SHMT2 gene expression levels increase significantly during drought conditions compared to the control (Figure 12). Additionally, the SHMT4 level was less compared to the control. However, the expression level increased appreciably. On the other hand, the expression level of BCCP2 decreased under drought stress which is consistent with other studies [59]. All together, indicating these genes may have played a vital role in the plant adaptation to drought stress.

Biosynthesis of amino acid levels is essential for plant stress tolerance by acting as osmolytes, precursors for energy-associated metabolites, ROS scavengers, and potential regulatory and signaling molecules [60]. In the amino acid pathway, genes such as AG118, LOC21409534, FBA3, SHMT2, and SHMT4 were significantly involved (Figure 12F). Furthermore, relative expression analysis revealed that all the genes involved amino acid pathway increased in expression. Notable are AG118, FBA3, and SHMT2. The acetylornithine aminotransferase (AG118) gene expression level increased to about 3.4-fold compared to the control. Interestingly, all these genes share downregulated methylated status, and their expression level increased, suggesting the DNA methylation variations induce these genes and may involve in osmolytes, precursors for energy-associated metabolites, and ROS scavengers [60].

Quorum sensing (QS) molecules are one of the key mechanisms bacteria communicate. Plants also evolve and react to these molecules [61]. Arabidopsis and Wheat plant treatment with N-acyl hormoserine lactones (AHL), a notable QS molecule, promoted plant defense mechanism, growth and development, and tolerance to salt stress and biotic stress [61,62]. In this work, the quorum-sensing pathway was significantly enriched during the mulberry plant exposure to drought stress. Genes such as SECA1, LOC21400038, and LOC21387341 encoding protein translocase subunit SECA1, probable pectate lyase 18, and an uncharacterized protein, respectively. Interestingly, all these genes shares upregulated DNA methylation status from the MethylRAD data. However, the qRT-PCR analysis results show that SECA1 was upregulated in the drought leaves. On the other hand, the SECA1 protein expression level has been reported to be downregulated under heat stress [63].

Additionally, pectate lyase 18 (LOC21400038) expression level was significantly lower (Figure 12G). Pectate lyase is involved in cell wall modification, and formation of root structures and downregulation of pectase layse response to drought stress has been recently reported, which agrees with our work [64]. Finally, though the level of the uncharacterized gene (LOC21387341) expression in the drought stress was less than in control, its expression level somehow increased appreciably. Thus, the functional analysis of this gene needs further investigation. Altogether, these results suggest that DNA methylation may have regulated these genes in response to the drought stress.

## 4. Materials and Methods

### 4.1. Plant Materials and Treatments

The mulberry species (*Morus alba*) Yu-711 was obtained from the National Mulberry GenBank at the Jiangsu University of Science and Technology, Zhenjiang, Jiangsu, China. Plants were grown in a greenhouse with a 14 h light/10 h dark photoperiod, at 25 °C day/20 °C night temperature, and relative humidity of 70–80% based on the previous study [10]. The cuttings were grafted to the rootstocks. The grafted nurseries, reaching the three-leaf stage, were planted in pots of 35 cm diameter containing loam soil with one seedling per pot. A total of 18 pots were grouped into drought and control. Each group containing nine pots were divided into three replicates, with each replicate made up of three pots. The control and drought groups were watered daily until new shoots reached 20 cm growth for approximately two months. The drought stress experiment began upon the emergence of fresh leaves after the seedlings development. Water supply was withdrawn for 14 days in one group to induce natural drought stress. However, the control group was constantly supplied with water daily. Leaves were sampled when the drought-stress experimental seedlings reached the wilting point (symptoms apparent) (Figure 1).

The first three-time point for sampling after 14 days of drought stress was the first day (1 day), the third day (3 days), and the fifth day (5 days). The control and drought-treated plants were sampled simultaneously (midday). The primary leaf tissue samples were harvested and immediately frozen in liquid nitrogen and stored at −80 °C. Leaves from stressed experiment and control groups from the 5 days time point (*n* = 4) were used for genomic DNA isolation and MethylRAD library construction and sequencing.

### 4.2. Genome DNA Isolation, MethylRAD Library Construction, and Sequencing

Genomic DNA in leaves was isolated using a DNAsecure Plant Kit (OE Biotech Co., Ltd., Shanghai, China) following the manufacturer’s protocol. First, the DNA quality was checked on 1% agarose gel electrophoresis. Next, four MethylRAD libraries were created from the DNA isolated from the drought stress group (EG) and the control group (CK) treatment. The DNA (200 ng) from the various samples were digested with 5U FspEI (New England BioLabs, cat. no. R0662L, Ipswich, MA, USA) in a 15 µL reaction for 4 h at 37 °C and subjected to 1% agarose gel electrophoresis, producing 32-bp fragments, including four-base 3’overhangs [4]. After the digestion, the DNA fragments were further used for adaptor ligation at 4 °C for overnight [4]. Finally, the ligated PCR was amplified with index primers and purified using a QIAquick PCR purification kit (Qiagen, cat. no. 28106, Hilden, Germany). Sample-specific barcodes were incorporated in each construct by PCR, and the products were sequenced using an Illumina Hiseq X Ten Nova PE150 platform. The primers used for the PCR are listed in Table S6.

### 4.3. Quality Control and Alignment to Reference Genome

Sequencing with the Nova-seq PE150 platform was performed on the 5 days time point samples from the drought and control leaves. The Phred score approach was used to calculate the base quality values. The data quality was checked using checkfastq v. 0.1.0 using the default parameters before and after quality trimming and adapter removal. To improve the accuracy of the analysis, the Raw Reads were filtered again according to the following criteria; (i) normalization of all sample data volumes, (ii) fragment insertion extraction according to the sequence of primer connectors, (iii) fragments with enzyme (FspEI) cutting sites were kept, (iv) fragments with enzymatic cutting point distance of 5’ or 3’ end 13–17 bp were retained and (v) high- quality fragment (those with a mass value of more than 80% of the base and less than 8% of N base content) were retained.

To take advantage of the individual’s clean read, finally, Bowtie2 (version 2.3.4.3) [24] with the -no-unal parameters was used to map the clean reads to the mulberry notabilis reference genome, ASM41409v2. A maximum of one mismatch was allowed in read mapping. Reads mapped to the genome exactly one time were included in the subsequent analysis as it was impossible to judge where the methylation site’s definite position occurred in the multi-mapped reads.

### 4.4. Methylation Site Identification and Quantification

Methylated sites were classified by iterating through all the read sequences to find a matched pattern of CG/CWG methylation sites (where W = A or T bases, i.e., CCGG, CCAGG, and CCTGG) and their location in the genome was recorded. Adjustment for substitution, deletion, and insertion was performed after the number of high-quality reads mapped to each methylated site was recorded. Sites were also matched with the reference genome for verification. Sites with fewer than five reads were removed from the downstream analysis as these were less reliable. Counts from duplicate sites between patterns were summed as one site. The observed sequencing depth in MethylRAD data directly corresponded to the degree of methylation at the site, with a higher depth indicating a higher methylation level. To determine the relative quantification of the MethylRAD data, normalized data (i.e., reads per million, RPM) was employed to calculate the levels of each restriction site CG/CWG. Genome-wide DNA methylation patterns were obtained by summarizing the mean methylation level of each 100 kb window across the genome.

### 4.5. Comparison of Methylation Levels and Correlation between Samples

The methylation levels in the drought and control treatment samples were determined. The sequencing depth of the methylation label can reflect the methylation level of the site. Therefore, a higher label depth indicates a higher level of methylation. The unit of the methylation level quantification value in each sample was determined by a normalized read depth-reads per million (RPM; equal to read coverage per site/high-quality reads per library × 1,000,000). Quartiles were used to determine the distribution of methylated sites regions with the most significant loss of methylation and greatest gain of methylation between drought and control samples. The distribution of the methylated sites throughout the genome was determined by the number of methylation sites (actual number of sites), the number of electron enzyme tangents (the number of theoretical sites), and the reads depth (the sum of the depths of the inner bits in the window) were counted as sliding windows so that the distribution of such sites on the entire chromosome can be described.

Here, the 10 kbp area was used for the window, moving 5 kbp steps each time. Circos v0.69.6 with the default parameters [25] was used to draw a line chart of the frequency distribution. The methylation levels of different samples were reflected in sequencing depth, and the methylation levels of both samples were compared. The sequencing depth scatterplot of all methylation sites was plotted. The Pearson’s correlation coefficient was calculated to determine the correlation between the samples.

### 4.6. Methylation Site Horizontal Genomic Annotation

Based on the location information of the methylation sites, snpEff v4.1g [27] was used to predict the details of the gene elements in terms of location on the genome and the description. The distribution of the methylation site in the different genetic components of each sample in the genome was annotated using BEDTools v2.25.0 [26]. The number of methylation sites in different functional elements of the mulberry genome (such as promoters, gene body, exon, intron, intergenic regions, and downstream regions, upstream region, 3’UTR, 5’UTR) was analyzed in this study. In addition, the distribution of methylation sites in the transcription starting position (TSS), gene body, and the transcription termination location (TTS) regions was carried out. A 2-kb segment upstream of the gene TSS, TTS, and the gene body were selected to analyze the sequence and the distribution trend line chart of the reads in the above segment. In the gene body, we divided the sequence of each gene into 100 windows, counted the RPM values for each window, and then averaged the RPM values of all genes in the same window as the RPM values for that window. With the TSS and TTS, we divided the 2 kb segment into 101 windows, counted the RPM values for each window, and averaged the same window’s RPM values for all segments as the RPM values for that window.

### 4.7. Differential Methylation at the Site and Gene Levels and Enrichment Analysis

The differential analysis was carried out on the samples between groups. First, the DMS and DMGs were determined by DESeq v1.18.0 [28] on the sequencing depth information on each sample. Then, the basemean values were used to estimate the expression by calculating the multiple difference (fold change). The negative two-distribution test method was used to test the significant difference in the number of reads. Finally, the differential methylation sites were screened based on the multiple difference and the significance difference test results. A *p*-value less than 0.05 was considered significant. The MA, volcanic, and cluster heat map was performed on the differential methylation levels to reveal differences among the samples. Gene Ontology (GO) and Kyoto Encyclopedia of Genes and Genomes (KEGG) enrichment were performed on the genes associated with the DMS and the DMGs at the CG and CWG sites.

GO term analysis was carried on from the GO database (http://geneontology.org/, accessed on 5 March 2021). GO terms with a *p*-value less than 0.05 were considered significantly enriched. The Benjamini-Hochberg multiple testing then corrected all the *p*-values to obtain the FDR. Next, Kyoto Encyclopedia of Genes and Genomes (KEGG: http://www.genome.jp/kegg/, accessed on 5 March 2021) was applied to reveal the pathway enrichment involving the genes with the methylation levels. The significance of the pathway enrichment was derived using the hypergeometric distribution method to obtain the *p*-value < 0.05 in each pathway.

### 4.8. Validation of DMGs and DEGs Associated with DMS by qRT-PCR

We validated the differentially methylated genes (DMGs) and differentially expressed genes (DEGs) associated with differentially methylated sites (DMS) in the leaves as determined by MethylRAD-seq. To this, 23 genes in DMGs and DEGs associated with the spliceosome, phenylpropanoid biosynthesis pathway, carbon metabolism, amino acid biosynthesis, RNA transport, plant hormone signal transduction, and quorum sensing were subjected to quantitative real-time PCR. Total RNAs were isolated from mulberry leaves of various treatment conditions sampled at the 5-day time point after the 14 days drought stress using RNAiso Plus reagent (Takara, Beijing, China) followed by DNase I treatment to remove any genomic DNA contamination according to the manufacture’s protocols. The RNAs were then quantified by computing the absorbance at 260 nm. The RNA was used as the template to synthesize cDNA from 1 µg of total RNA using TRUEscript Reverse Transcription Kit (Aidlab, Beijing, China). Gene-specific qRT-PCR primers were designed by NCBI primer blast regarding the CDS (Table S6) and then synthesized commercially.

The qRT-PCR analysis was conducted with the StepOnePlus Real-Time PCR System (Thermo Fisher Scientific, Waltham, MA, USA). The reaction solution consists of 10 μL SYBR Green I Master Mix (CWBIO, Beijing, China), 1 μmol L^−1^ primers (SANGON BIOTECH, Shanghai, China), and 1 μL each template, making a total volume of 20 μL. The PCR program was as follows: 95 °C for 3 min; 40 cycles of 94 °C for 15 s, 60 °C for 20 s, and 72 °C for 20 s. The resulting fragments were immediately subjected to a melting-curve analysis to verify the amplification of gene-specific PCR products. The melting-curve analysis was completed with the following program: 94 °C for 15 s, followed by a constant increase from 60 to 95 °C at a 2% ramping rate. The mulberry actin3 gene (HQ163775.1) was used as an internal control gene. All samples were analyzed with three biological replicates, each comprising three technical replicates. Relative gene expression levels were calculated according to the 2^−ΔΔCt^ method. Statistical analysis was performed using GraphPad Prism 9 software. Significant difference analysis was performed by Student’s t-test at a significance level of *p* < 0.05.

## 5. Conclusions

In this study, we investigated the DNA methylation status and its effect on gene regulation in mulberry variety Yu-711 under drought stress and control using the leaves sampled at 5 days time point after 14 days drought stress. Our findings reveal that CG methylation status was more prevalent (37.37%) than the CWG methylation (28.81%) in the mulberry genome between drought stress leaves and the control. The drought stress condition induces methylation slightly more than the control treatment. The methylation status occurred in the TSS and TTS more than in the gene body. Again, the methylation status distribution in the functional components of the genome mainly occurred in exon, intergenic, intron, and downstream of the mCG and mCWG sites.

In addition, 170 DMGs and 581 DMS were identified from both CG and CWG methylation sites. GO term functional analysis reveals that the DMGs and DEGs associated with DMS were enriched in three biological process, cellular components, and molecular functions. The KEGG enrichment pathway analysis indicates that these genes are implicated in plant hormone signal transduction, spliceosome, carbon metabolism, RNA transport, biosynthesis of amino acid, and quorum sensing pathways play a key role in drought stress response in mulberry. The qRT-PCR analysis results indicate that the 23 genes involved in the top KEGG enrichment analysis have dynamic gene expression patterns, explaining the complex gene regulation network between DNA methylation and gene expression. The MethylRAD data indicates that 86.96% of the 23 genes selected for the gene expression analysis share a downregulated methylated status, whereas 13.04% shares upregulated methylated status.

Further study on the Functional analysis of these genes involved in the pathways will undoubtedly help us understand the genes’ functions by providing new insight into adaptation mechanisms for mulberry plant response to drought stress conditions in the current global climate change. Thus, this study is significant for understanding the potential role of DNA methylation in the regulation of mulberry plants under drought stress.

## Figures and Tables

**Table 1:** Statistics table for data volume changes.

Sample	Raw_Reads	Norm_Reads	Adapter_Reads	Enzyme_Reads	Range_Reads	Clean_Reads	Percent
EG1	37,614,985	37,614,985	36,965,787	13,237,113	12,235,940	11,702,383	31.11%
EG2	40,658,740	40,658,740	39,893,244	14,298,760	13,247,489	12,694,691	31.22%
CK1	36,045,478	36,045,478	35,360,851	13,247,268	12,171,944	11,649,483	32.32%
CK2	42,583,224	40,657,427	39,894,273	13,596,353	12,403,867	11,906,648	29.29%

**Table 2:** Sample sequencing data quantity vs. ratio.

Sample	Clean	Uniquely	Uniquely	Multiple	Multiple	Total
	Reads	Mapped	Mapped	Mapped	Mapped	Mapped
		Reads	Ratio	Reads	Ratio	Ratio
EG1	11,702,383	1,835,353	15.68%	4,448,071	38.01%	53.69%
EG2	12,694,691	1,992,606	15.70%	4,841,099	38.13%	53.83%
CK1	11,649483	1,689,487	14.50%	4,341,254	37.27%	51.77%
CK2	11,906,648	1,700,091	14.28%	4,425,700	37.17%	51.45%

**Table 3:** Statistics of coverage depth of methylation sites.

Sample	CG_Site_Num	CG_Site_Depth	CWG_Site_Num	CWG_Site_Depth
EG1	34,719 (9.37%)	35.8	14,150 (7.25%)	24.87
EG2	35,208 (9.50%)	38.18	14,624 (7.49%)	26.54
CK1	33,941 (9.16%)	33.02	13,794 (7.07%)	27.19
CK2	34,596 (9.34%)	33.02	13,693 (7.02%)	26.08

## Data Availability

The original data sets described in the study are included in the article/Supplementary Material. The original raw datasets presented in this study can be found in the online repository: Sequence Read Archive (SRA) (https://www.ncbi.nlm.nih.gov/sra/ available online since 30 December 2021) of NCBI (accession number: PRJNA771759). Further inquiries can be addressed to the corresponding author.

## Supplementary Materials

The following are available online at https://www.mdpi.com/article/10.3390/plants11020190/s1, Supplementary Figures (a PDF file containing Figures S1–S24 with captions and legends); Supplementary Tables: Table S1: CG and CWG methylation sites (EG_CK) (an Excel workbook), Table S2: Distribution of mCG and mCWG Sites in the different functional components of the genome (EG_CK) (an Excel workbook), Table S3: Differential methylated site (DMS) and gene (DMGs) and GO analysis (EG_CK) (an Excel workbook), Table S4: Differential methylated Site (DMS) and gene (DMGs) and KEGG analysis (EG_CK) (an Excel workbook), Table S5: Differential methylated genes at the promoter level (EG_CK) (an Excel workbook), Table S6: Gene name and primers sequences used for qRT-PCR gene validation and MethylRAD primer and adaptor sequences (an Excel workbook).
