# Supplementary material for: DNA Methylation Changes and Its Associated Genes in Mulberry (Morus alba L.) Yu-711 Response to Drought Stress Using MethylRAD Sequencing
Source: Plants (Basel). 2022 Jan 12;11(2):190. doi: 10.3390/plants11020190 (PMC8780187; doi:10.3390/plants11020190)
Supplement: Supplementary file 1 [file plants-11-00190-s001.zip › Additional file.pdf]

DNA methylation changes and its associated genes in mulberry (*Morus alba* L.) Yu-711 response to drought stress using MethylRAD sequencing

CK1

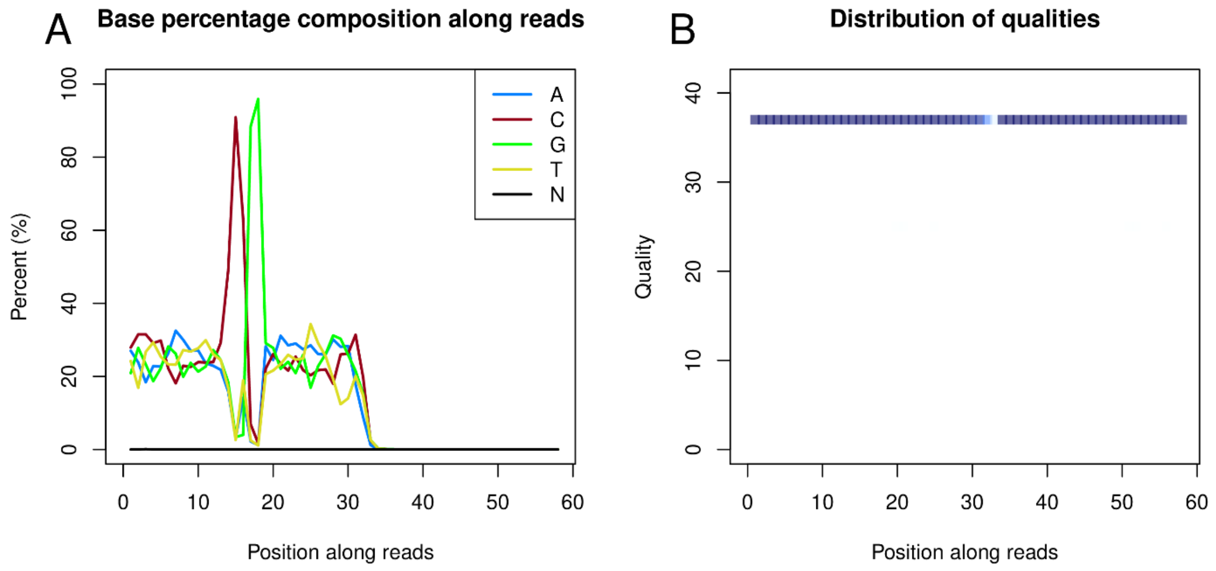

CK2

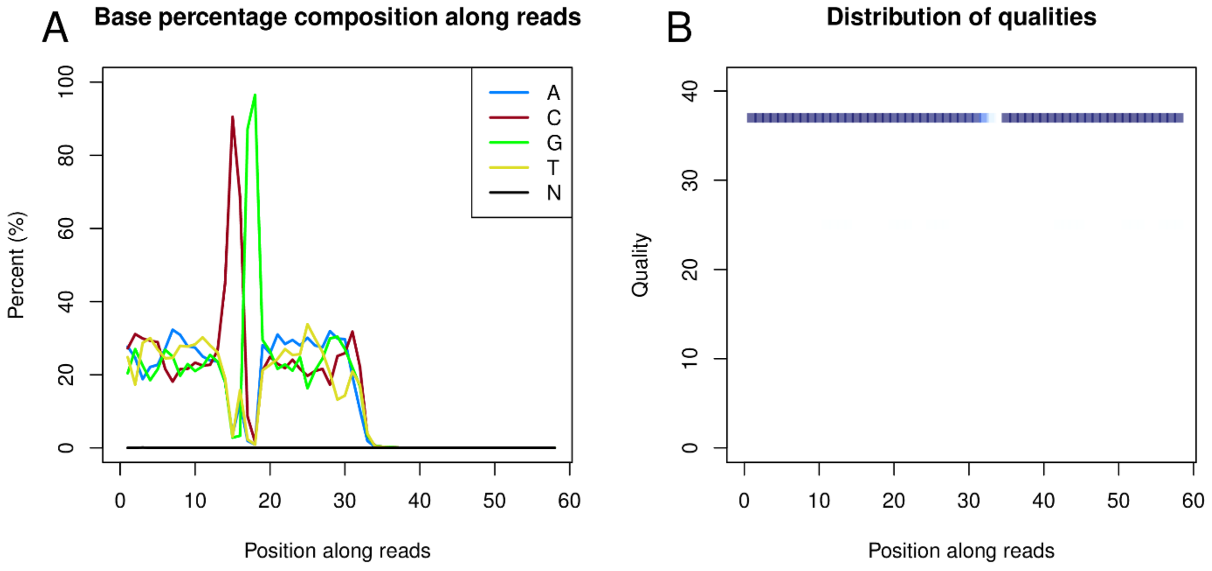

EG1

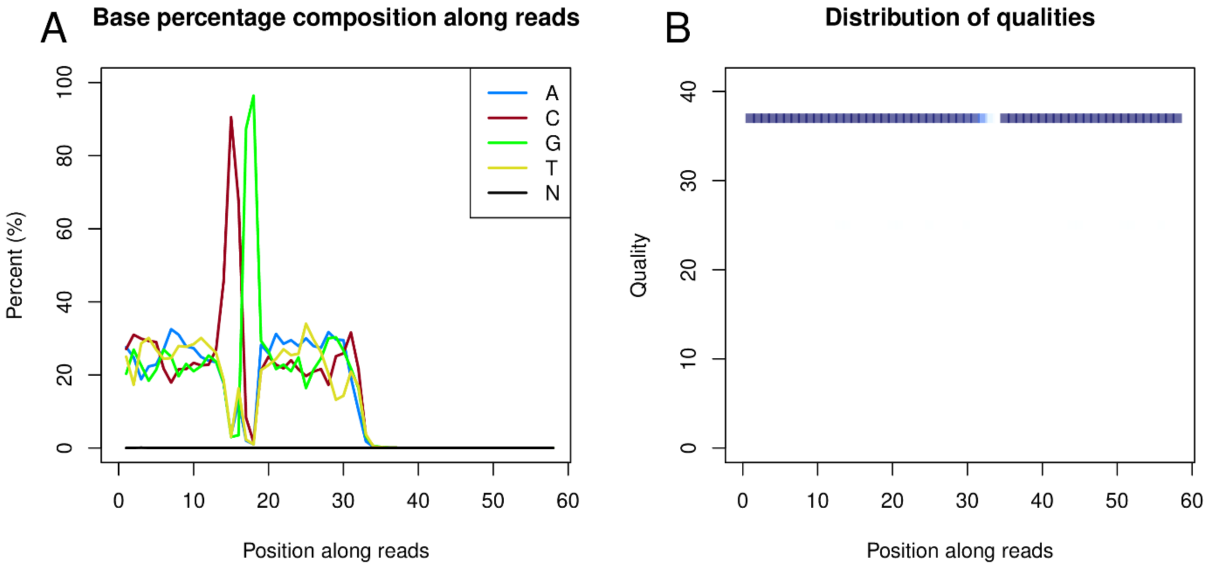

EG2

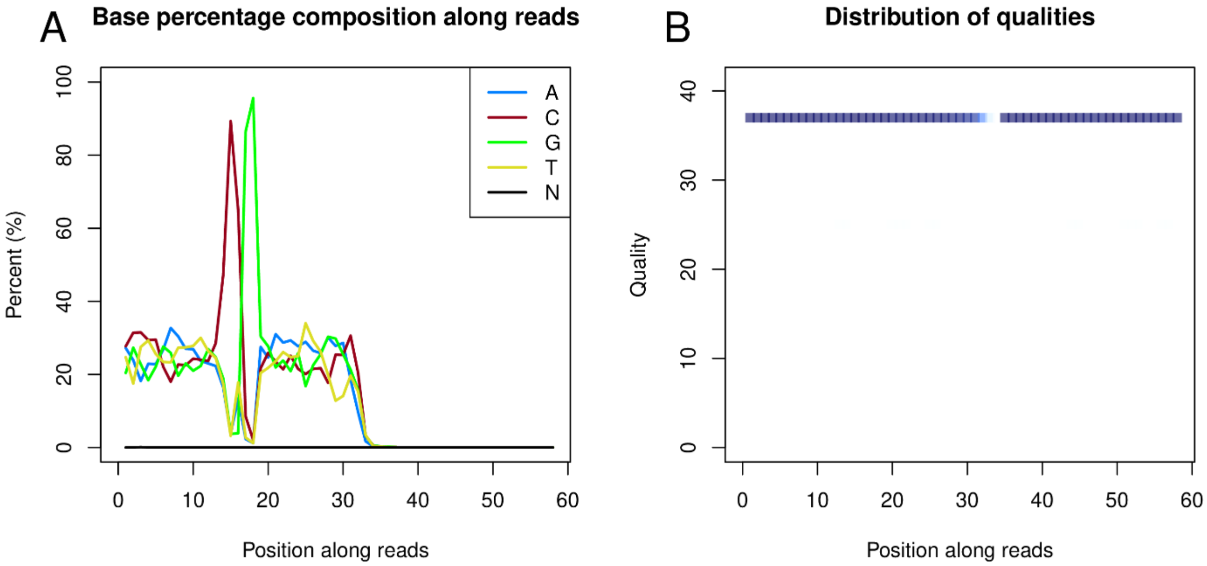

Figure S1. Base distribution map and base mass map of clean reads. Panel A: The horizontal coordinates denote the base position of reads. The ordinates are the proportion of the bases, and the assorted colors represent different bases. The bases that are not identified in the sequencing are labeled N. Panel B: The horizontal coordinates of the base position of reads, and the ordinates are the mass values of the base corresponding to the position.

A

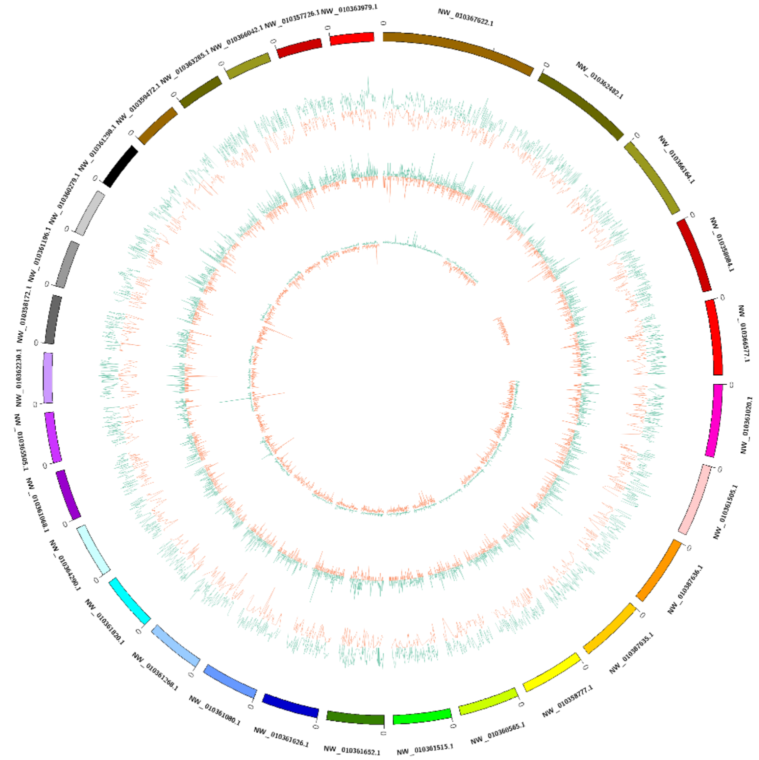

B

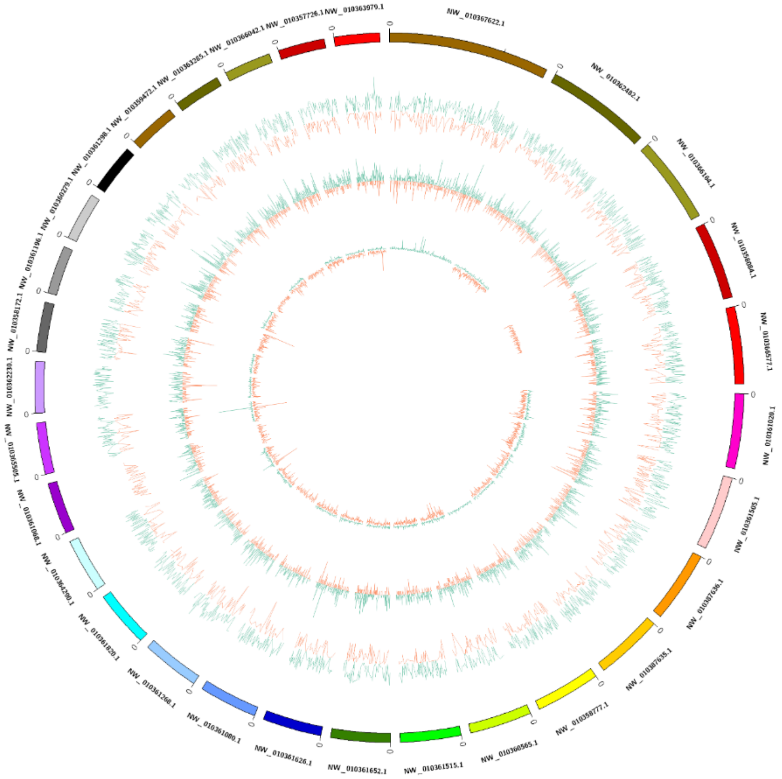

C

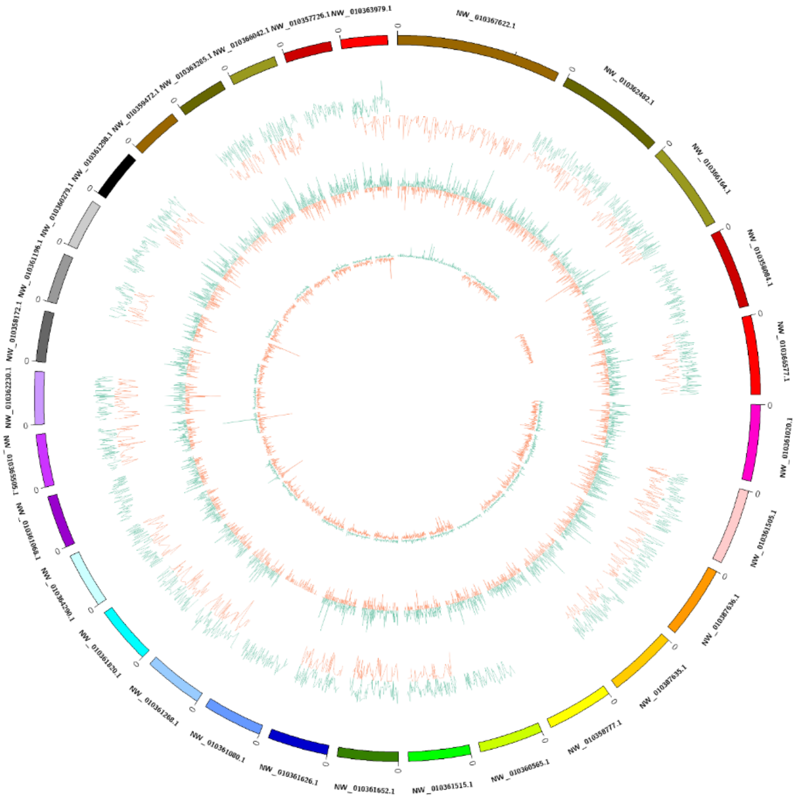

D

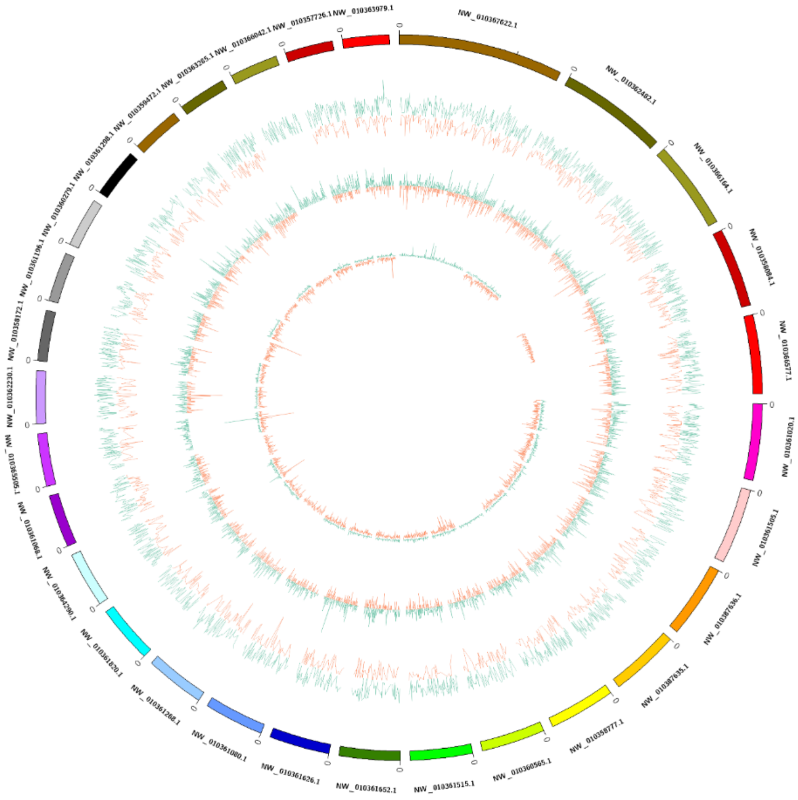

Figure S2. Distribution of methylation sites on the genome. (**A**) distribution in CK1; (**B**) distribution in CK1; (**C**) distribution in EG1; (**D**) distribution in EG2. From outside to inside the windows are chromosome coordinates, reads depth (the sum of the depths of the sites in the window). The number of methylation sites in each window (the actual number of sites) contains genomic electron enzymes and the number of cut sites (theoretical number of sites). Different colors represent different types of methylation sites: green indicates CG sites (the higher the value, the more the broken line extends outward), and the yellow indicates the CWG site, where W=A or T (the higher the value, the more inwardly); each value is taken as log10 for the picture display.

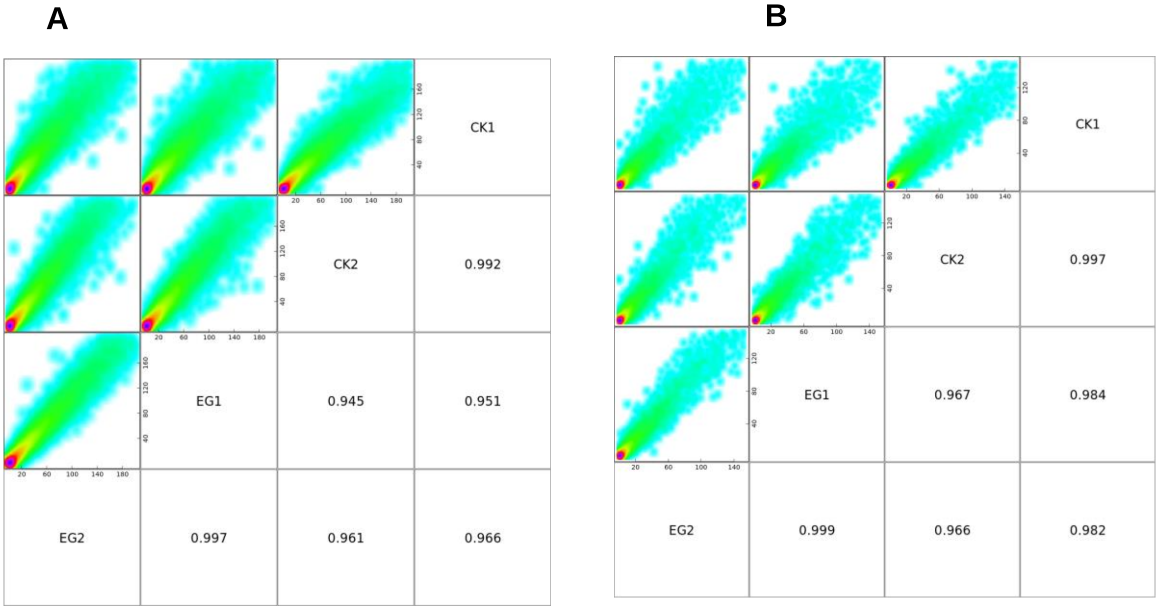

Figure S3. Correlation coefficient diagram between samples. (**A**) CG Pearson's correlation; (**B**) CWG Pearson's correlation. The upper left triangle in the figure is the scatter plot of the methylation levels of the two samples. The lower right triangle area is the corresponding Pearson's correlation coefficient; the diagonal line is the sample name.

A

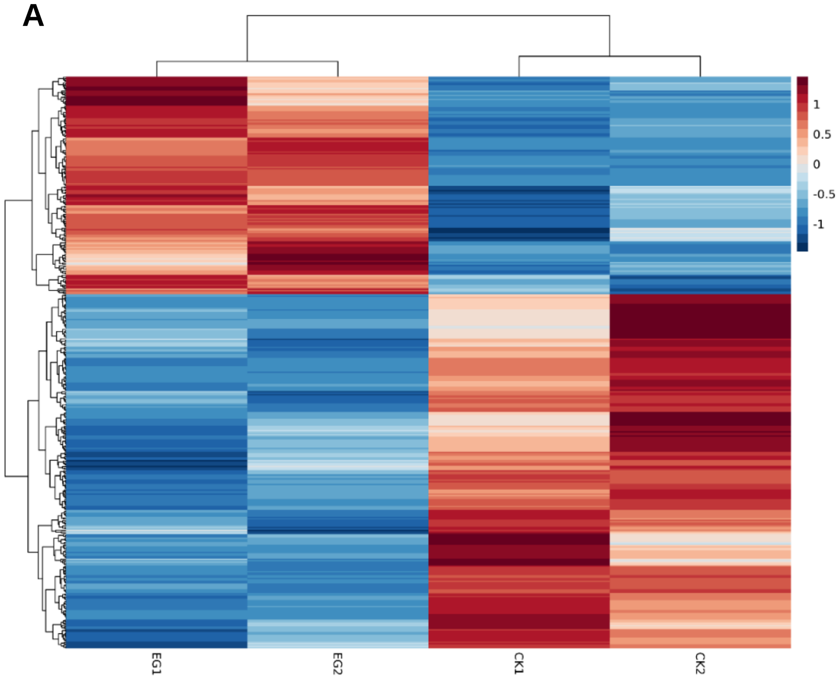

B

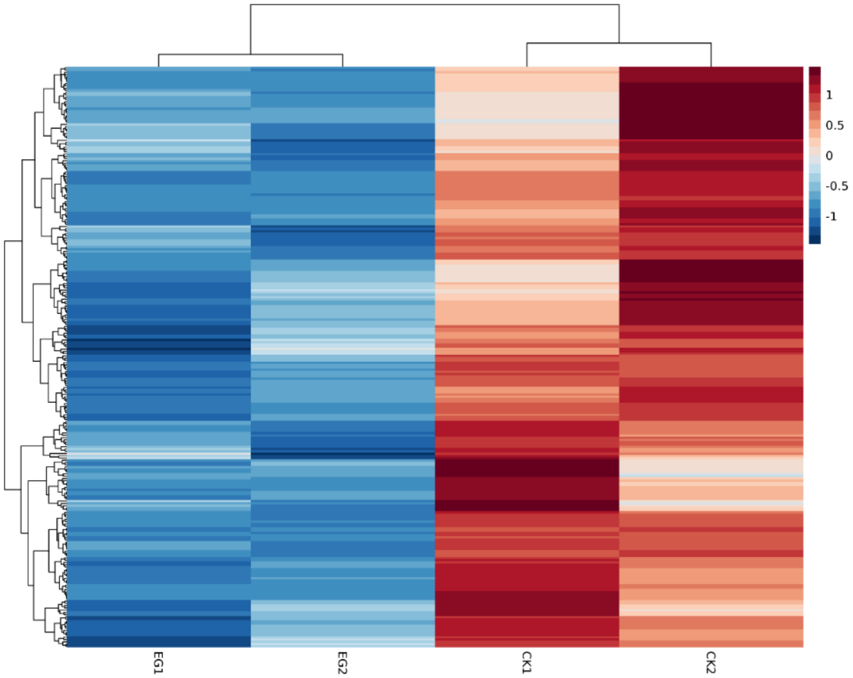

C

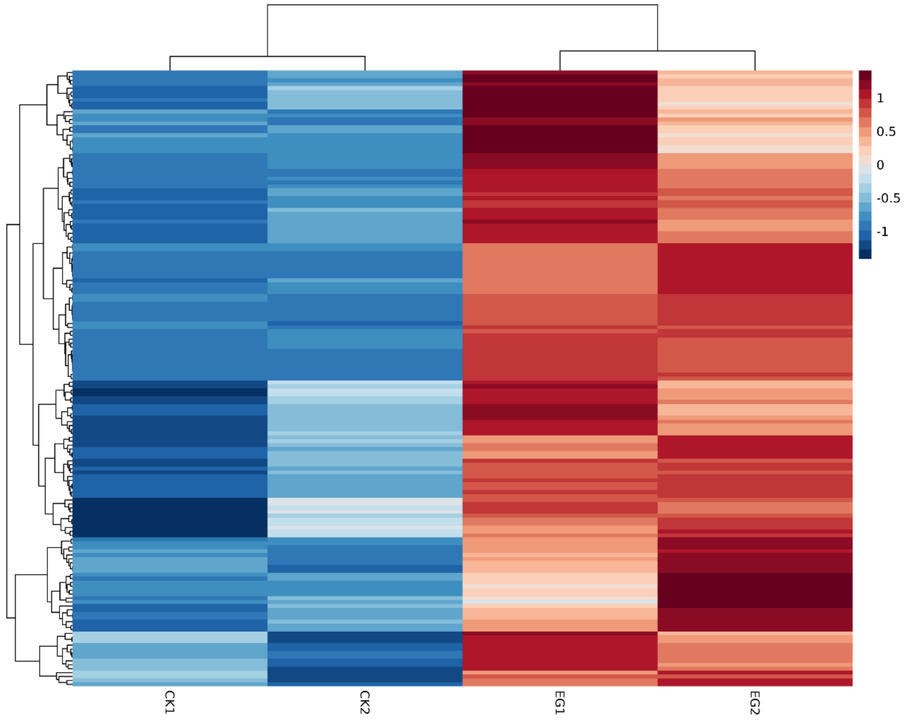

D

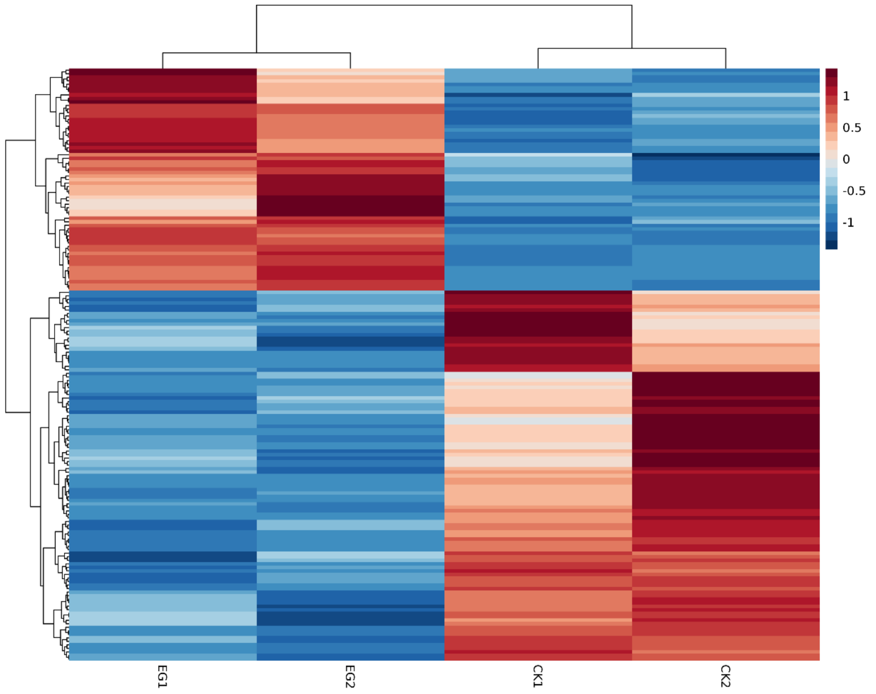

E

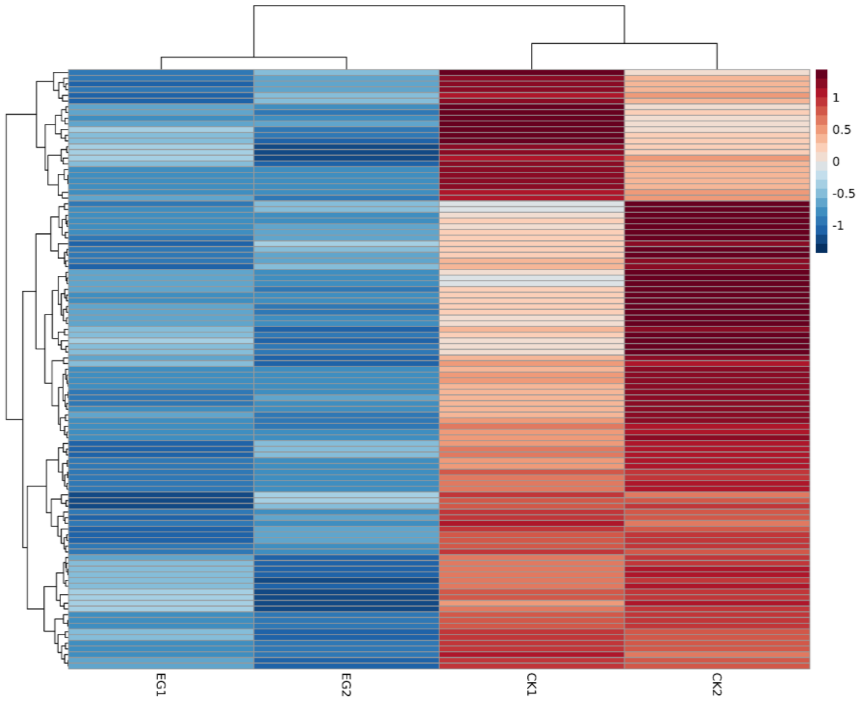

F

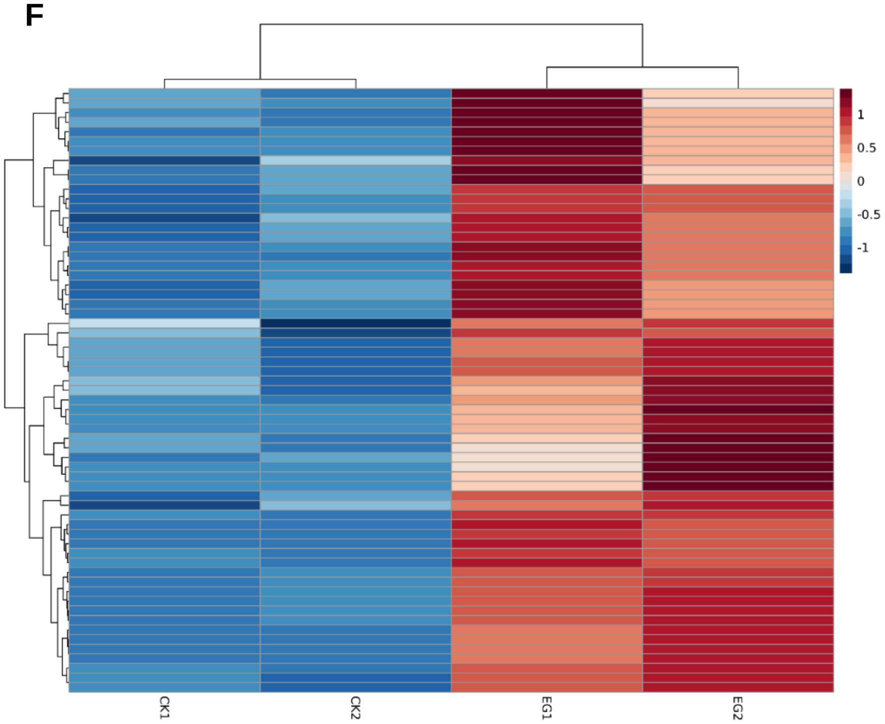

Figure S4. Clustering heat map of differential methylation sites (DMS)between groups. (**A**) CG DMS heat map in EG vs CK; (**B**) CG down-DMS heat map in EG vs CK; (**C**) CG upregulated DMS heat map in EG vs CK.; (**D**) CWG DMS heat map in EG

vs CK; (**E**) CWG down-regulated methylated site heat map in EG vs CK; (**F**) CWG upregulated DMS heat map in EG vs CK. The abscissa is the sample, and the ordinate is the location. The data is standardized by row. From **A-C** are the sites where the expression of CG sites are different, and from d-f are the sites where CWG sites are different. The red color is the site where the expression is upregulated, and the blue color is the site where the expression is down-regulated**.**

A

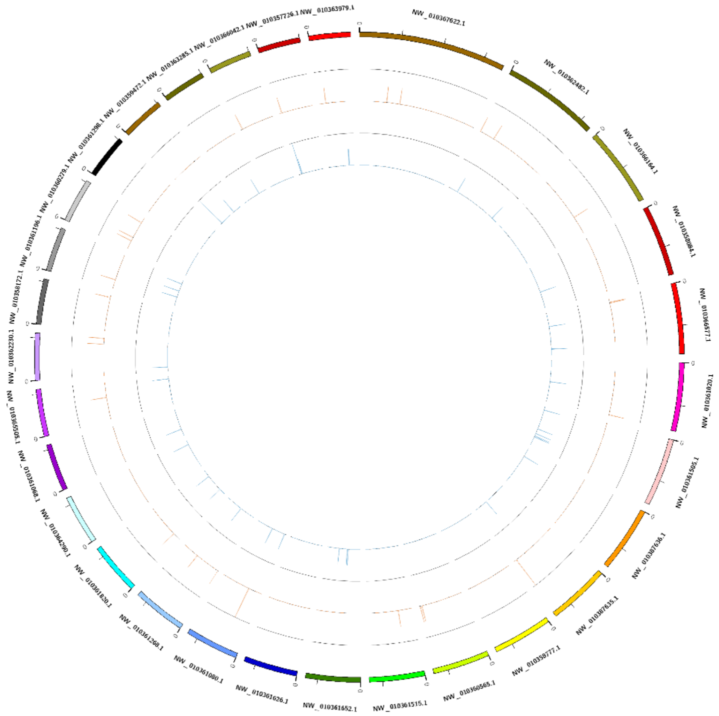

B

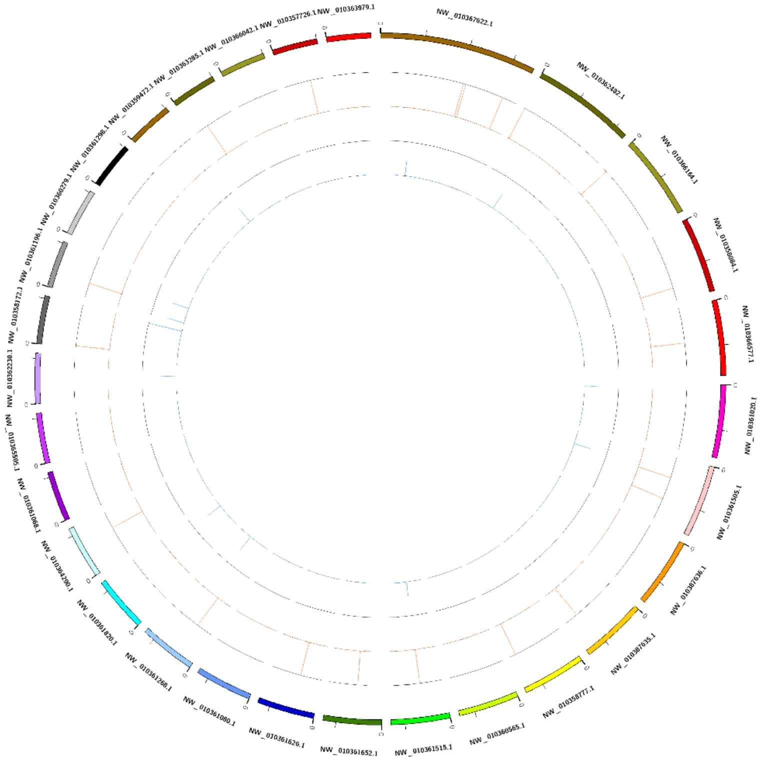

Figure S5. Distribution of DMS at the CG and CWG level in the chromosome. (**A**) the distribution on the chromosome at the CG site; (**B**) the chromosome at the CWG site

A

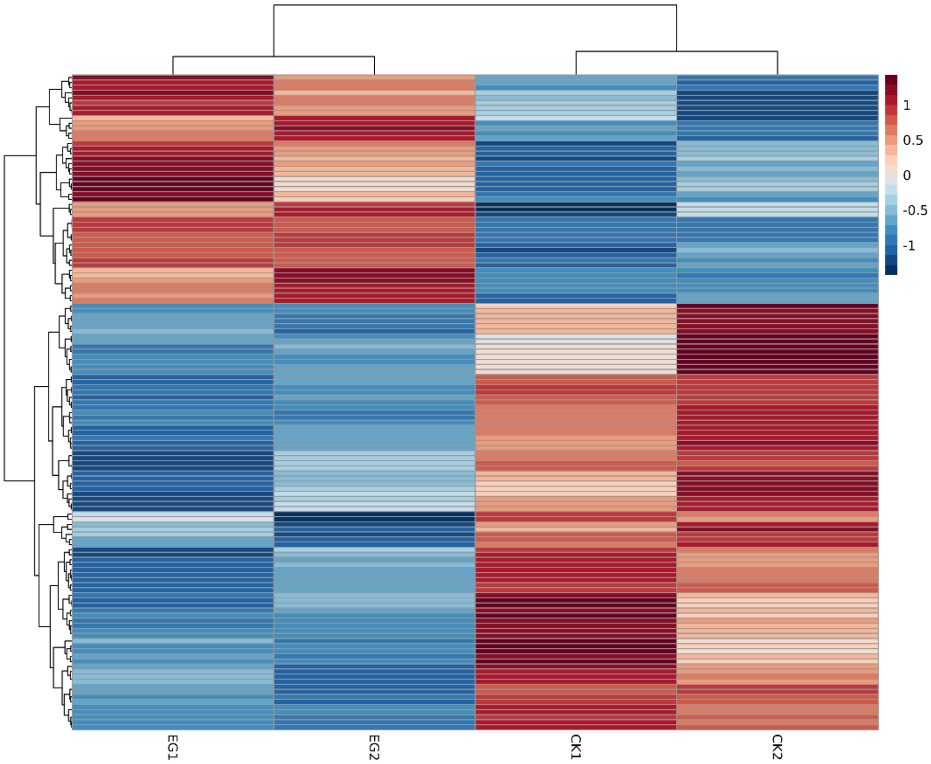

B

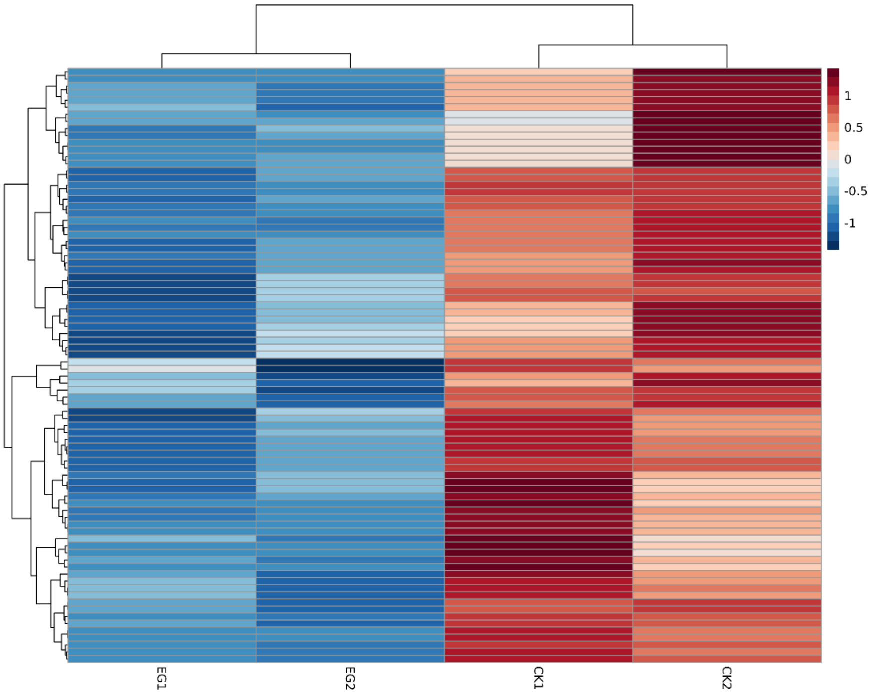

C

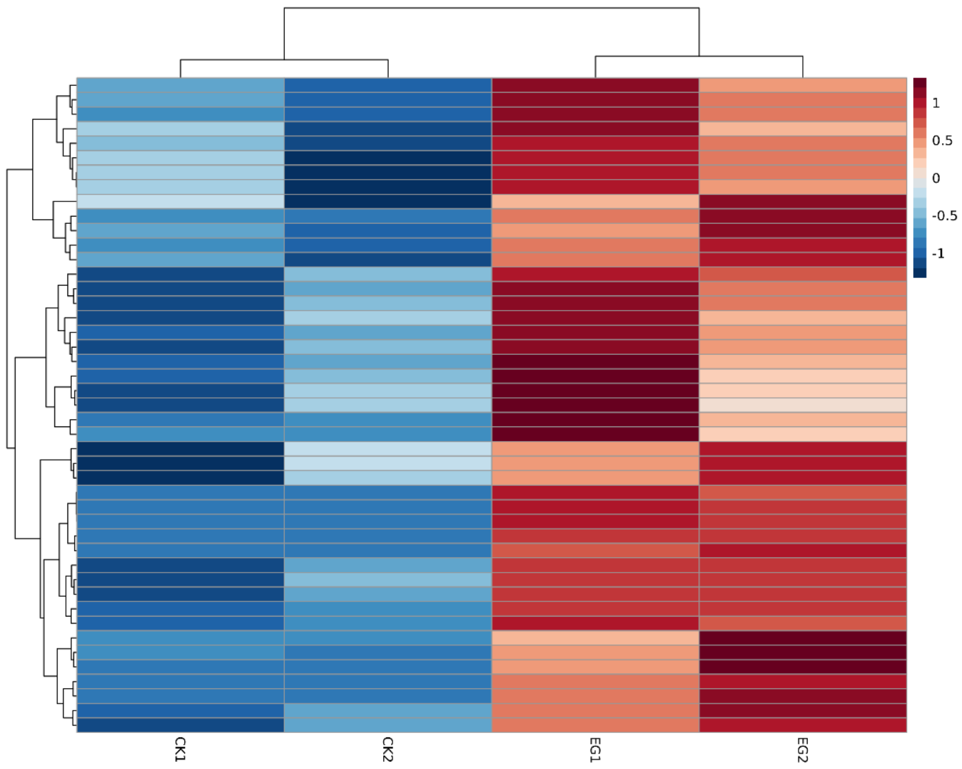

D

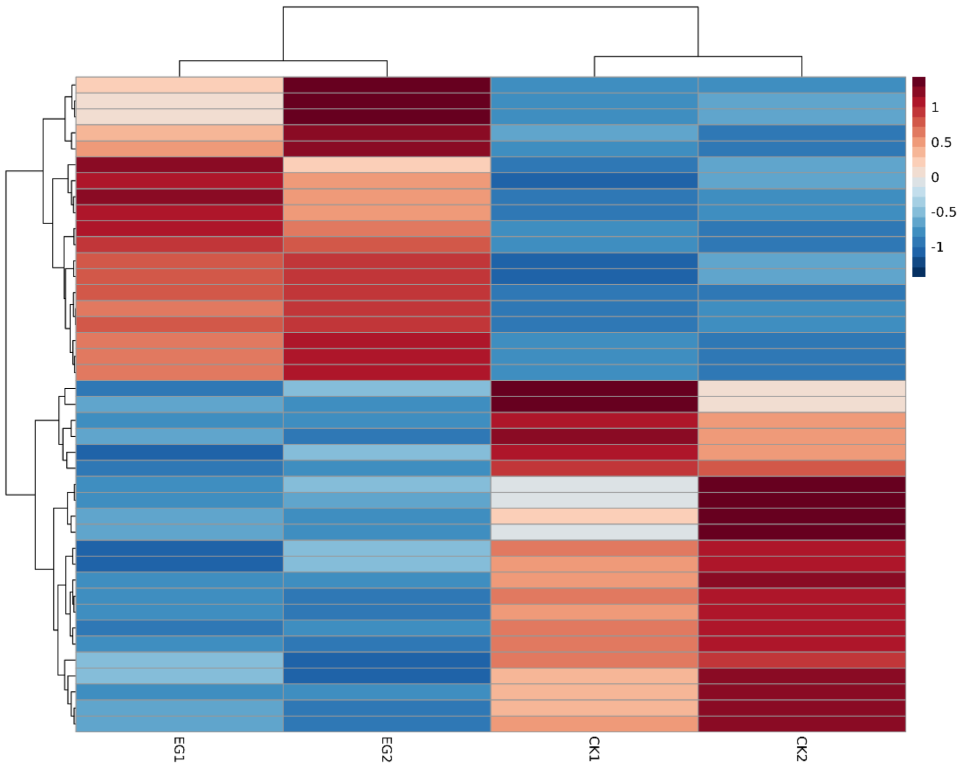

E

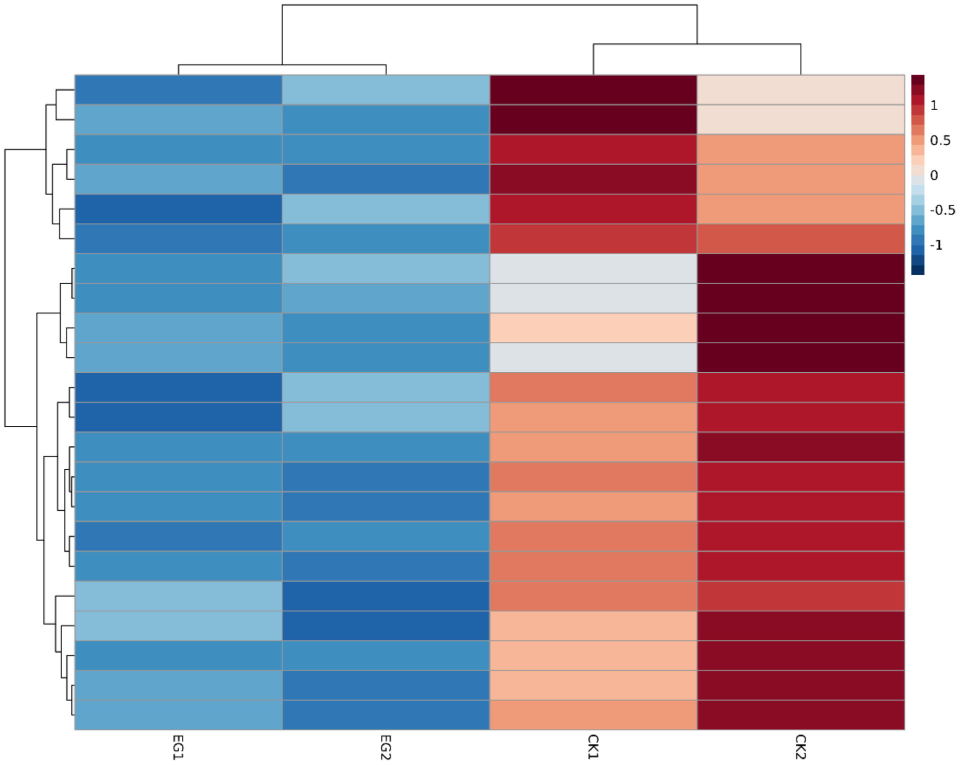

F

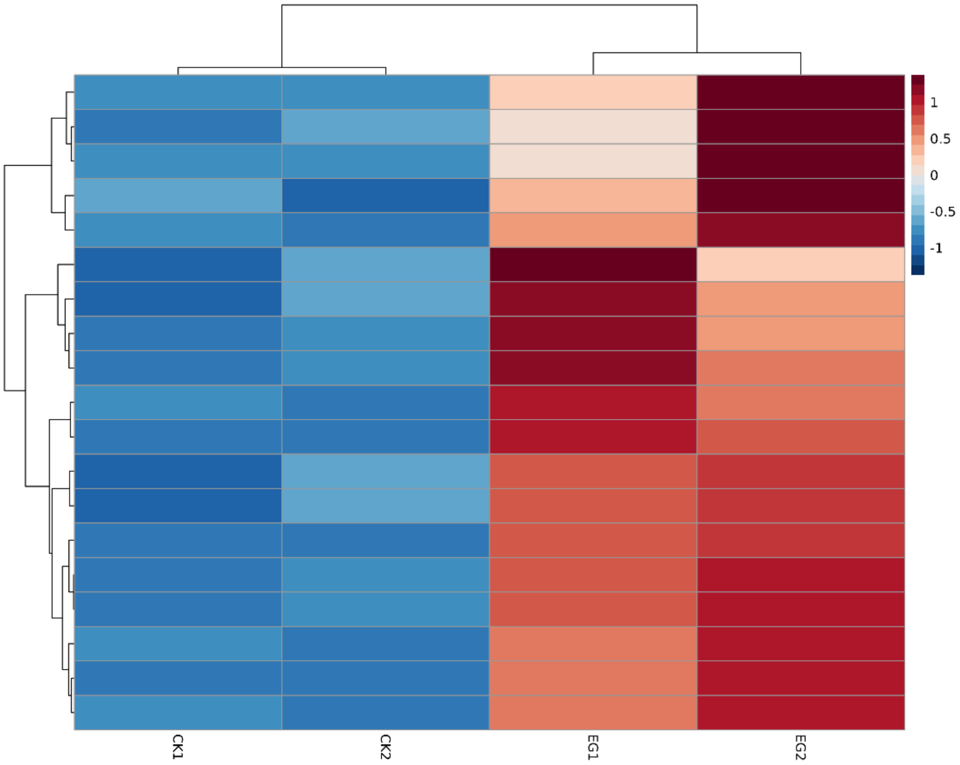

Figure S6. Clustering heat map of differential methylation genes (DMGs) between EG-vs-CK. (**A**) CG DMGs heat map in EG vs CK; (**B**) CG down-regulated DMGs heat map in EG vs CK; (**C**) CG upregulated methylated site heat map in EG vs CK; (**D**) CWG DMGs heat map in EG vs CK; (**E**) CWG downregulated DMGs heat map in EG vs CK. (**F**) CWG upregulated methylated site heat map in EG vs CK. The abscissa is the sample, and the ordinate is the location. The data is standardized by row. From **A-C** are the sites where the expression of CG sites are different, and from **D-E** are the sites where CWG sites are different. The red color is the sites where the expression is upregulated, and the blue color is the sites where the expression is downregulated

A

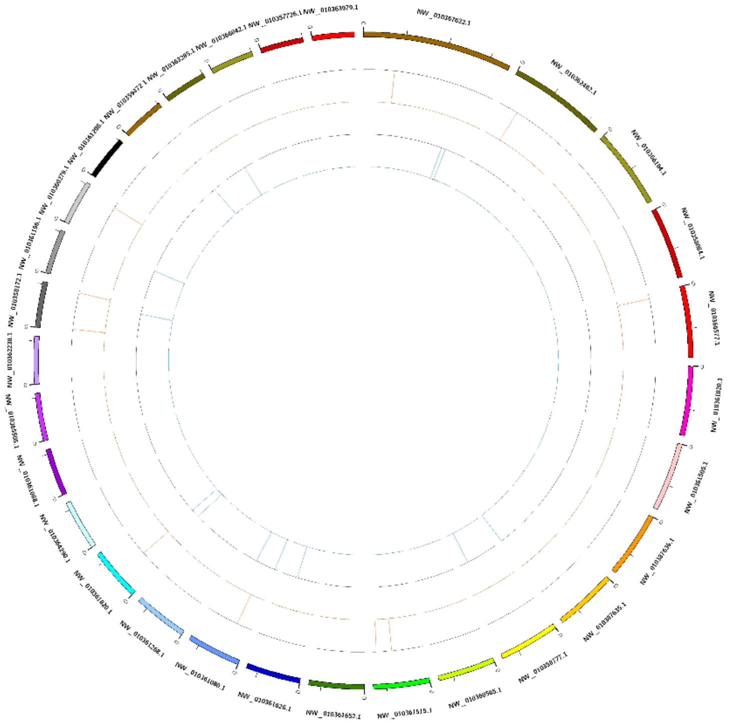

B

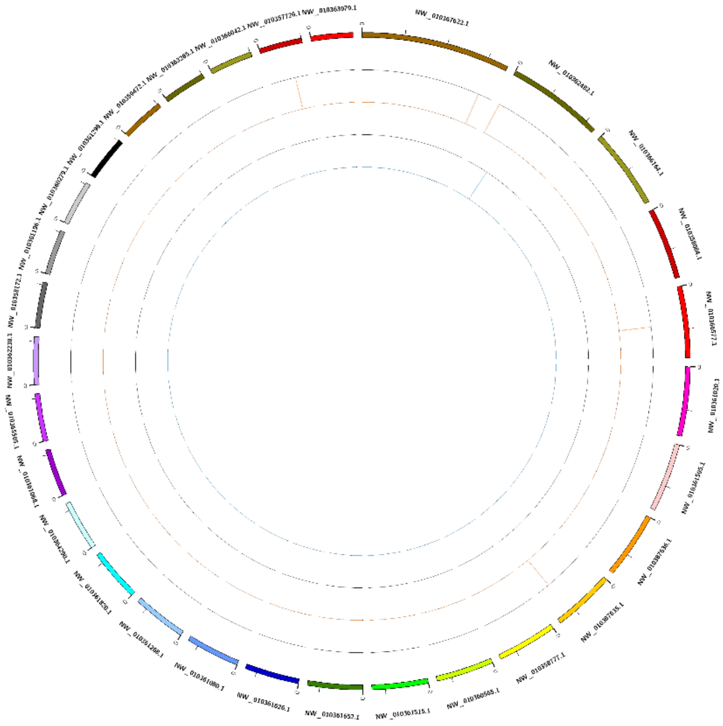

Figure S7. Distribution of DMGs at the CG and CWG level in the chromosome. (**A**) the distribution on the chromosome at the CG site; (**B**) the chromosome at the CWG site

A

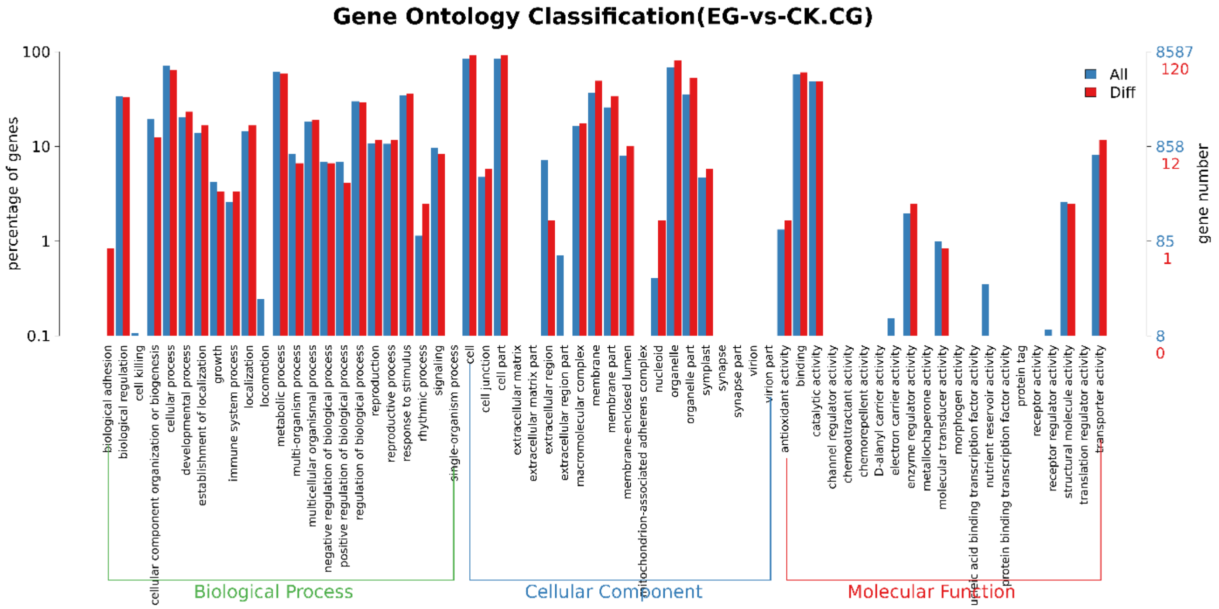

B

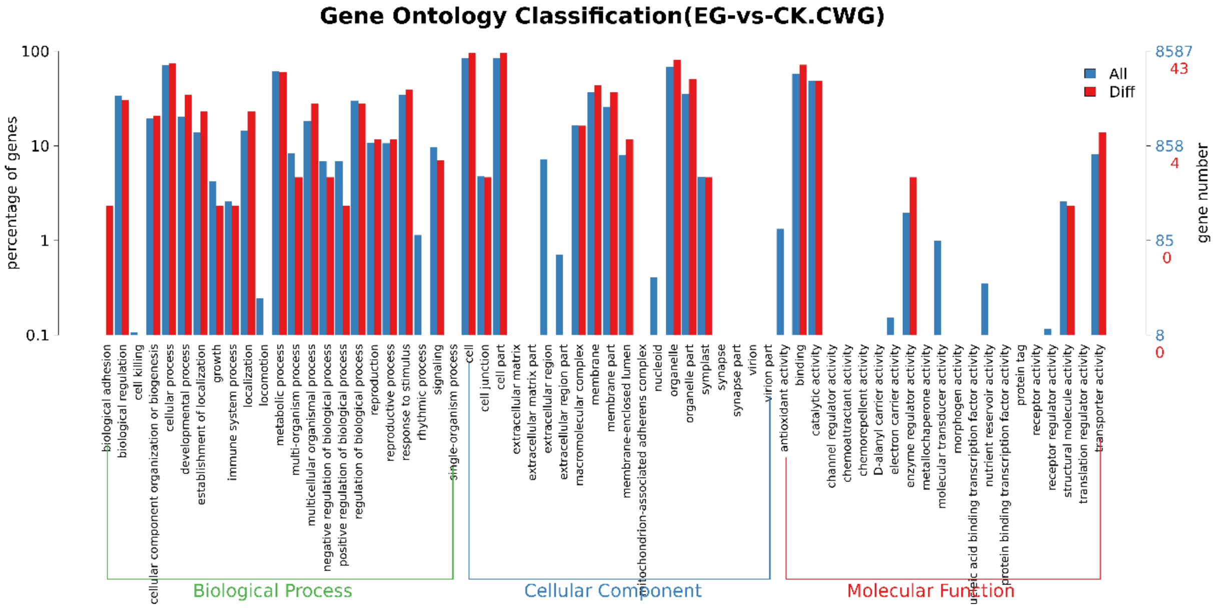

Figure S8. A comparison of the distribution of the list of enriched genes (DEGs) and all genes at the GO level where the differential methylation sites are located. (**A**) GO classification at the CG methylation site. (**B**) GO classification at the CWG methylation site. Columns in blue indicate GO level entries for all gene enrichment. Columns in red are the GO level entries for difference expression gene enrichment, the horizontal axis for level horizontal entry names, and the vertical axis for corresponding difference expression genes and their percentage.

A

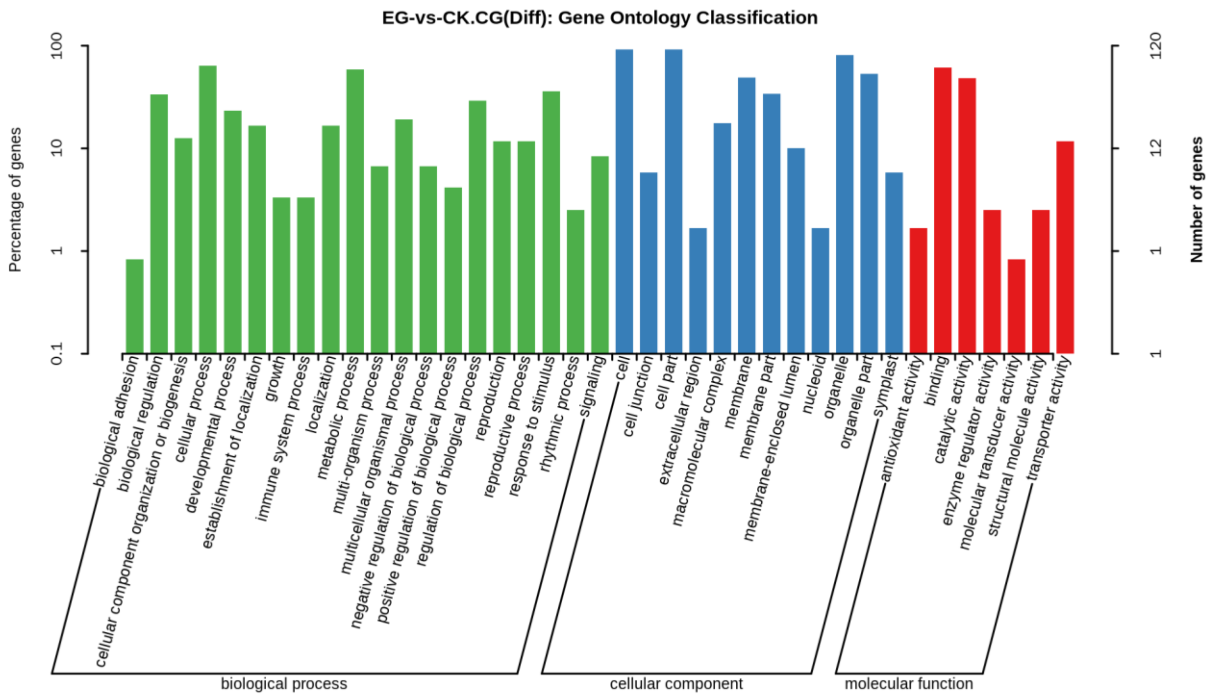

B

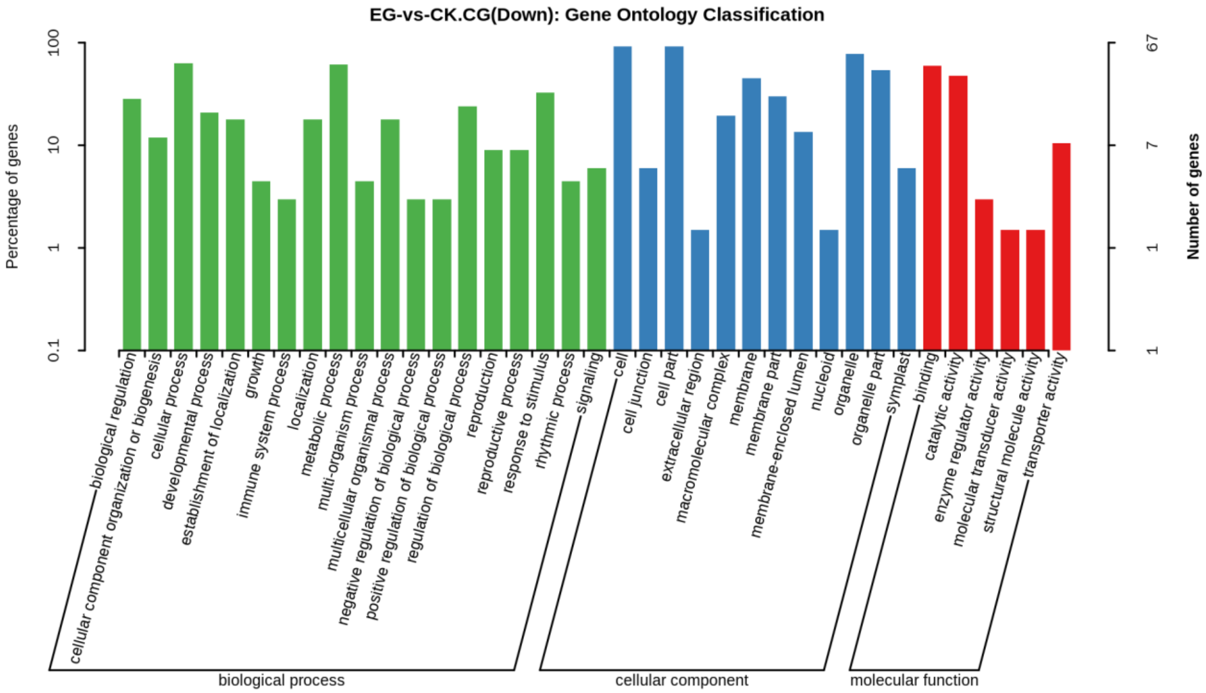

C

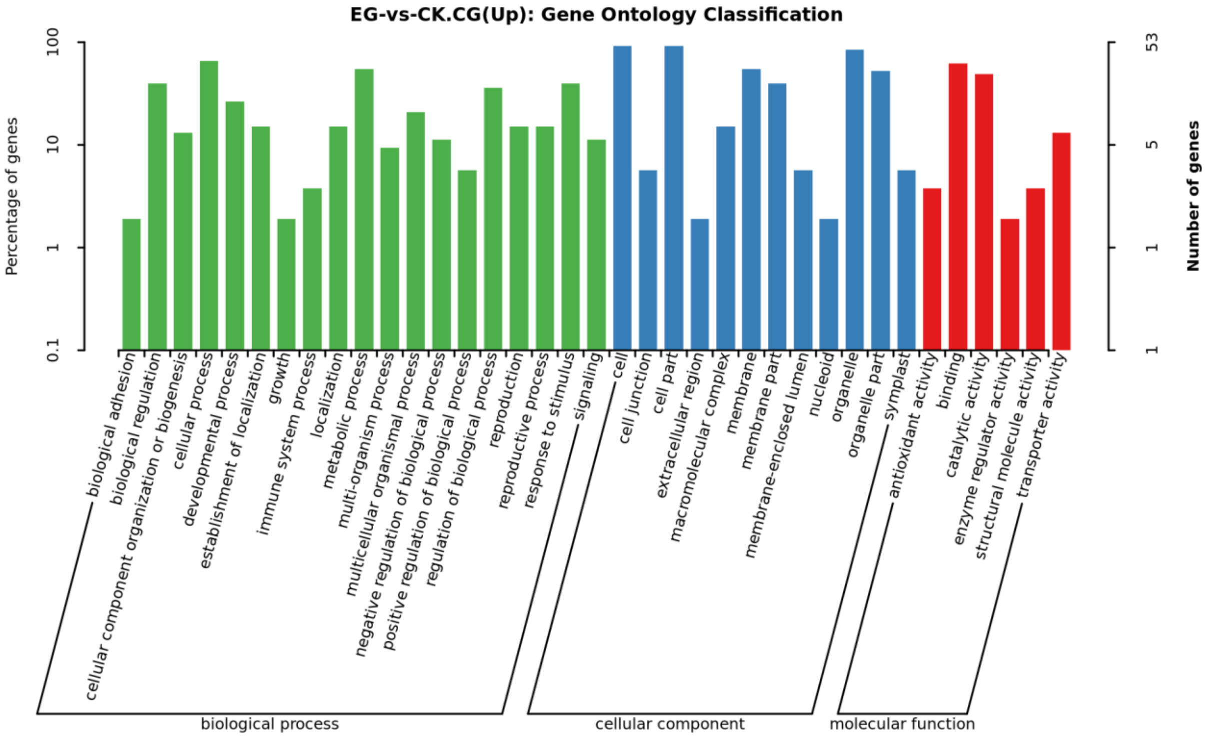

Figure S9. Comparison of the GO classification of the DEGs where the CG differential methylation sites are located. (**A**) GO classification of the DEGs at the CG site; (**B**,**C**) The DEGs in the GO terms at the CG site that were down and upregulated

A

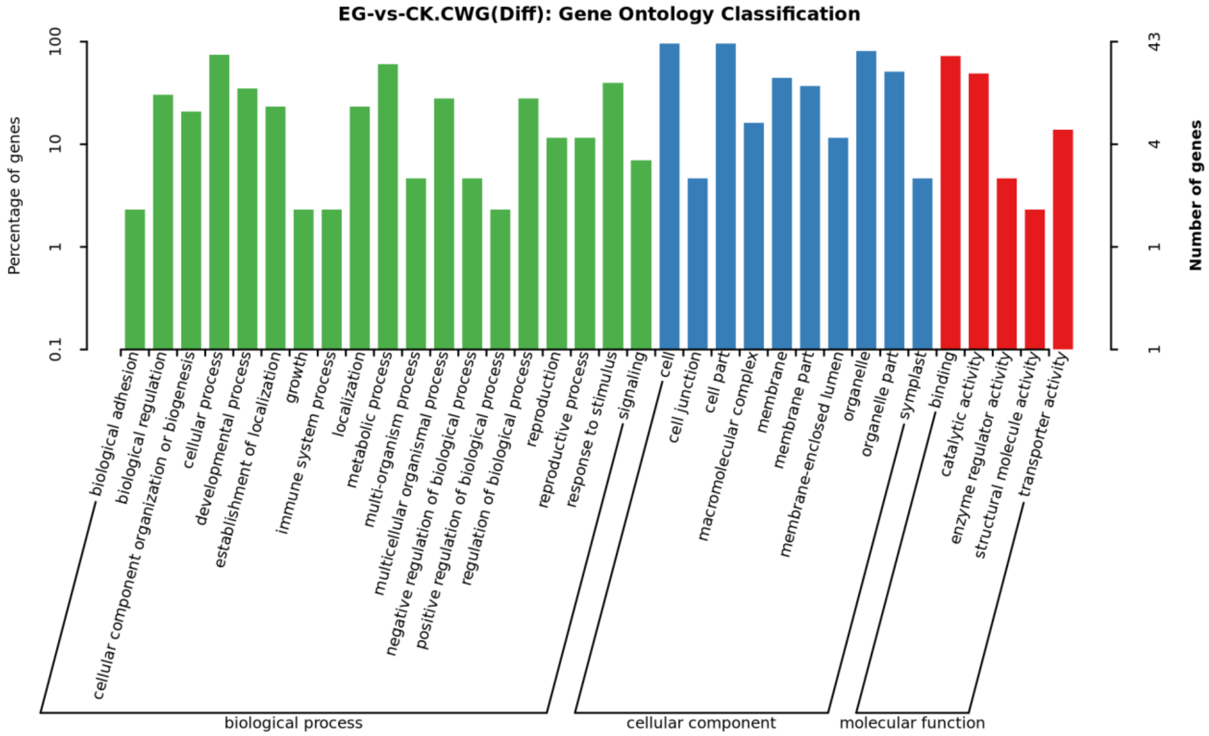

EG-vs-CK.CWG(Diff): Gene Ontology Classification
Percentage of genes
100
10
1
0.1
Number of genes
43
4
1
1
biological adhesion
biological regulation
cellular component organization or biogenesis
cellular process
developmental process
establishment of localization
growth
immune system process
localization
metabolic process
multi-organism process
multicellular organismal process
negative regulation of biological process
positive regulation of biological process
regulation of biological process
reproduction
reproductive process
response to stimulus
signaling
cell
cell junction
cell part
macromolecular complex
membrane
membrane part
membrane-enclosed lumen
organelle
organelle part
symplast
binding
catalytic activity
enzyme regulator activity
structural molecule activity
transporter activity
biological process
cellular component
molecular function

B

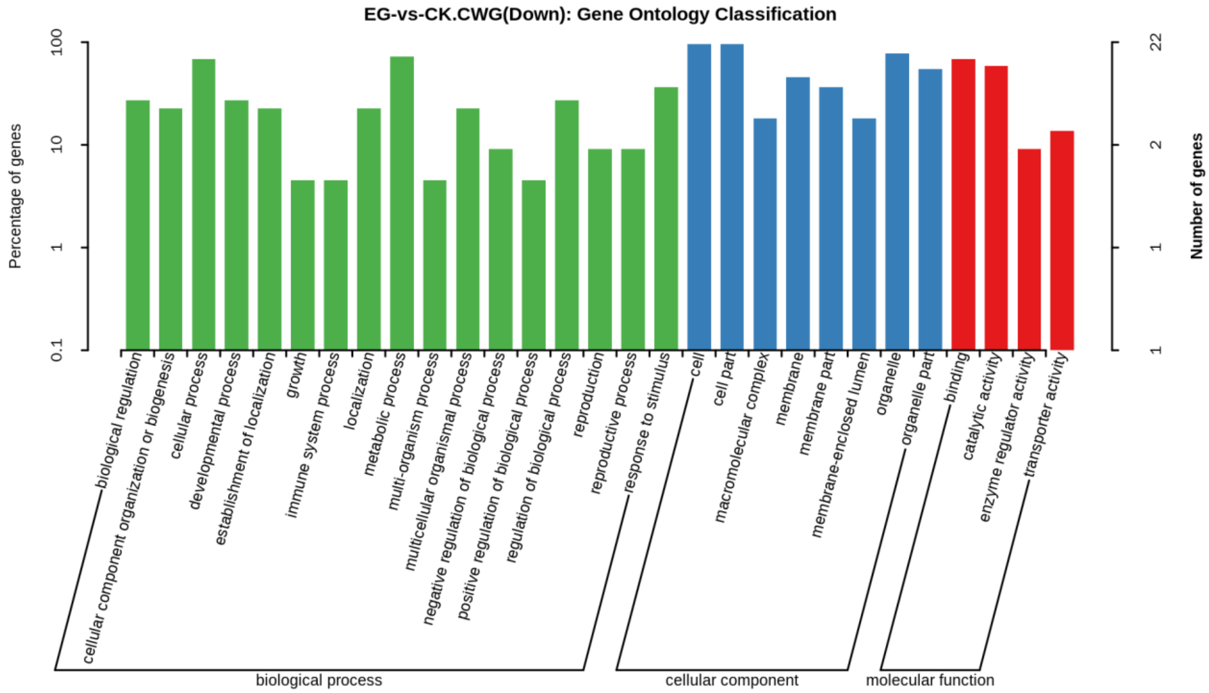

EG-vs-CK.CWG(Down): Gene Ontology Classification
Percentage of genes
100
10
1
0.1
Number of genes
22
2
1
1
biological regulation
cellular component organization or biogenesis
cellular process
developmental process
establishment of localization
growth
immune system process
localization
metabolic process
multi-organism process
multicellular organismal process
negative regulation of biological process
positive regulation of biological process
regulation of biological process
reproduction
reproductive process
response to stimulus
cell
cell part
macromolecular complex
membrane
membrane part
membrane-enclosed lumen
organelle
organelle part
binding
catalytic activity
enzyme regulator activity
transporter activity
biological process
cellular component
molecular function

C

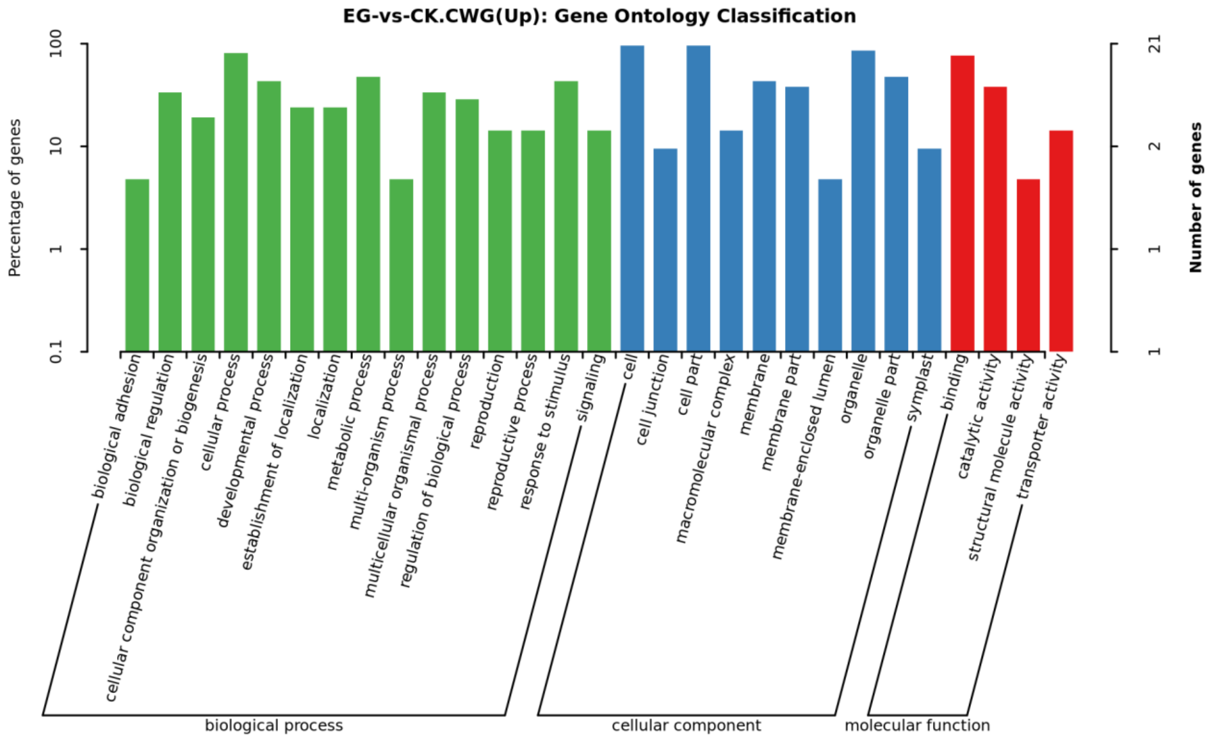

Figure S10. Comparison of the GO classification of the DEGs where the CWG differential methylation sites are located. (**A**) GO classification of the DEGs at the CWG site. (**B**,**C**) The DEGs in the GO terms at the CG site that were down and upregulated

A

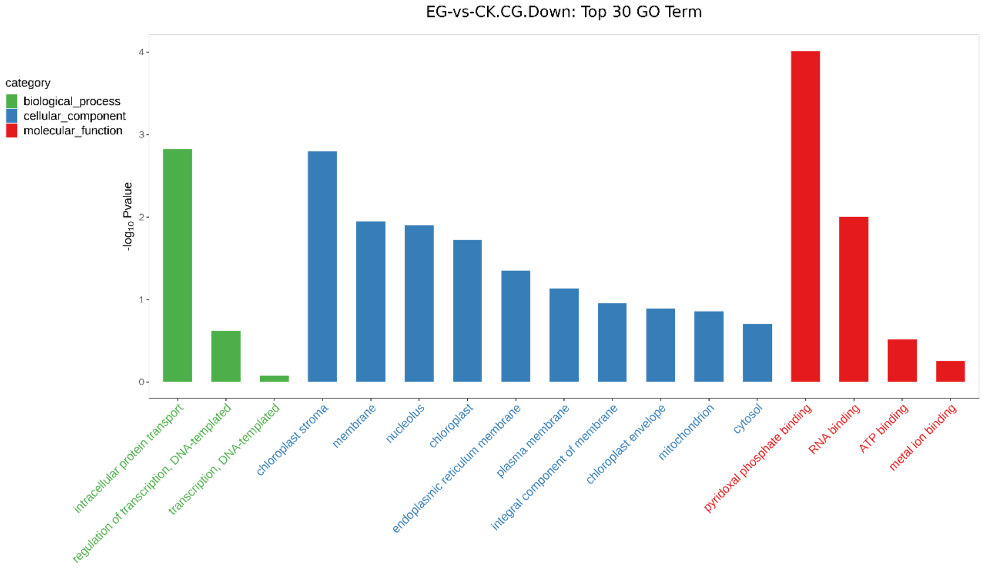

B

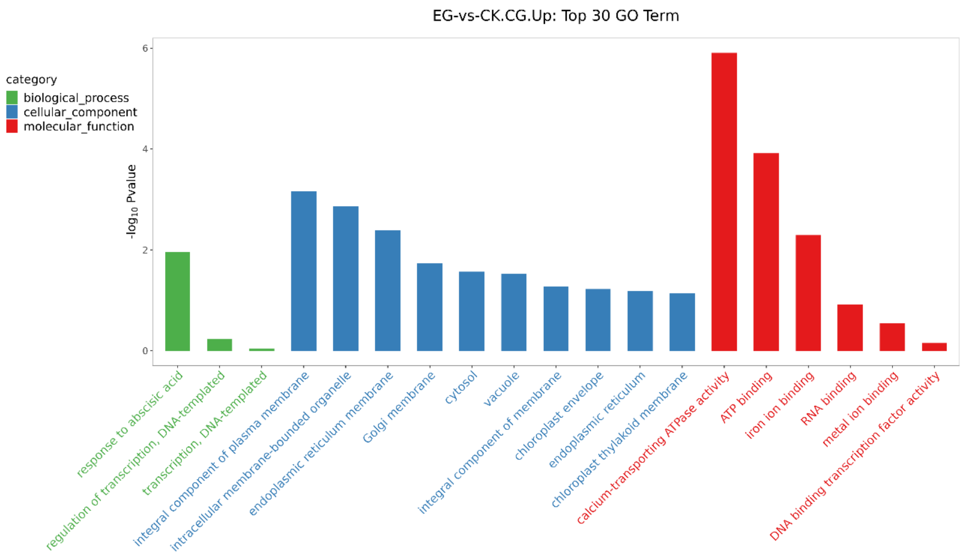
EG-vs-CK.CG.Up: Top 30 GO Term
category
biological_process
cellular_component
molecular_function
-log10 Pvalue
0
2
4
6
response to abscisic acid
regulation of transcription, DNA-templated
transcription, DNA-templated
integral component of plasma membrane
intracellular membrane-bounded organelle
endoplasmic reticulum membrane
Golgi membrane
cytosol
vacuole
integral component of membrane
chloroplast envelope
endoplasmic reticulum
chloroplast thylakoid membrane
calcium-transporting ATPase activity
ATP binding
iron ion binding
RNA binding
metal ion binding
DNA binding transcription factor activity

C

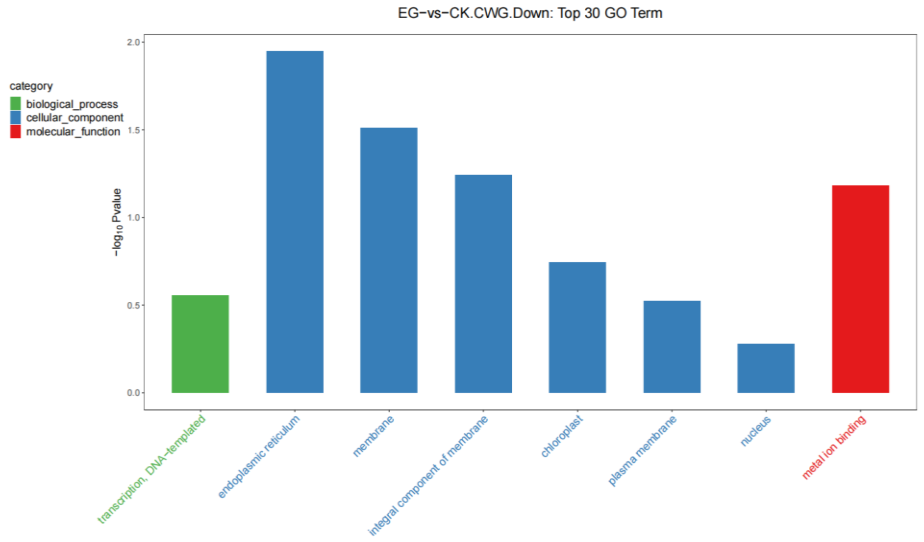
EG-vs-CK.CWG.Down: Top 30 GO Term
category
biological_process
cellular_component
molecular_function
-log10 Pvalue
0.0
0.5
1.0
1.5
2.0
transcription, DNA-templated
endoplasmic reticulum
membrane
integral component of membrane
chloroplast
plasma membrane
nucleus
metal ion binding

D

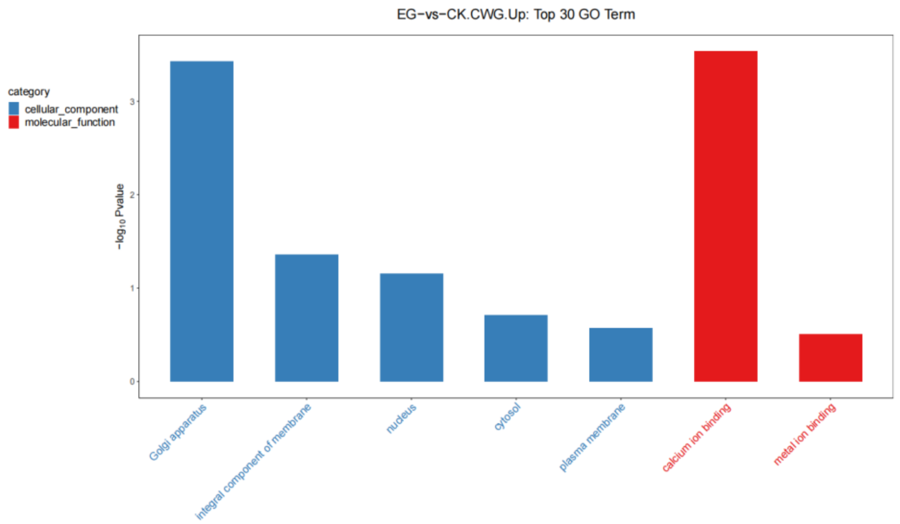
EG-vs-CK.CWG.Up: Top 30 GO Term
category
cellular_component
molecular_function
-log10 Pvalue
0
1
2
3
Golgi apparatus
integral component of membrane
nucleus
cytosol
plasma membrane
calcium ion binding
metal ion binding

Figure S11. Bar chart of the top 30 GO functions of the gene where CG and CWG up and downregulated differential methylation sites are located. (**A**) the top 30 down regulated differential GO terms at CG sites; (**B**) the top 30 upregulated differential GO terms at CG sites; (**C**) the top 30 down regulated differential GO terms at CWG sites; (**D**) the top 30 upregulated differential GO terms at CWG sites. The X coordinates in the figure are the name of the GO entry, and the Y coordinates are -log10Pvalue.

A

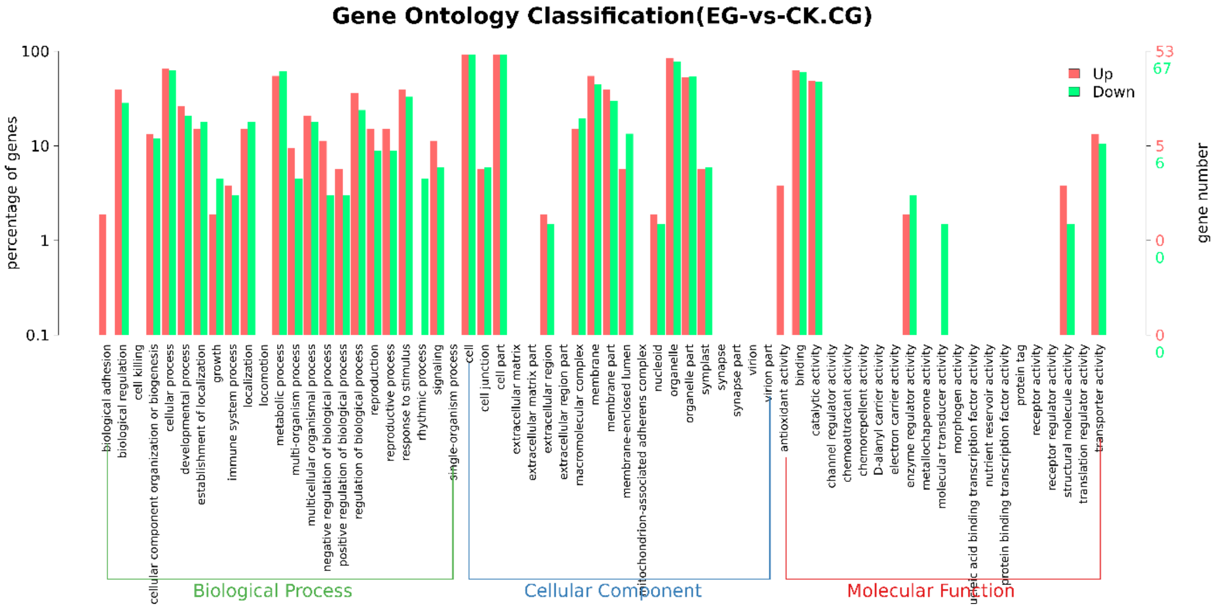

B

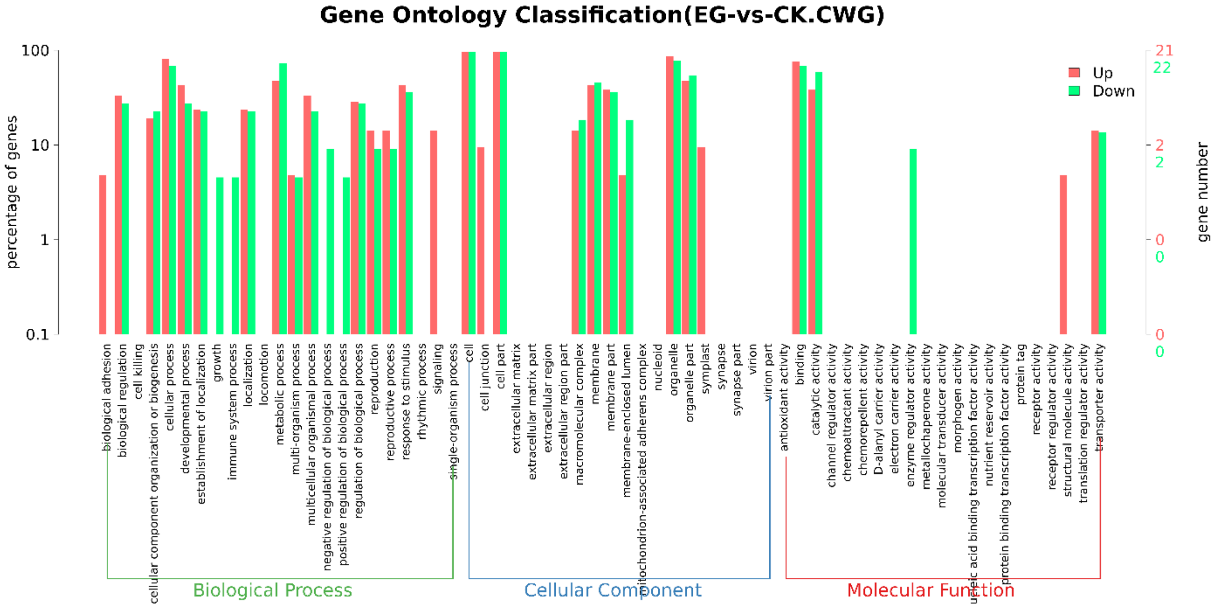

Figure S12. A comparison of the GO classification where the DEGs were either up or downregulated in both CG and CWG; (**A**) classification at the CG sites; (**B**) classification at the CWG sites

A

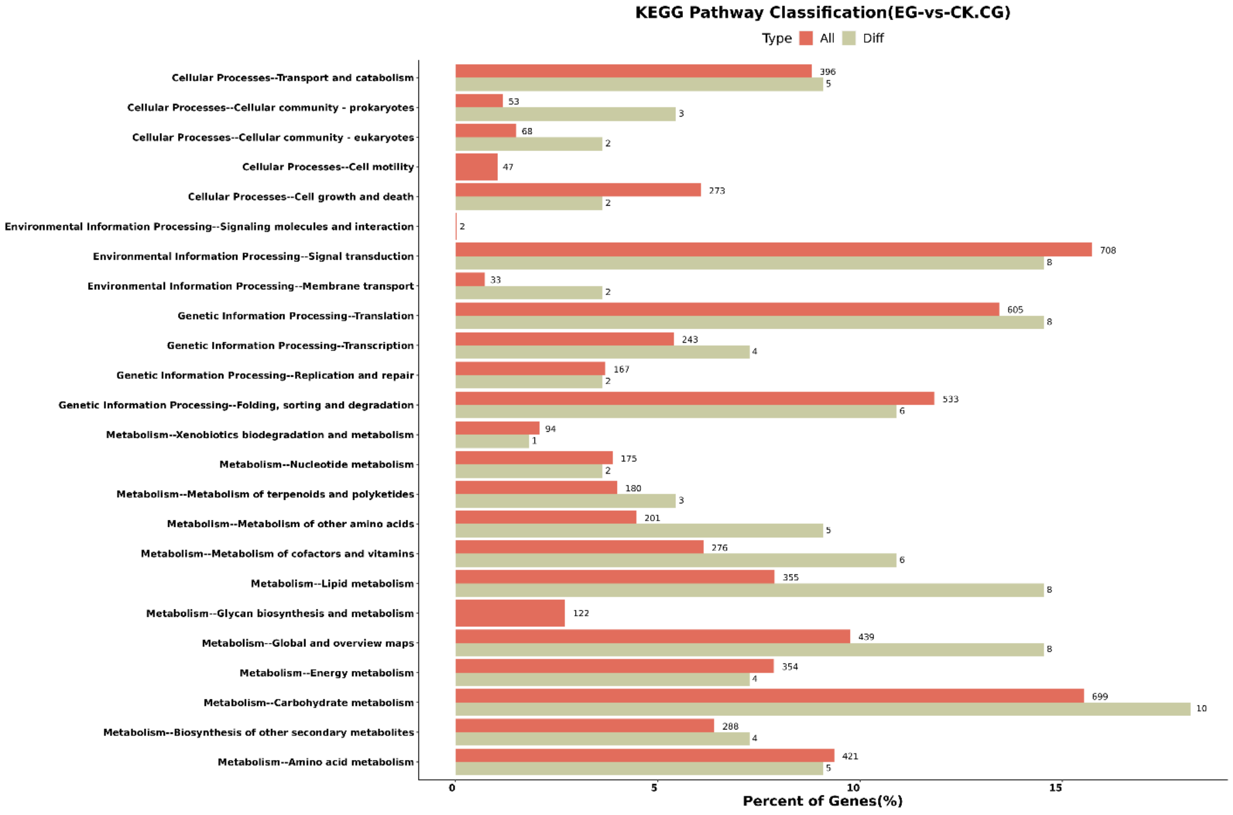

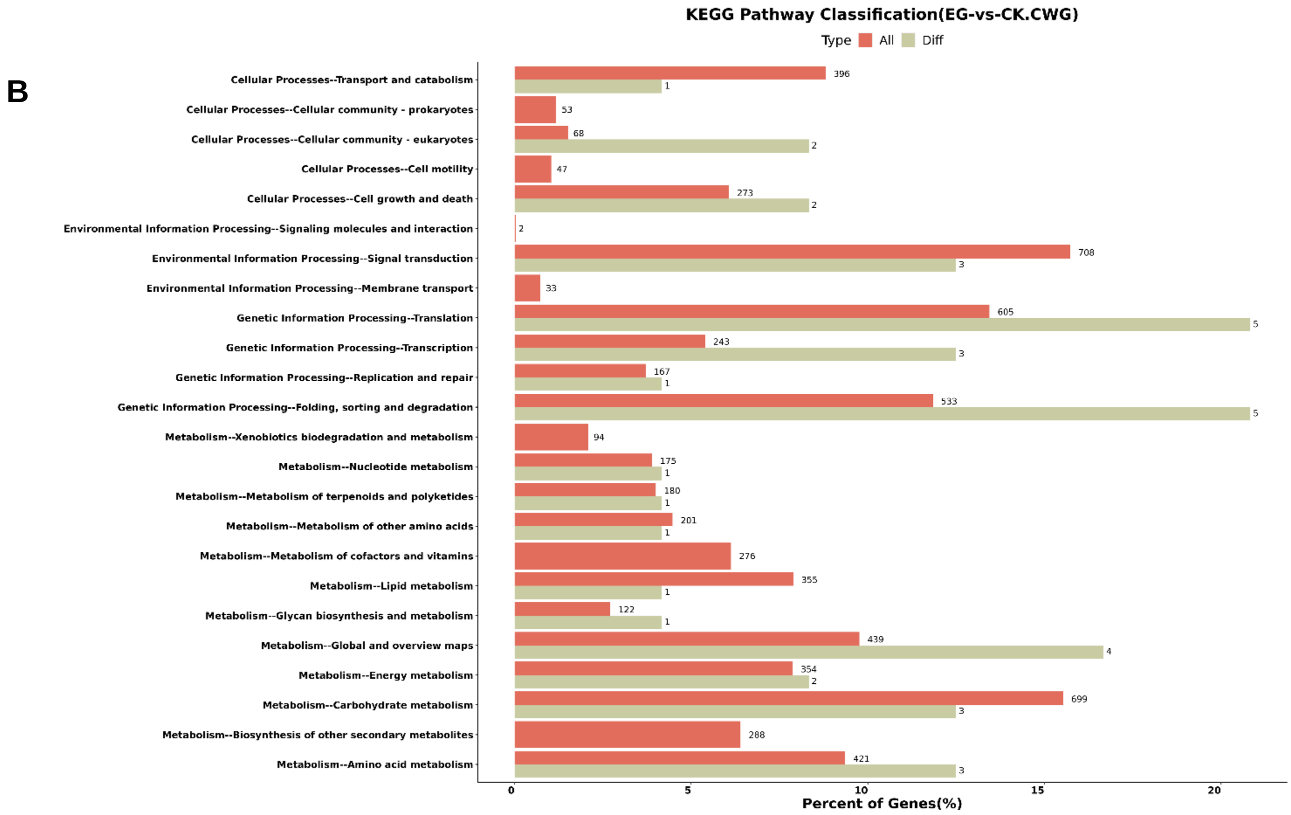

Figure S13. Comparison of the distribution of DEGs and all genes at the KEGG Level where the differential methylation sites are located. (**A**) KEGG pathway classification at the CG level; (**B**) KEGG pathway classification at the CWG level

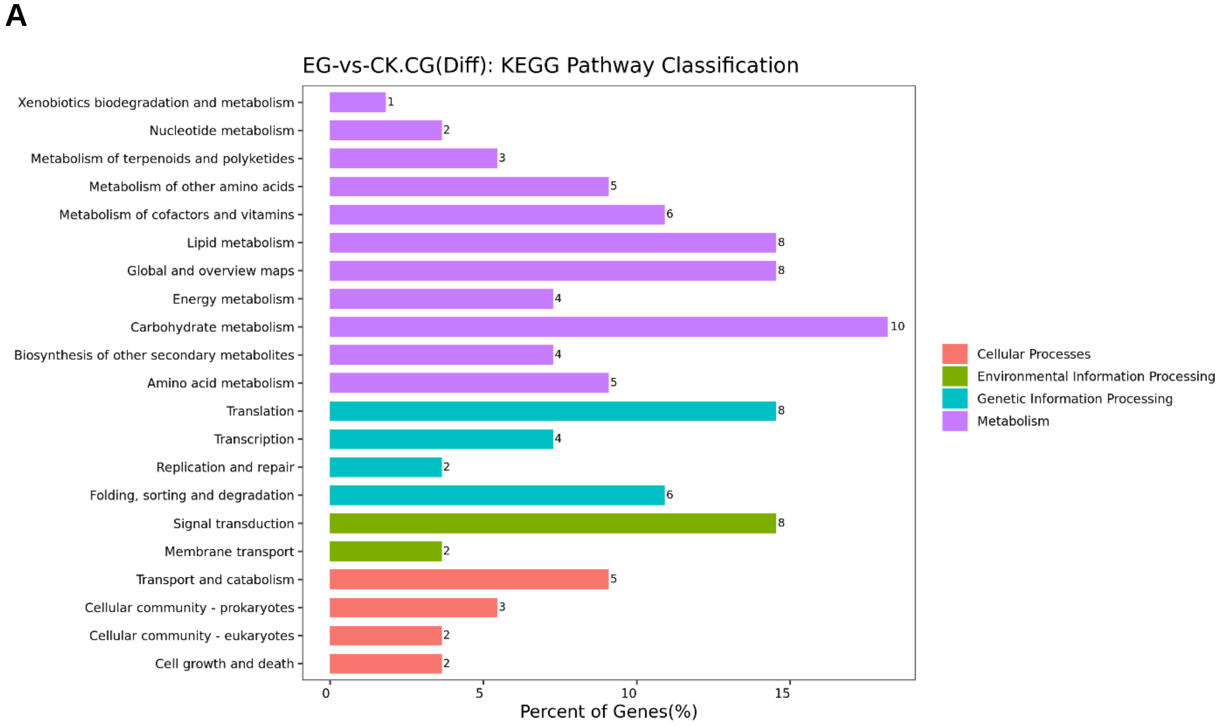

B

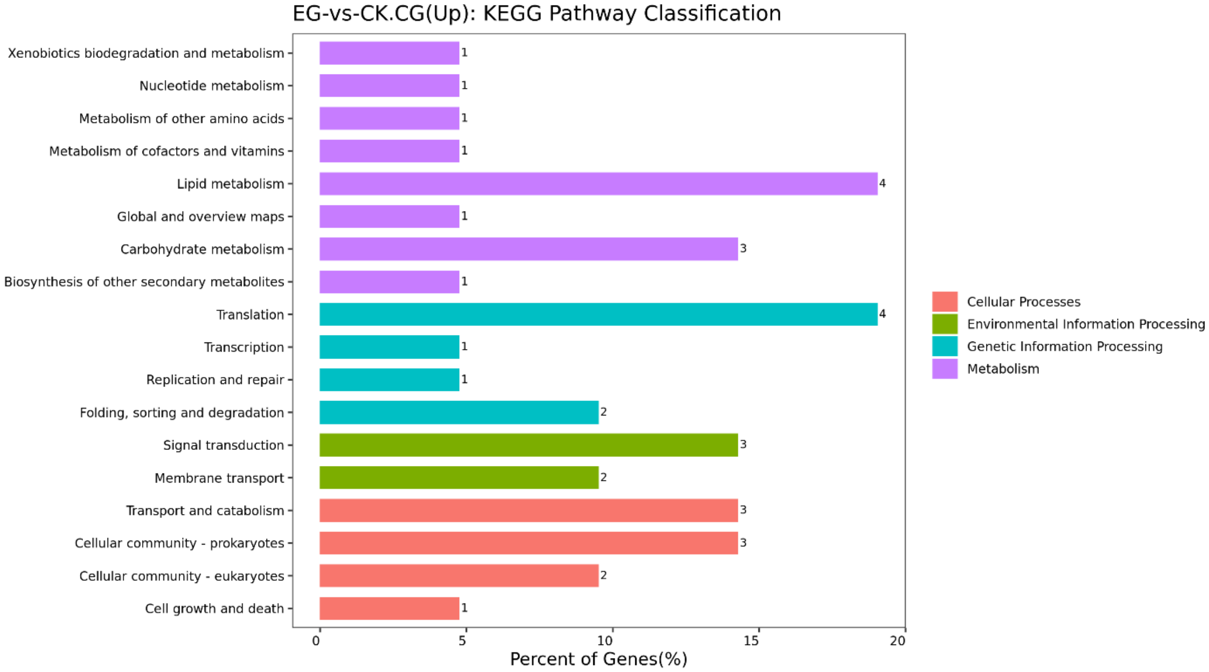
EG-vs-CK.CG(Up): KEGG Pathway Classification
Xenobiotics biodegradation and metabolism
Nucleotide metabolism
Metabolism of other amino acids
Metabolism of cofactors and vitamins
Lipid metabolism
Global and overview maps
Carbohydrate metabolism
Biosynthesis of other secondary metabolites
Translation
Transcription
Replication and repair
Folding, sorting and degradation
Signal transduction
Membrane transport
Transport and catabolism
Cellular community - prokaryotes
Cellular community - eukaryotes
Cell growth and death
1
1
1
1
4
1
3
1
4
1
1
2
3
2
3
3
2
1
0
5
10
15
20
Percent of Genes(%)
Cellular Processes
Environmental Information Processing
Genetic Information Processing
Metabolism

C

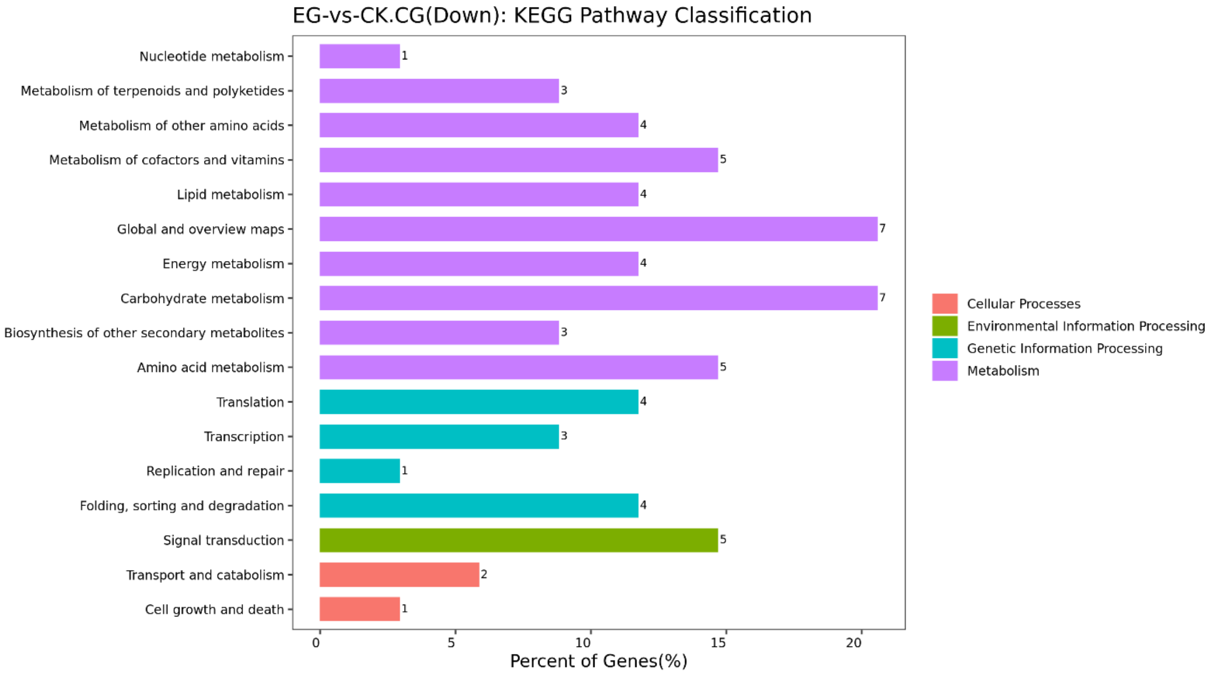
EG-vs-CK.CG(Down): KEGG Pathway Classification
Nucleotide metabolism
Metabolism of terpenoids and polyketides
Metabolism of other amino acids
Metabolism of cofactors and vitamins
Lipid metabolism
Global and overview maps
Energy metabolism
Carbohydrate metabolism
Biosynthesis of other secondary metabolites
Amino acid metabolism
Translation
Transcription
Replication and repair
Folding, sorting and degradation
Signal transduction
Transport and catabolism
Cell growth and death
1
3
4
5
4
7
4
7
3
5
4
3
1
4
5
2
1
0
5
10
15
20
Percent of Genes(%)
Cellular Processes
Environmental Information Processing
Genetic Information Processing
Metabolism

Figure S14. Classification of DEGs associated with the DMS at the CG methylation involved in the KEGG pathways (**A**) Classification of DEGs involved in the pathway. (**B**, **C**) Classification of up and downregulated DEGs involved in the KEGG pathway

A

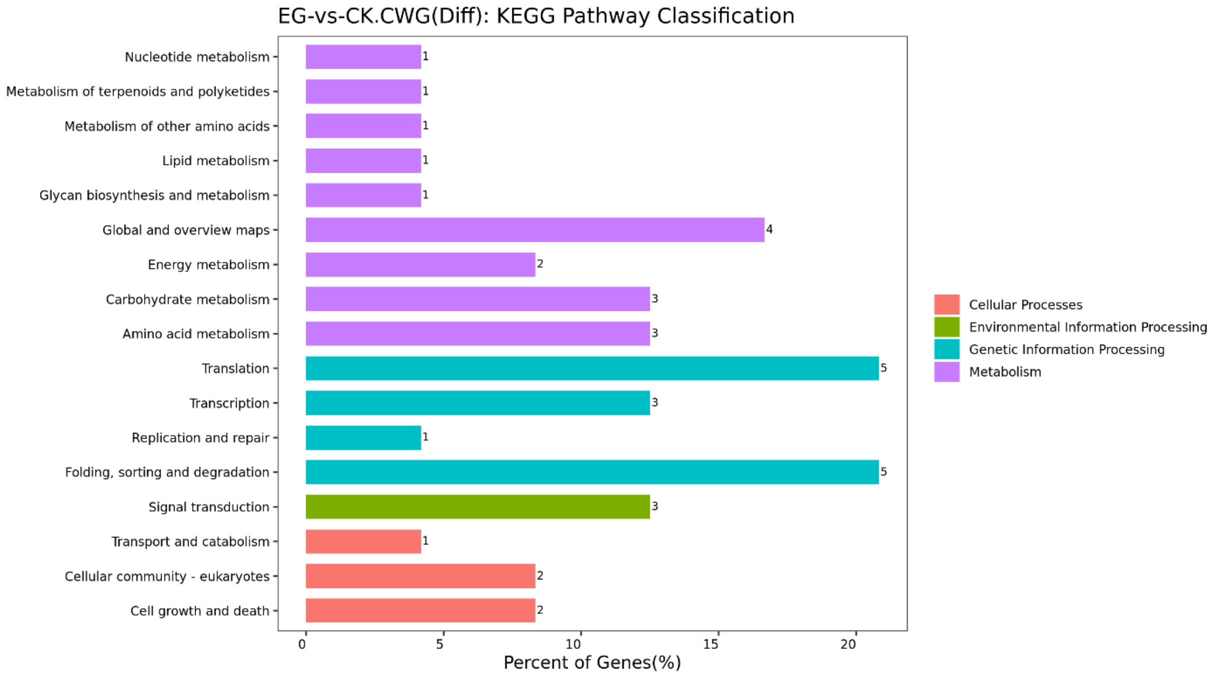

B

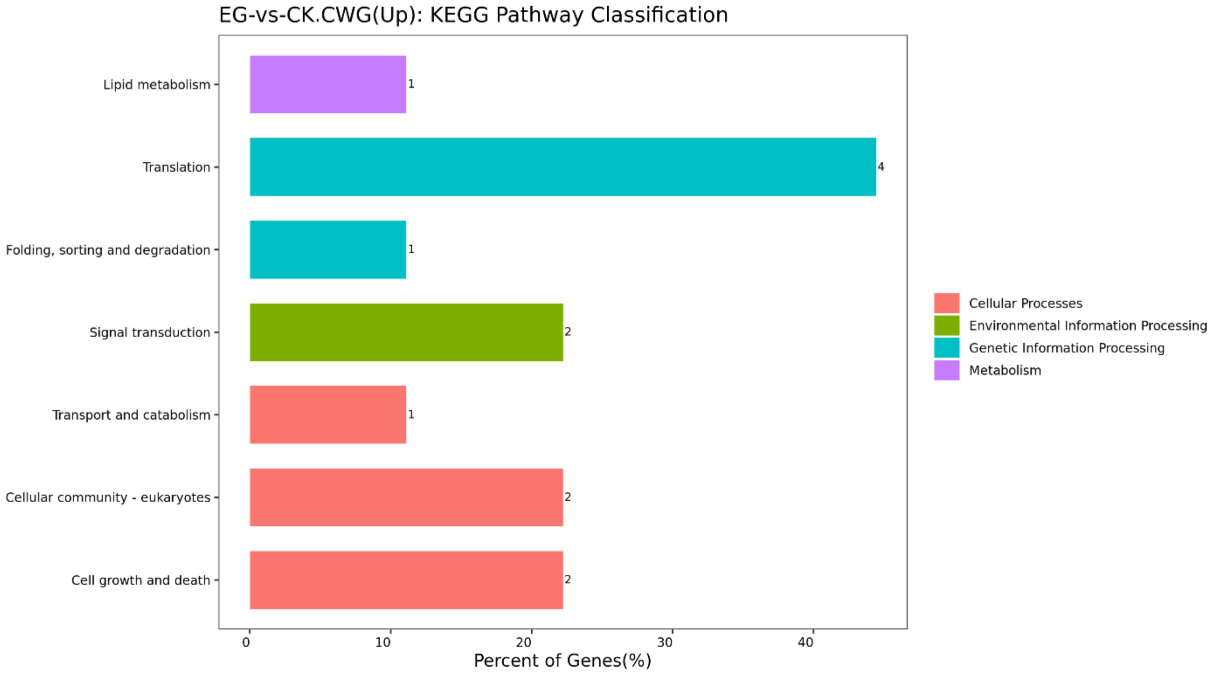

C

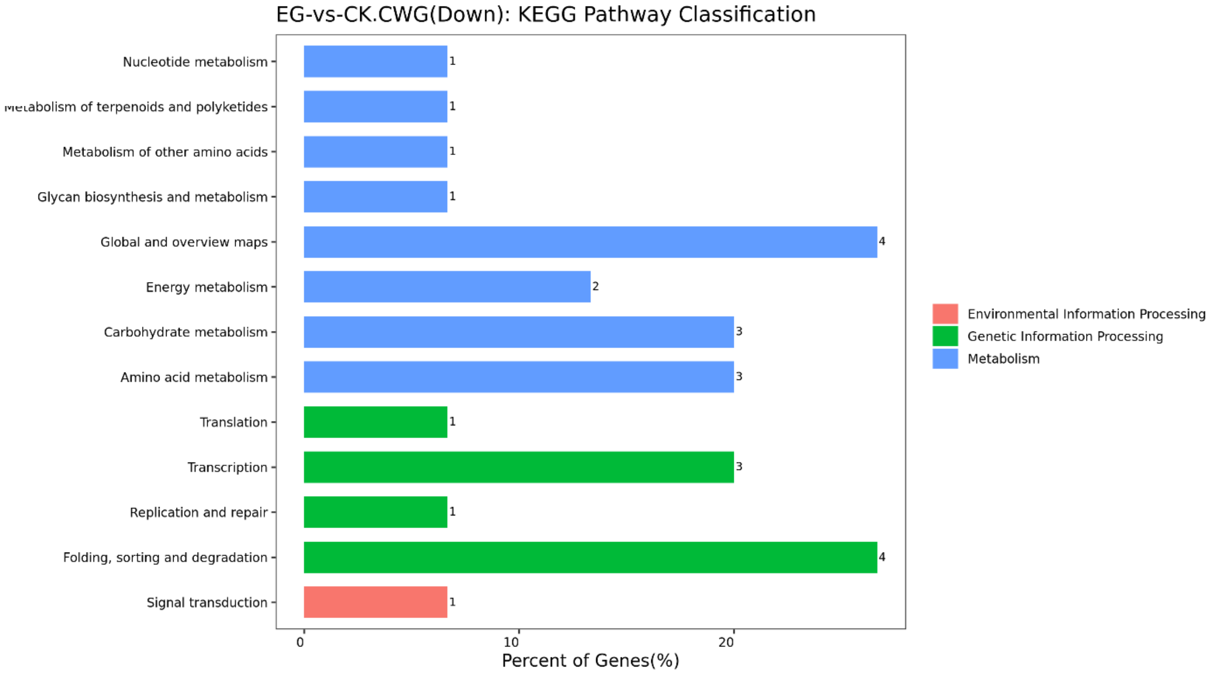

Figure S15. Classification of DEGs associated with the DMS at the CWG methylation involved in the KEGG pathways (**A**) Classification of DEGs involved in the pathway. (**B**, **C**) Classification of DEGs involved in up and downregulated CWG sites

A

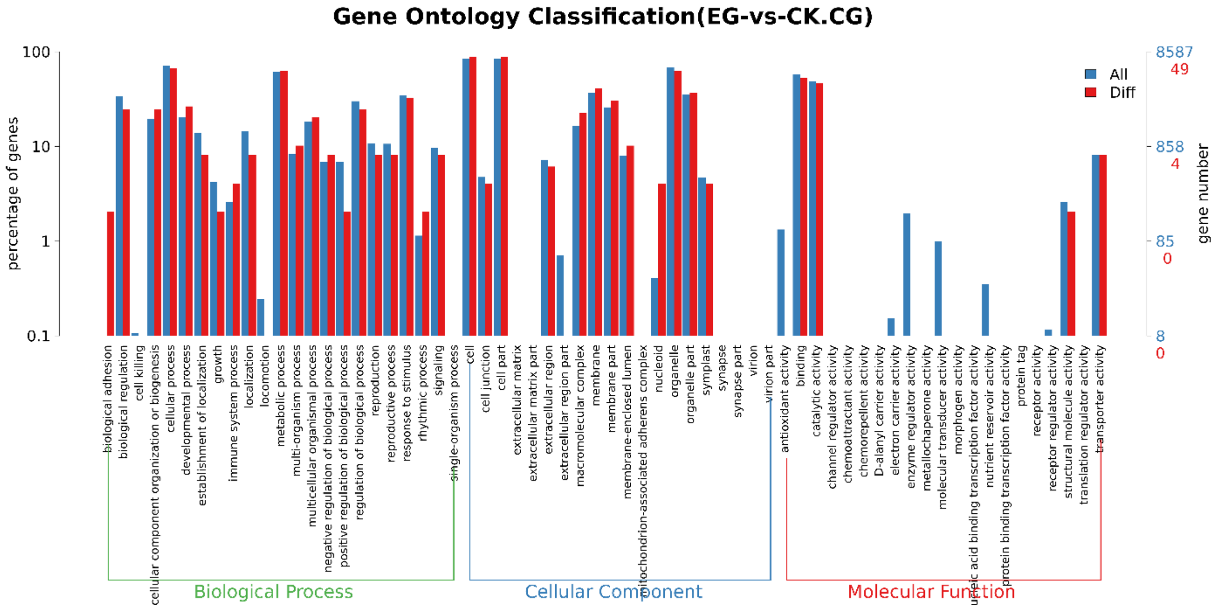

B

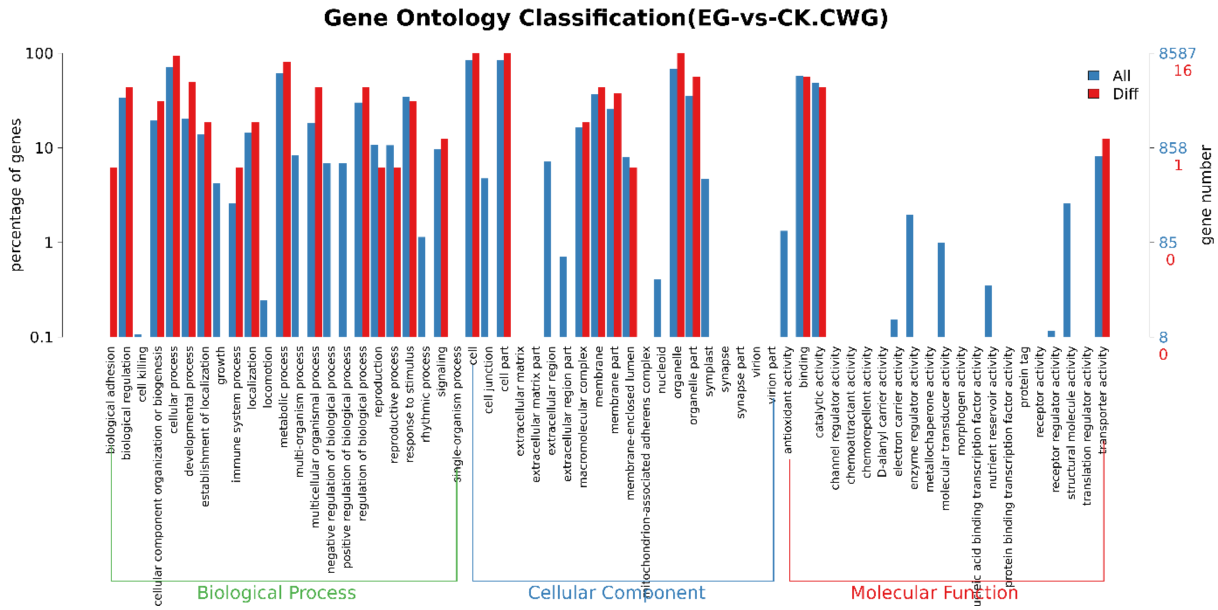

Figure S16. A comparison of the distribution of DMGs. (**A**) GO classification at the CG methylation site. (**B**) GO classification at the CWG methylation site. Columns in blue indicate GO entries for all gene enrichment.the columns in red are the GO entries for differential expression gene enrichment, the horizontal axis is the GO entry names, and the vertical axis is the corresponding DEGs genes and their percentage

A

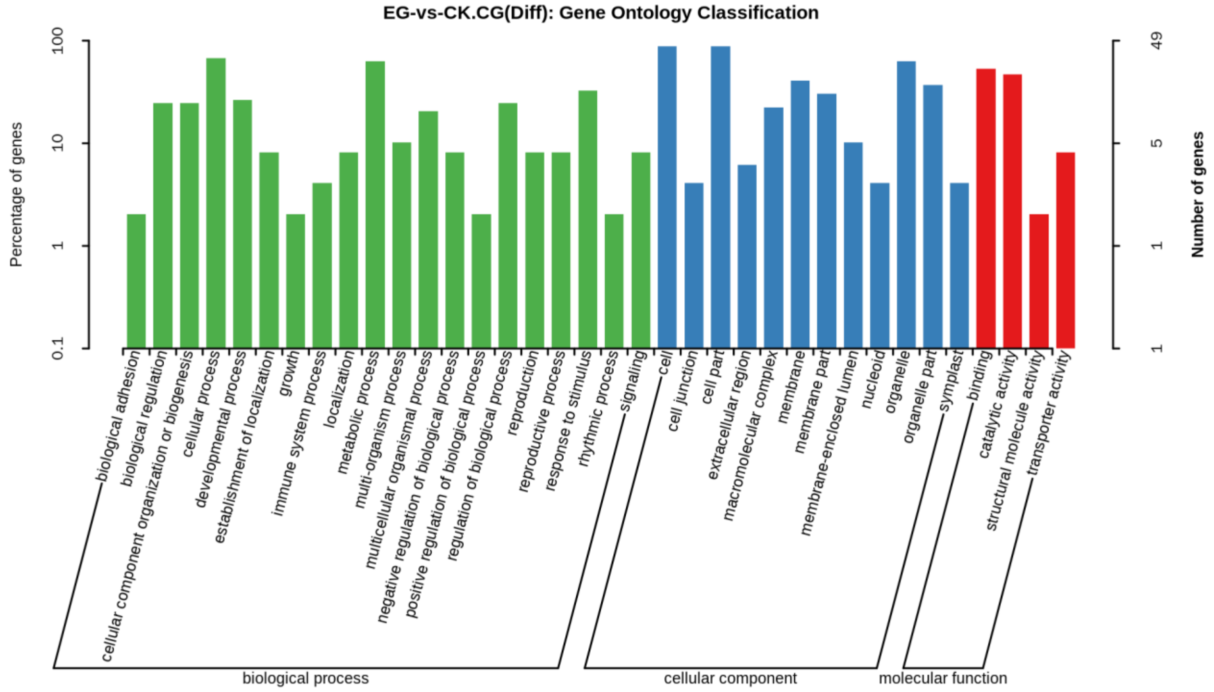

.

B

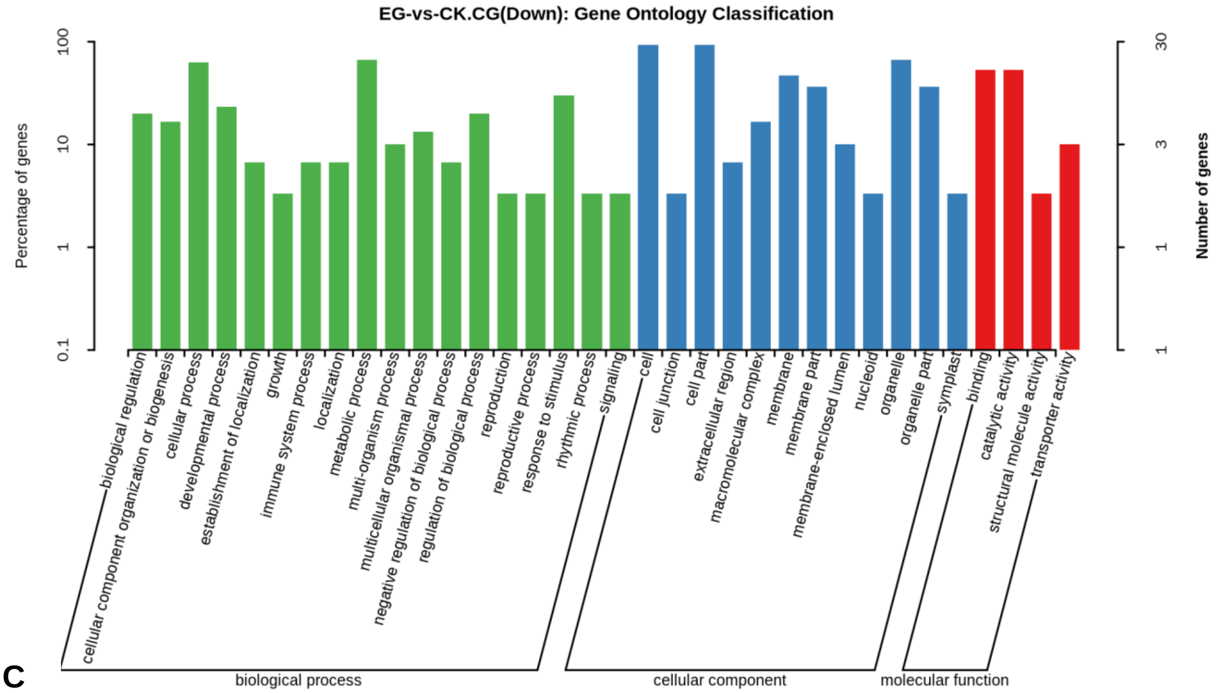
EG-vs-CK.CG(Down): Gene Ontology Classification
Percentage of genes
Number of genes
100
10
1
0.1
30
3
1
1
biological regulation
cellular component organization or biogenesis
cellular process
developmental process
establishment of localization
growth
immune system process
localization
metabolic process
multi-organism process
multicellular organismal process
negative regulation of biological process
regulation of biological process
reproduction
reproductive process
response to stimulus
rhythmic process
signaling
cell
cell junction
cell part
extracellular region
macromolecular complex
membrane
membrane part
membrane-enclosed lumen
nucleoid
organelle
organelle part
symplast
binding
catalytic activity
structural molecule activity
transporter activity
biological process
cellular component
molecular function

C

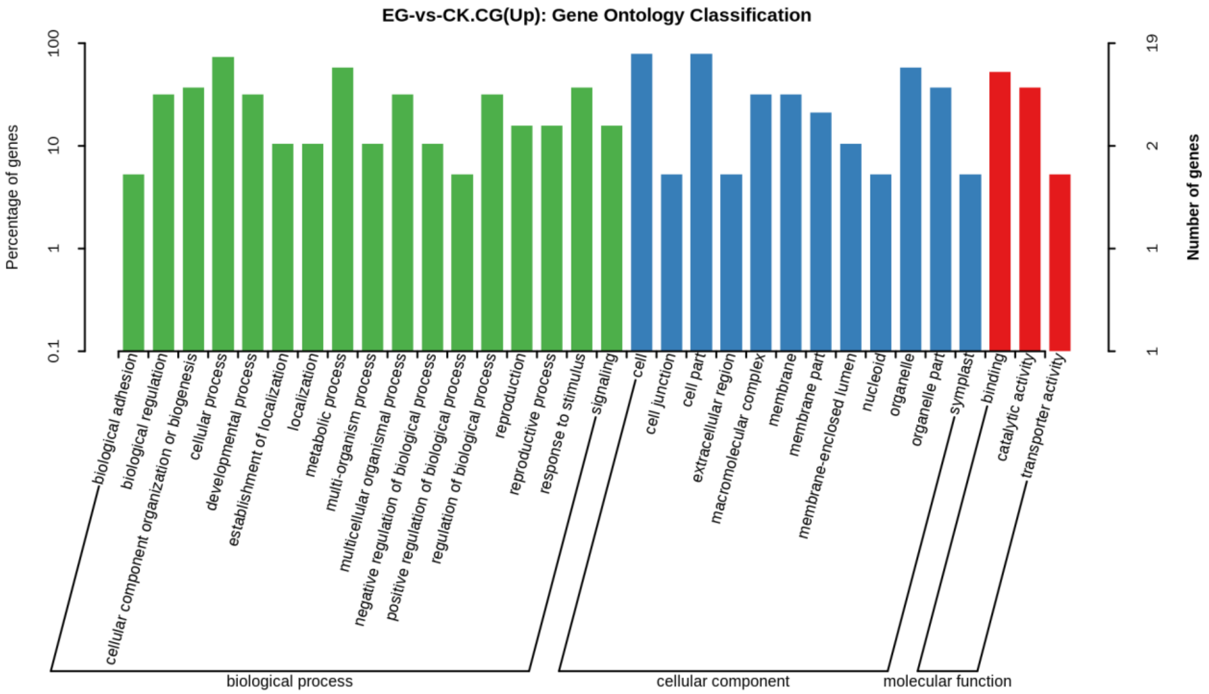
EG-vs-CK.CG(Up): Gene Ontology Classification
Percentage of genes
Number of genes
100
10
1
0.1
19
2
1
1
biological adhesion
biological regulation
cellular component organization or biogenesis
cellular process
developmental process
establishment of localization
localization
metabolic process
multi-organism process
multicellular organismal process
negative regulation of biological process
positive regulation of biological process
regulation of biological process
reproduction
reproductive process
response to stimulus
signaling
cell
cell junction
cell part
extracellular region
macromolecular complex
membrane
membrane part
membrane-enclosed lumen
nucleoid
organelle
organelle part
symplast
binding
catalytic activity
transporter activity
biological process
cellular component
molecular function

D

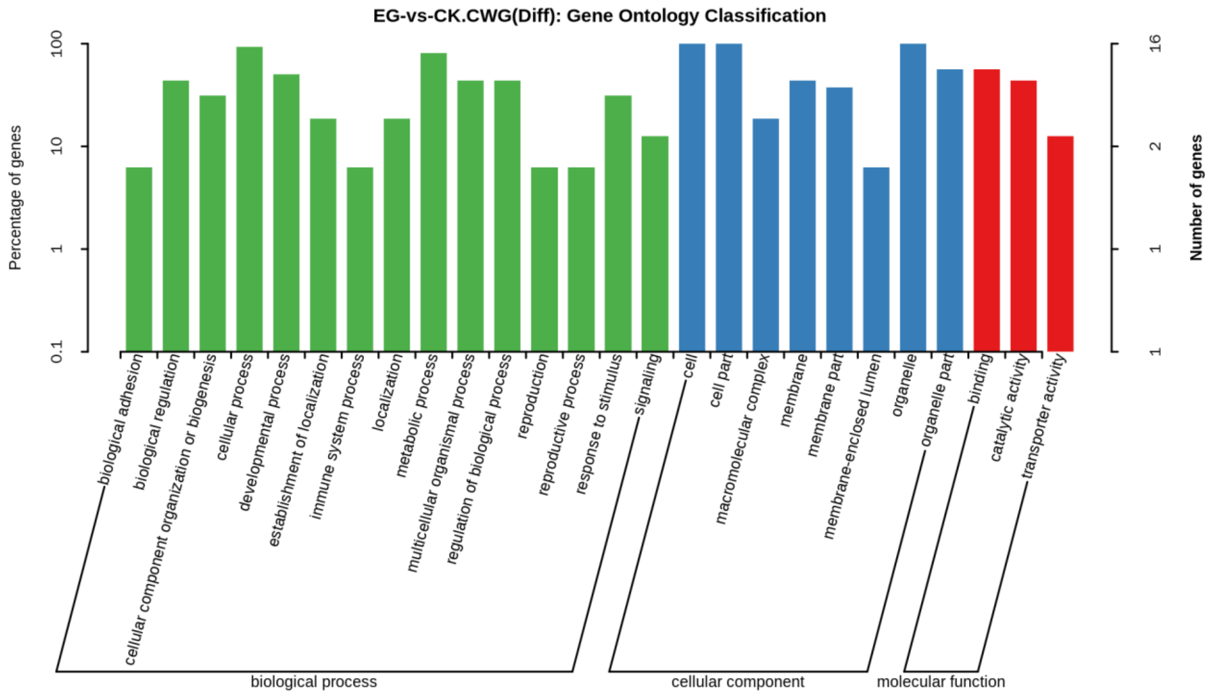

EG-vs-CK.CWG(Diff): Gene Ontology Classification
Percentage of genes
100
10
1
0.1
Number of genes
16
2
1
1
biological adhesion
biological regulation
cellular component organization or biogenesis
cellular process
developmental process
establishment of localization
immune system process
localization
metabolic process
multicellular organismal process
regulation of biological process
reproduction
reproductive process
response to stimulus
signaling
cell
cell part
macromolecular complex
membrane
membrane part
membrane-enclosed lumen
organelle
organelle part
binding
catalytic activity
transporter activity
biological process
cellular component
molecular function

E

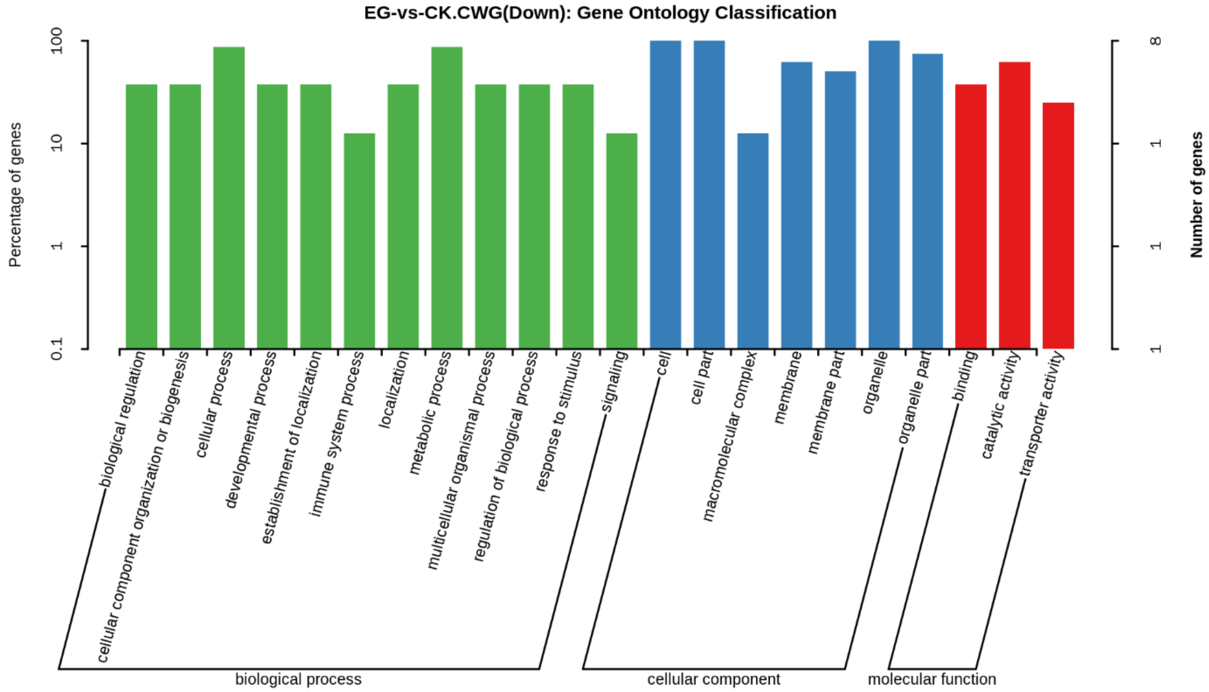

EG-vs-CK.CWG(Down): Gene Ontology Classification
Percentage of genes
100
10
1
0.1
Number of genes
8
1
1
1
biological regulation
cellular component organization or biogenesis
cellular process
developmental process
establishment of localization
immune system process
localization
metabolic process
multicellular organismal process
regulation of biological process
response to stimulus
signaling
cell
cell part
macromolecular complex
membrane
membrane part
organelle
organelle part
binding
catalytic activity
transporter activity
biological process
cellular component
molecular function

F

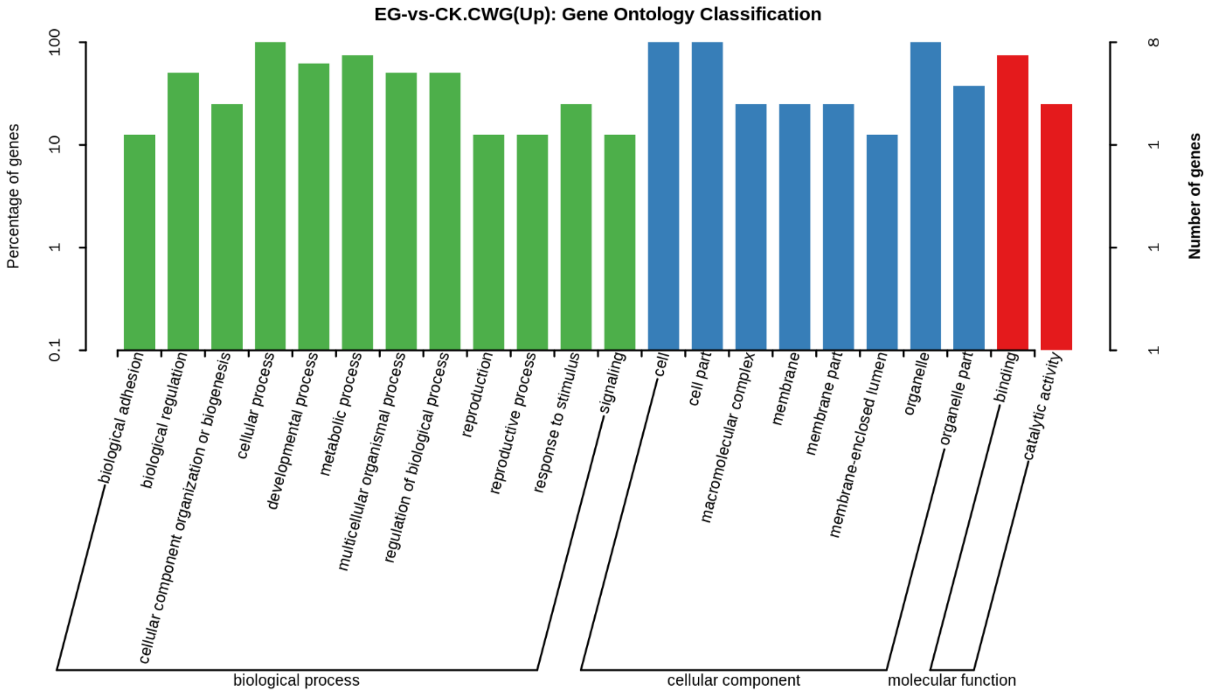

Figure S17. Comparison of the GO classification of the DMGs where the mCG and mCWG sites are located. (**A**) GO classification of the DMGs at the mCG site; (**B**,**C**) The DMGs in the GO terms at the down and upregulated mCG sites; (D) GO classification of the DMGs at the mCWG site; (**E**,**F**) the DMGs in the GO terms at the down and upregulated mCWG sites

A

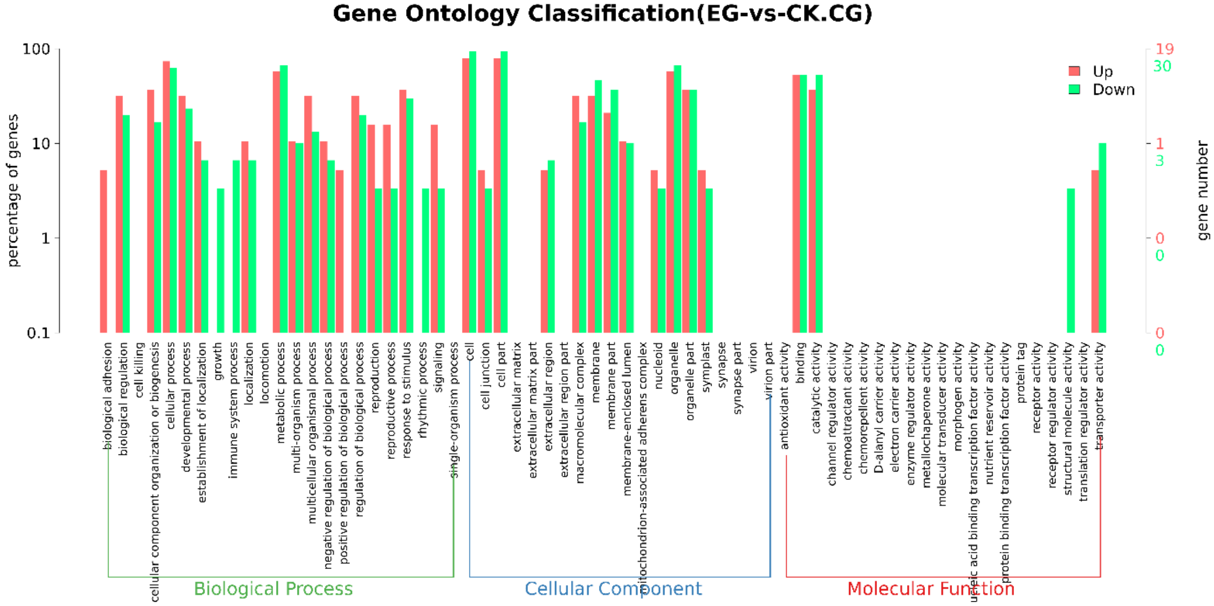

B

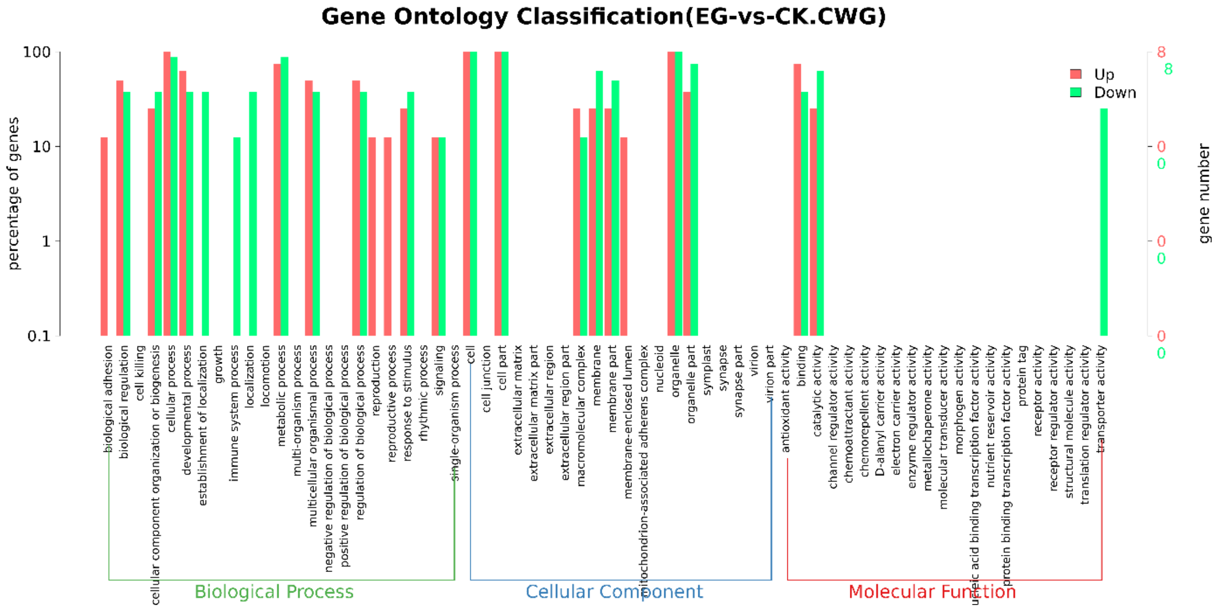

Figure S18. Comparison of the GO classification where the DMGs were at either up or downregulated mCG and mCWG sites. (**A**) classification at the mCG sites; (**B**) classification at the mCWG sites

A

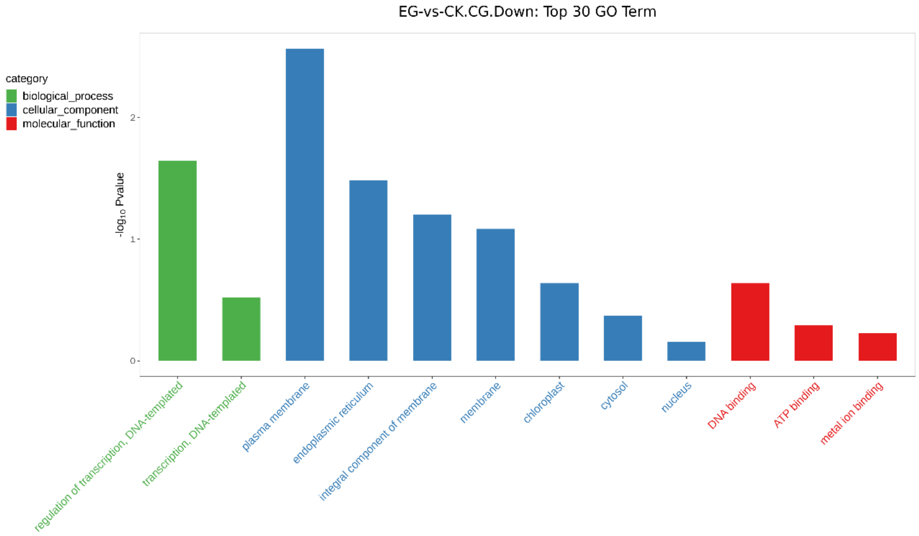

B

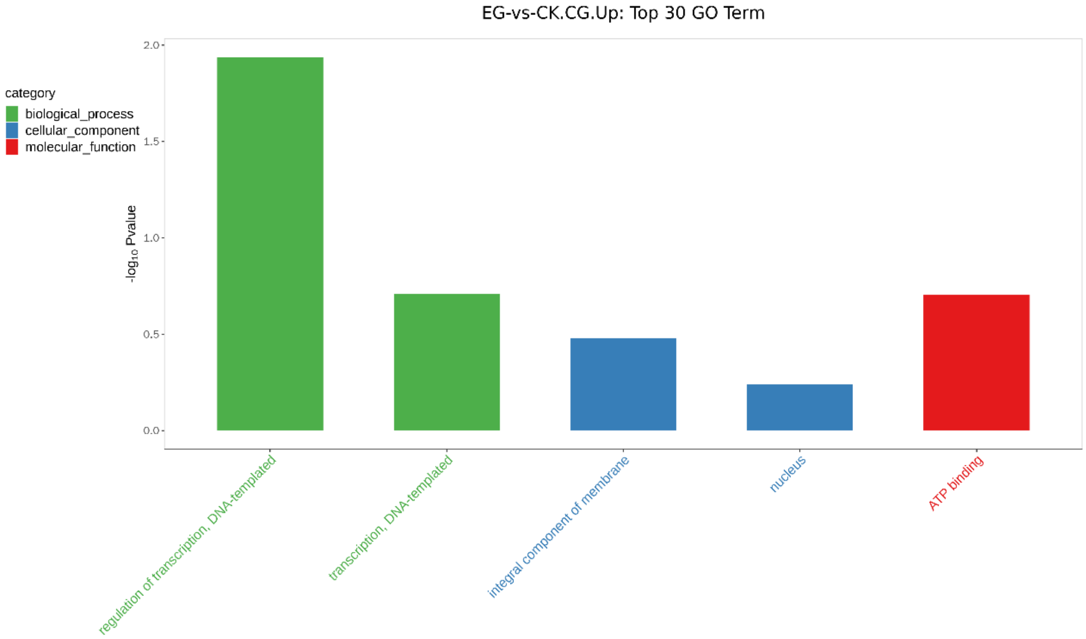
EG-vs-CK.CG.Up: Top 30 GO Term
category
biological_process
cellular_component
molecular_function
-log10 Pvalue
2.0
1.5
1.0
0.5
0.0
regulation of transcription, DNA-templated
transcription, DNA-templated
integral component of membrane
nucleus
ATP binding

C

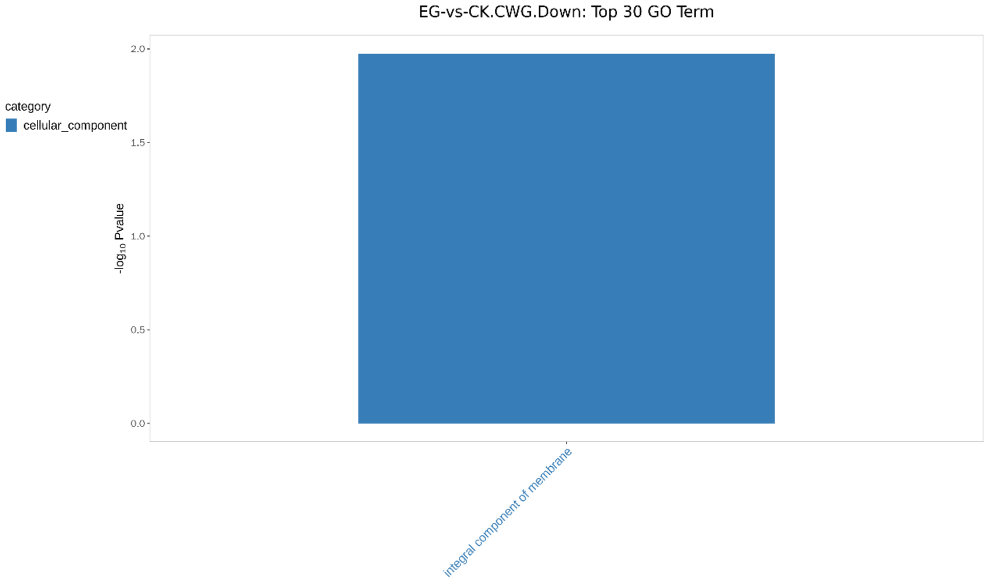
EG-vs-CK.CWG.Down: Top 30 GO Term
category
cellular_component
-log10 Pvalue
2.0
1.5
1.0
0.5
0.0
integral component of membrane

D

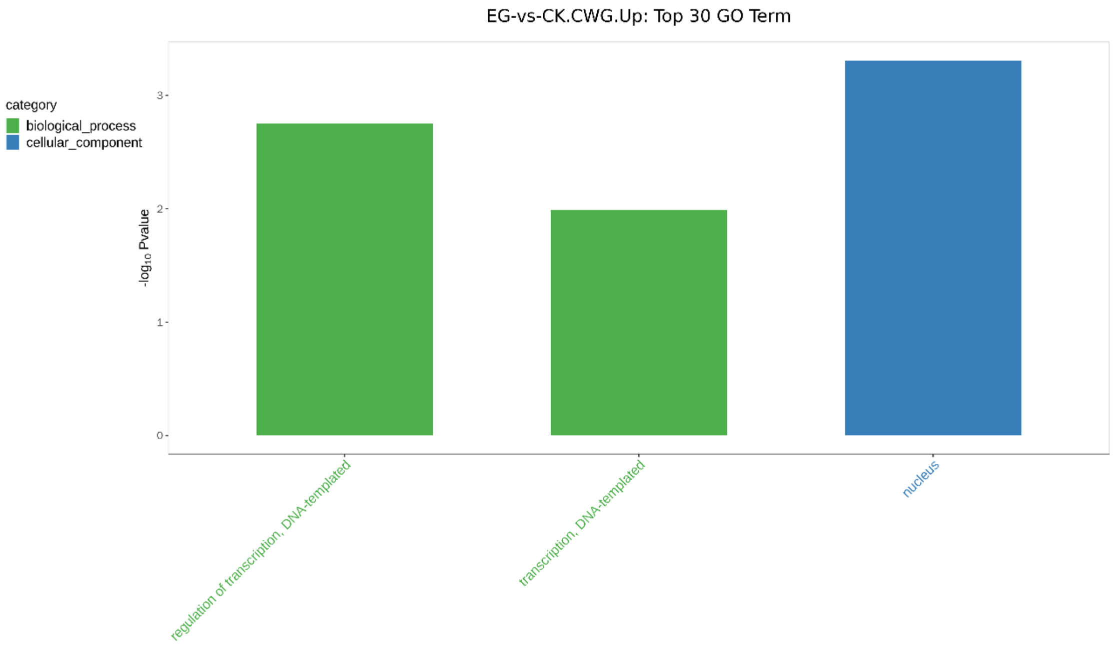

Figure 19. A bar chart of the top 30 GO functions of the DMGs at the mCG and mCWG sites. (**A**) the top 30 downregulated at mCG sites; (**B**) the top 30 upregulated at the mCG sites; (**C**) the top 30 downregulated at mCWG sites; (**D**) the top 30 upregulated at mCWG sites. The X coordinates are the name of the GO entry, and the Y coordinates are -log10Pvalue.

A

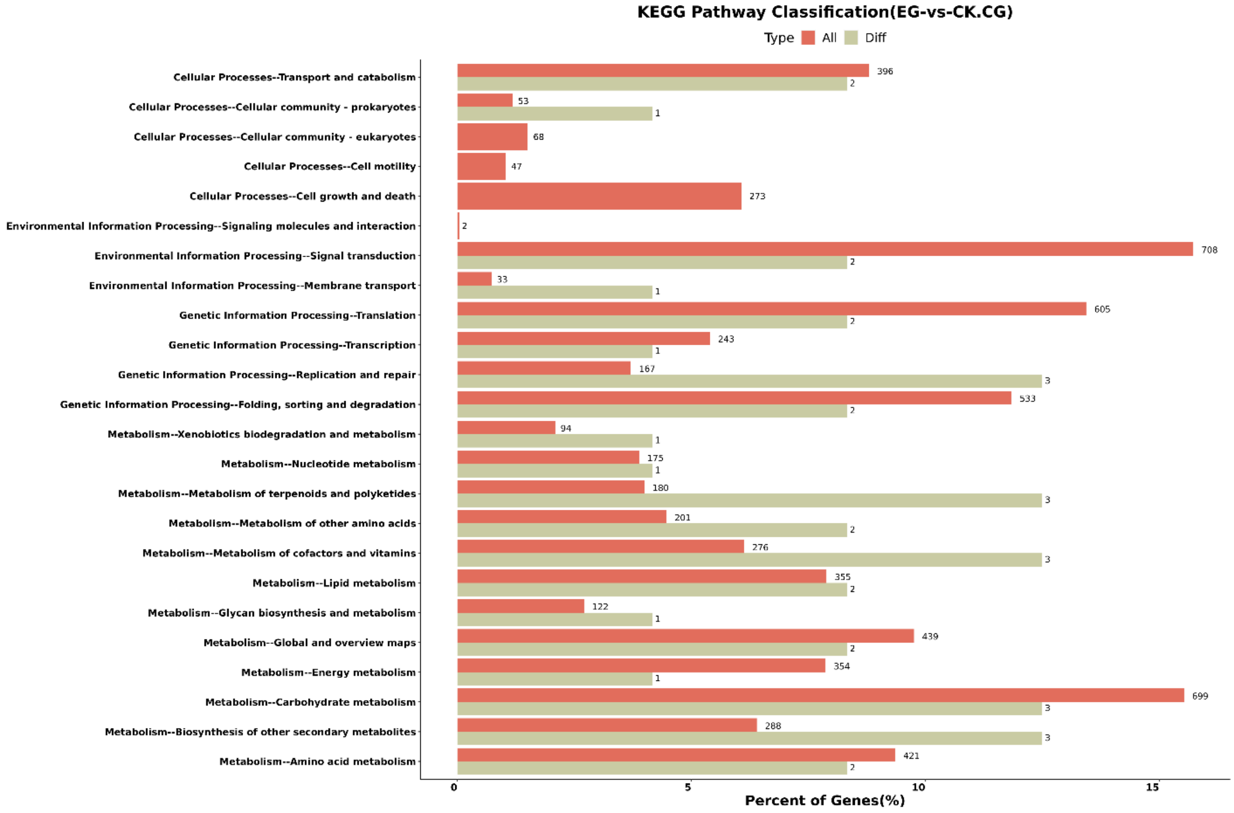

KEGG Pathway Classification(EG-vs-CK.CG)
Type All Diff
Cellular Processes--Transport and catabolism
Cellular Processes--Cellular community - prokaryotes
Cellular Processes--Cellular community - eukaryotes
Cellular Processes--Cell motility
Cellular Processes--Cell growth and death
Environmental Information Processing--Signaling molecules and interaction
Environmental Information Processing--Signal transduction
Environmental Information Processing--Membrane transport
Genetic Information Processing--Translation
Genetic Information Processing--Transcription
Genetic Information Processing--Replication and repair
Genetic Information Processing--Folding, sorting and degradation
Metabolism--Xenobiotics biodegradation and metabolism
Metabolism--Nucleotide metabolism
Metabolism--Metabolism of terpenoids and polyketides
Metabolism--Metabolism of other amino acids
Metabolism--Metabolism of cofactors and vitamins
Metabolism--Lipid metabolism
Metabolism--Glycan biosynthesis and metabolism
Metabolism--Global and overview maps
Metabolism--Energy metabolism
Metabolism--Carbohydrate metabolism
Metabolism--Biosynthesis of other secondary metabolites
Metabolism--Amino acid metabolism
0 5 10 15
Percent of Genes(%)

B

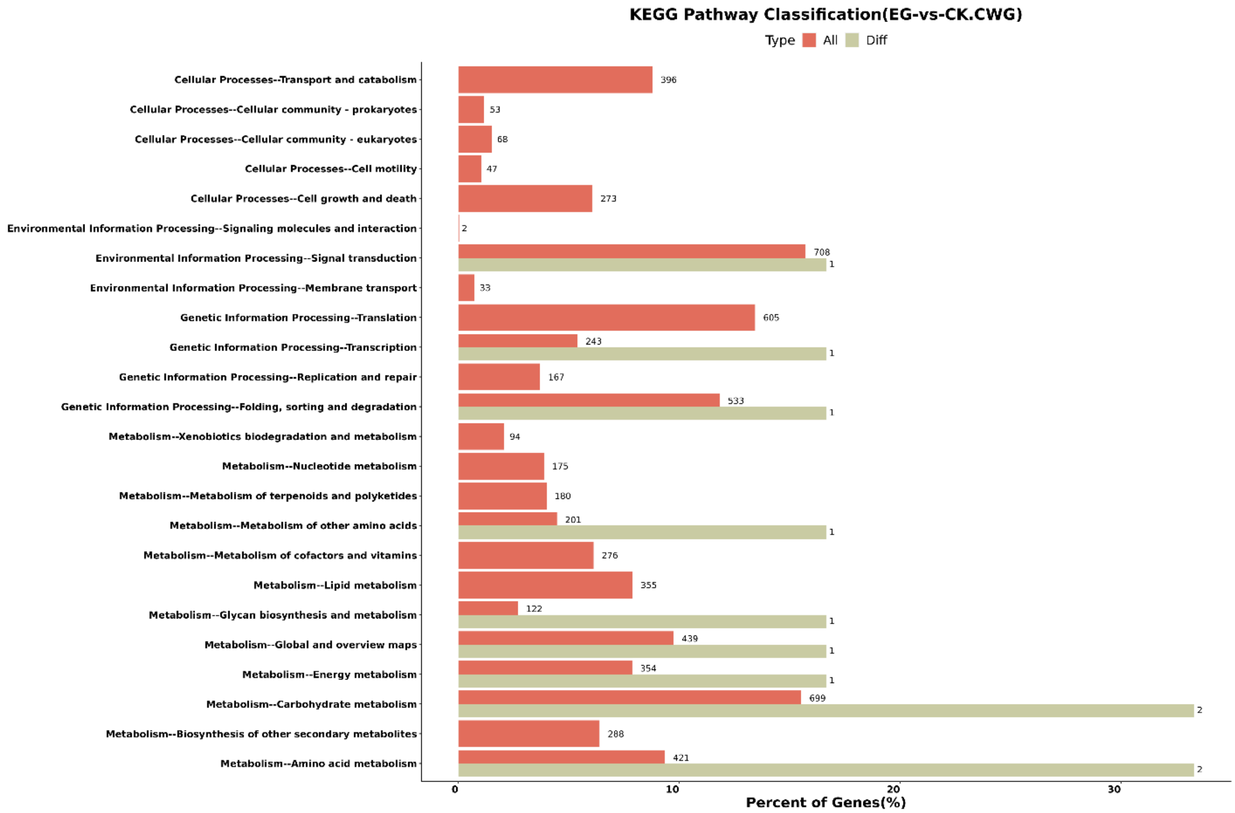

KEGG Pathway Classification(EG-vs-CK.CWG)
Type All Diff
Cellular Processes--Transport and catabolism
Cellular Processes--Cellular community - prokaryotes
Cellular Processes--Cellular community - eukaryotes
Cellular Processes--Cell motility
Cellular Processes--Cell growth and death
Environmental Information Processing--Signaling molecules and interaction
Environmental Information Processing--Signal transduction
Environmental Information Processing--Membrane transport
Genetic Information Processing--Translation
Genetic Information Processing--Transcription
Genetic Information Processing--Replication and repair
Genetic Information Processing--Folding, sorting and degradation
Metabolism--Xenobiotics biodegradation and metabolism
Metabolism--Nucleotide metabolism
Metabolism--Metabolism of terpenoids and polyketides
Metabolism--Metabolism of other amino acids
Metabolism--Metabolism of cofactors and vitamins
Metabolism--Lipid metabolism
Metabolism--Glycan biosynthesis and metabolism
Metabolism--Global and overview maps
Metabolism--Energy metabolism
Metabolism--Carbohydrate metabolism
Metabolism--Biosynthesis of other secondary metabolites
Metabolism--Amino acid metabolism
0 10 20 30
Percent of Genes(%)

Figure S20. Comparison of the distribution of DMGs and all genes at the KEGG Level. (**A**) KEGG pathway classification at the mCG sites. (**B**) KEGG pathway classification at the mCWG sites.

A

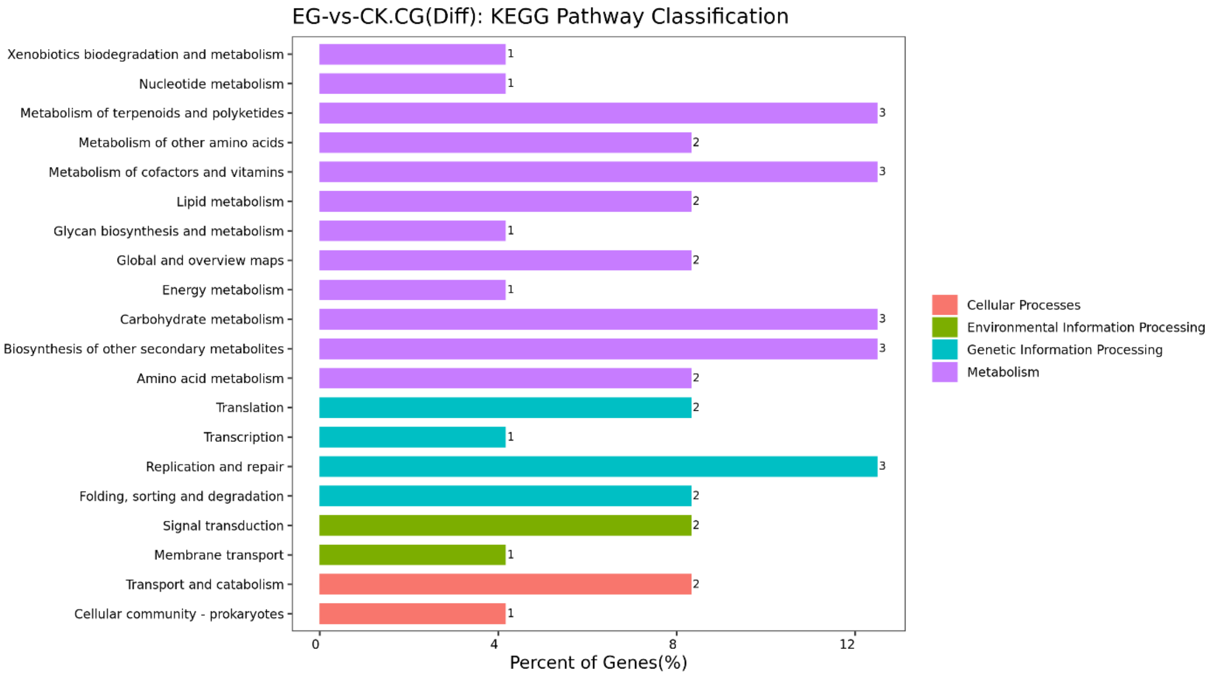

B

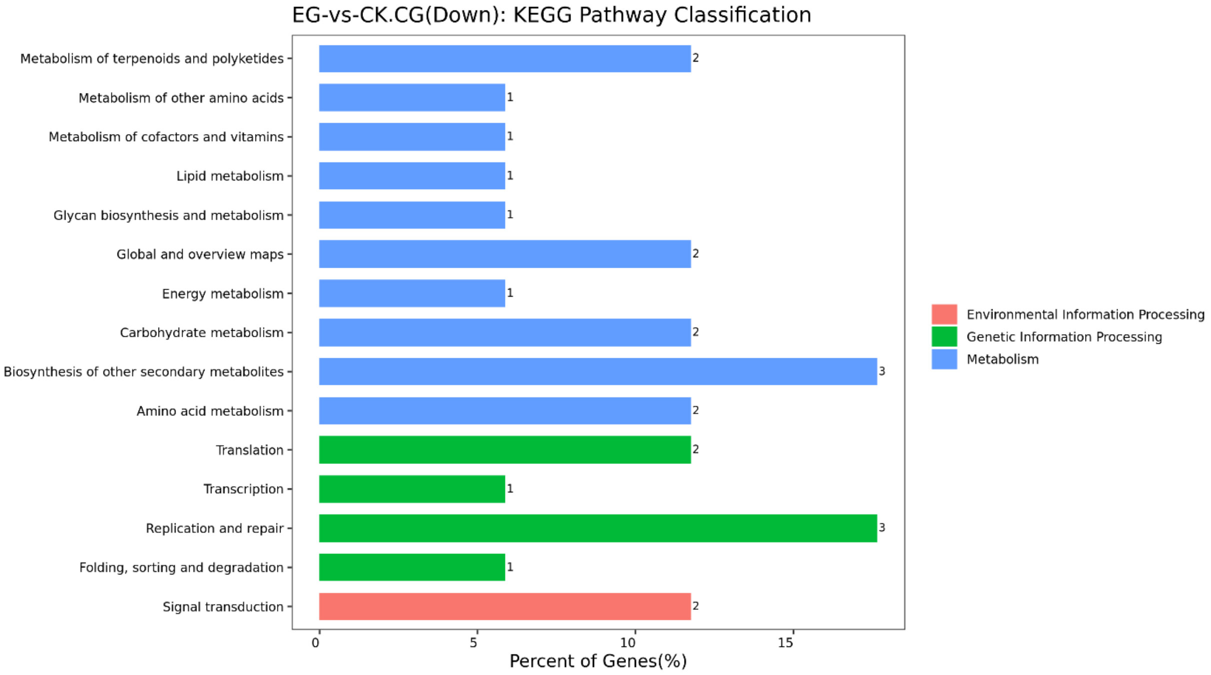

C

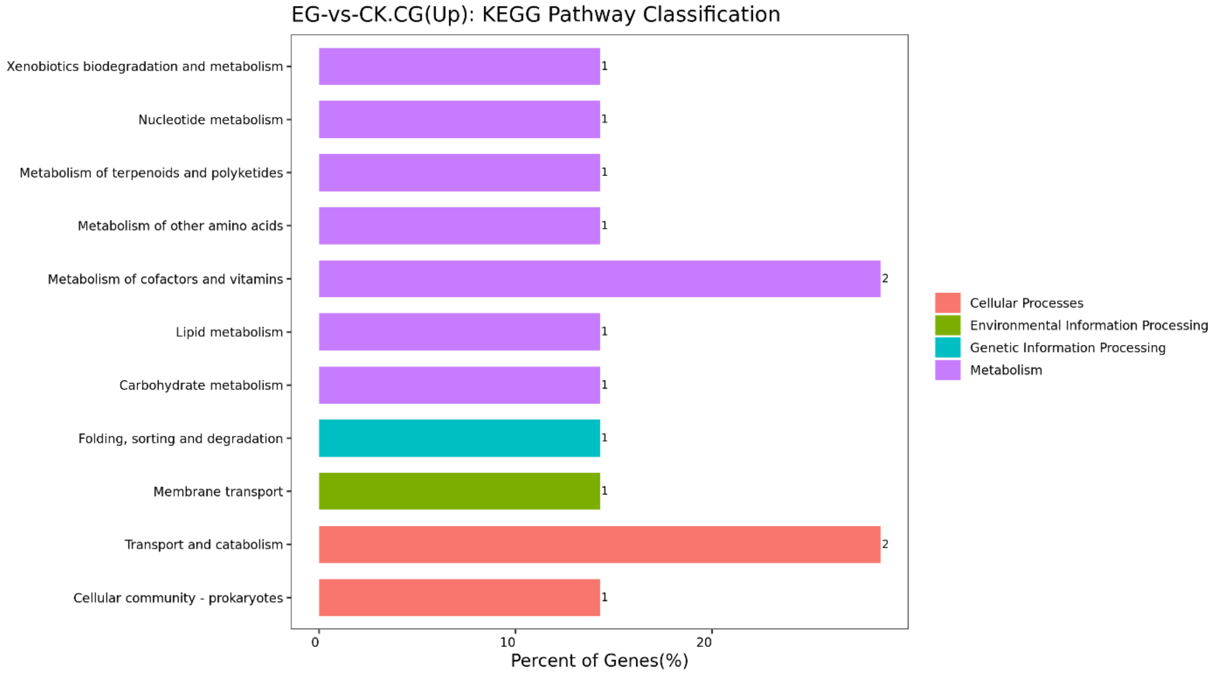
EG-vs-CK.CG(Up): KEGG Pathway Classification
Xenobiotics biodegradation and metabolism
Nucleotide metabolism
Metabolism of terpenoids and polyketides
Metabolism of other amino acids
Metabolism of cofactors and vitamins
Lipid metabolism
Carbohydrate metabolism
Folding, sorting and degradation
Membrane transport
Transport and catabolism
Cellular community - prokaryotes
1
1
1
1
2
1
1
1
1
2
1
0
10
20
Percent of Genes(%)
Cellular Processes
Environmental Information Processing
Genetic Information Processing
Metabolism

D

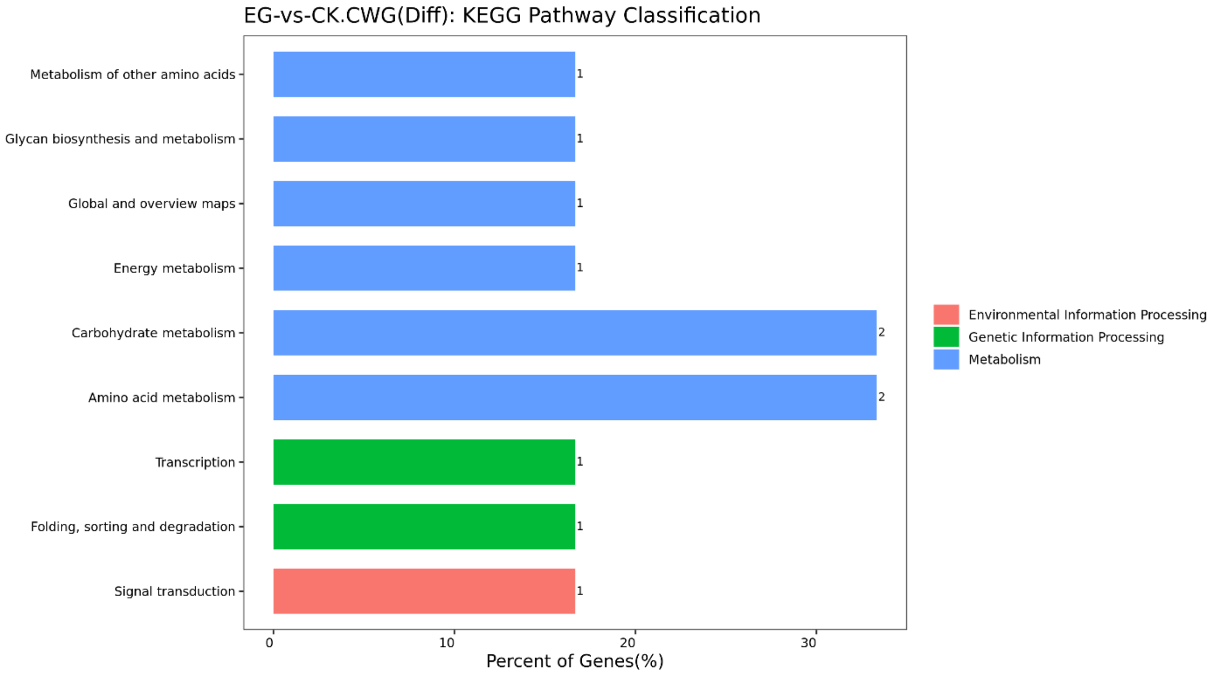
EG-vs-CK.CWG(Diff): KEGG Pathway Classification
Metabolism of other amino acids
Glycan biosynthesis and metabolism
Global and overview maps
Energy metabolism
Carbohydrate metabolism
Amino acid metabolism
Transcription
Folding, sorting and degradation
Signal transduction
1
1
1
1
2
2
1
1
1
0
10
20
30
Percent of Genes(%)
Environmental Information Processing
Genetic Information Processing
Metabolism

E

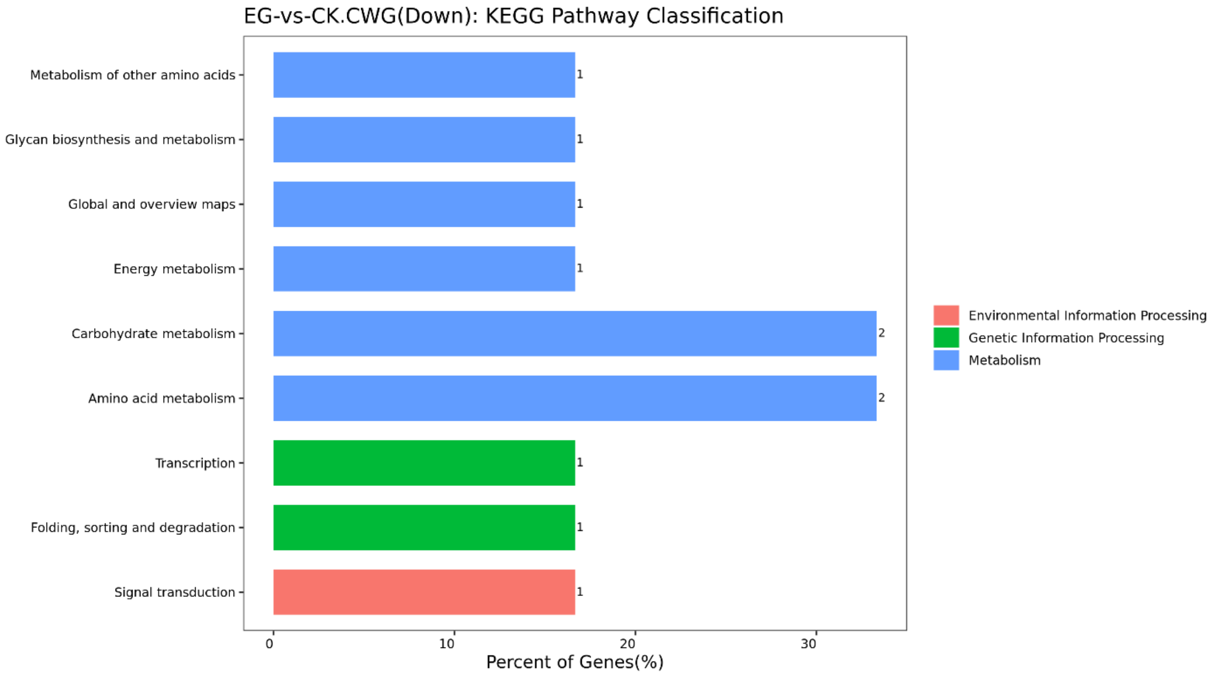

Figure S21. Classification of DMGs at the mCG and mCWG sites involved in the KEGG pathways. (**A**) Classification of DMGs in the mCG sites; (**B**,**C**) classification in the down and upregulated mCG sites.(**D**) Classification of DMGs in the mCWG sites (**E**) Classification in the downregulated mCWG sites

A

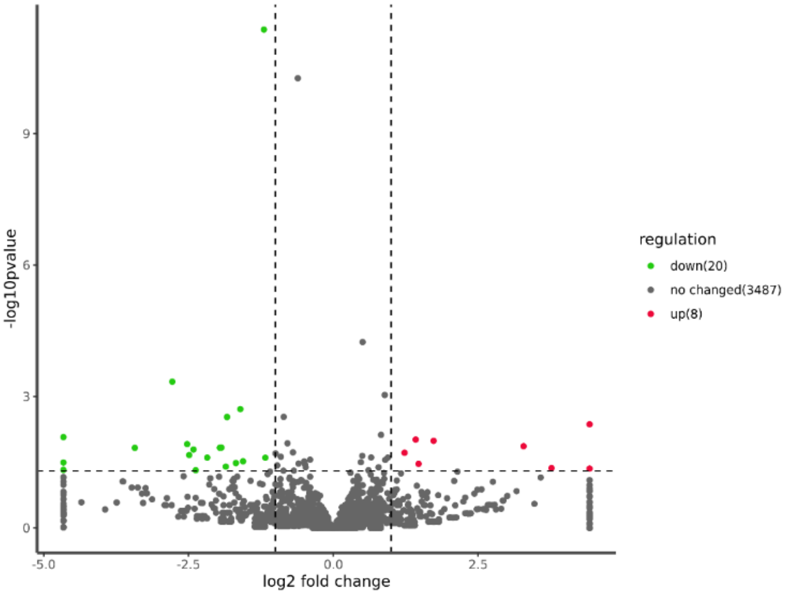

B

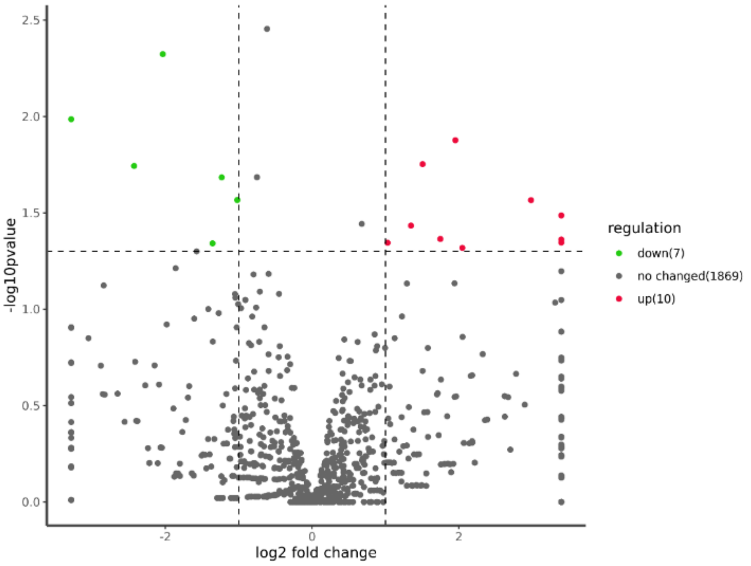

Figure S22. Differential methylation gene at the promoter level (DMGP) at CG and CWG sites between EG-vs-CK. (**A**) DMGP at the CG sites; (**B**) DMGP at the CWG sites. The red color is upregulation, and the green color is downregulation

A

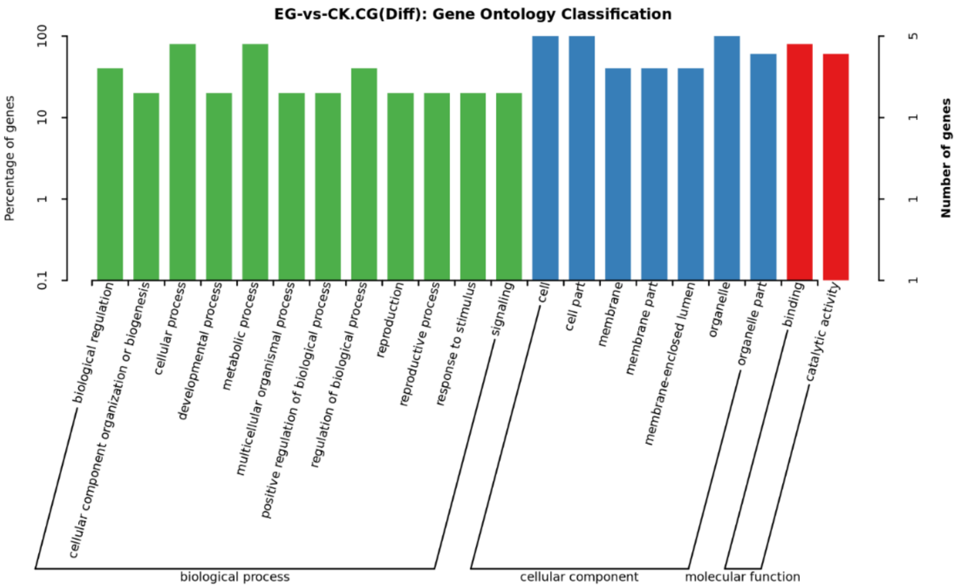

B

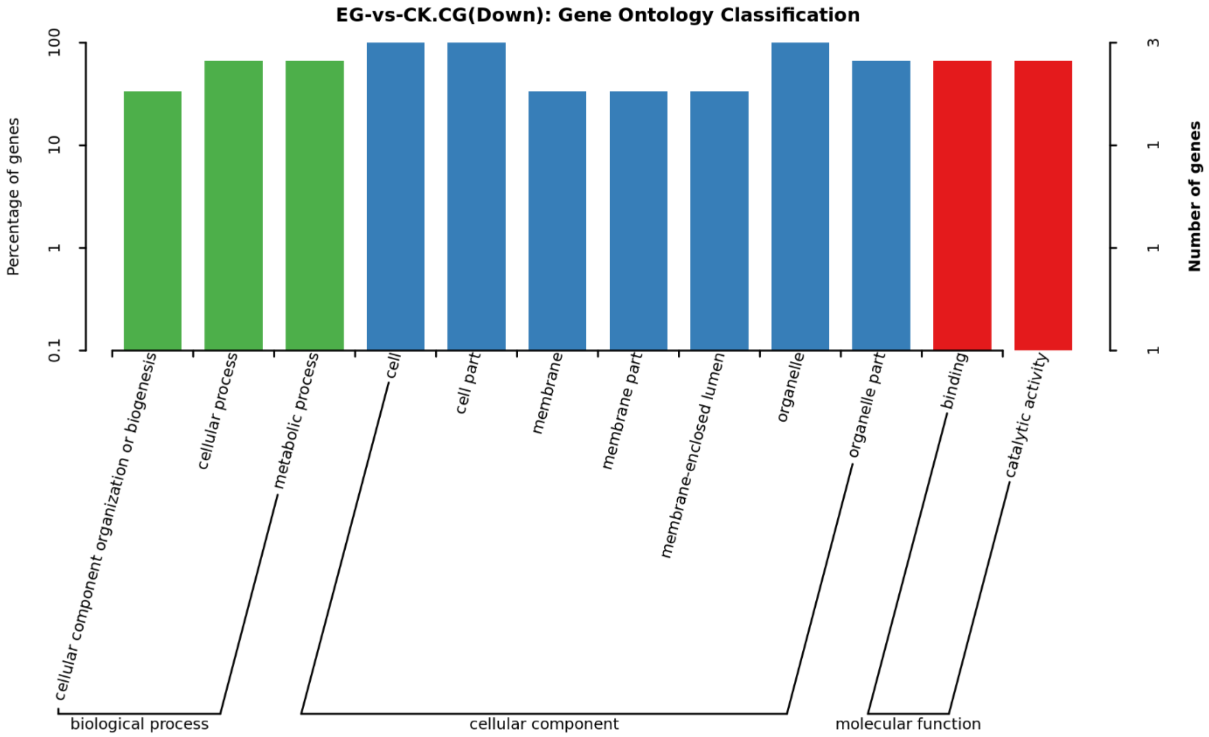
EG-vs-CK.CG(Down): Gene Ontology Classification
Percentage of genes
100
10
1
0.1
Number of genes
3
1
1
1
cellular component organization or biogenesis
cellular process
metabolic process
cell
cell part
membrane
membrane part
membrane-enclosed lumen
organelle
organelle part
binding
catalytic activity
biological process
cellular component
molecular function

C

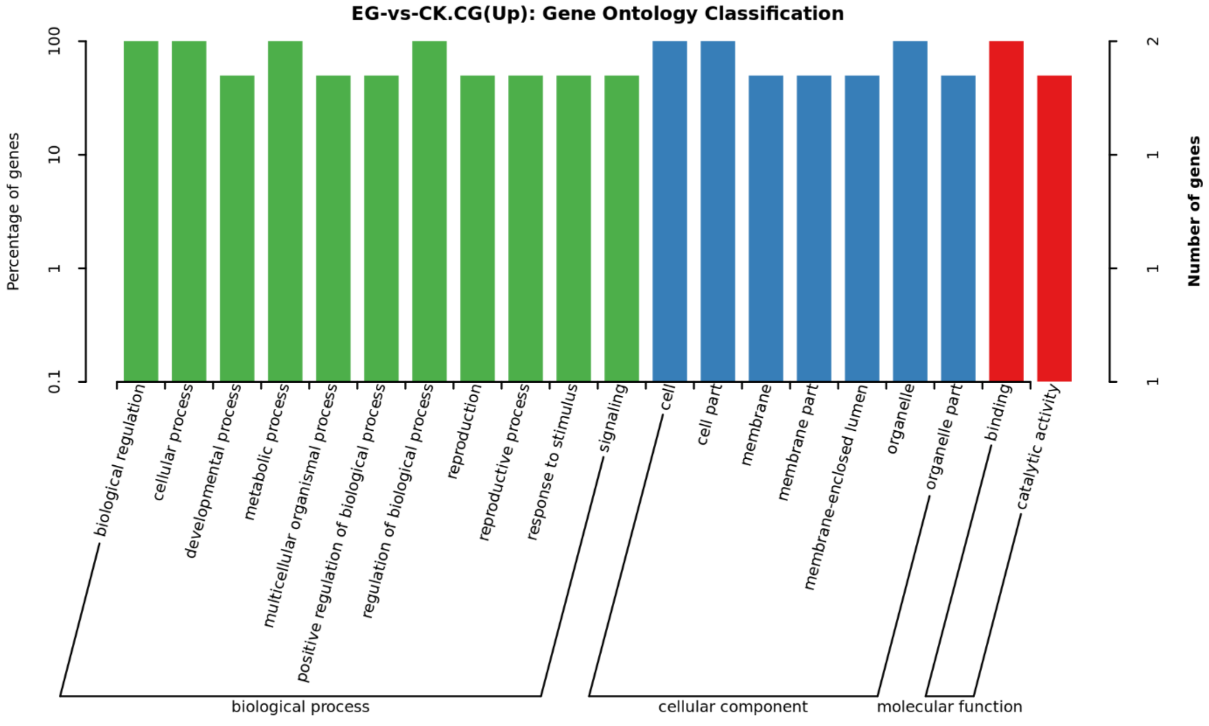
EG-vs-CK.CG(Up): Gene Ontology Classification
Percentage of genes
100
10
1
0.1
Number of genes
2
1
1
1
biological regulation
cellular process
developmental process
metabolic process
multicellular organismal process
positive regulation of biological process
regulation of biological process
reproduction
reproductive process
response to stimulus
signaling
cell
cell part
membrane
membrane part
membrane-enclosed lumen
organelle
organelle part
binding
catalytic activity
biological process
cellular component
molecular function

D

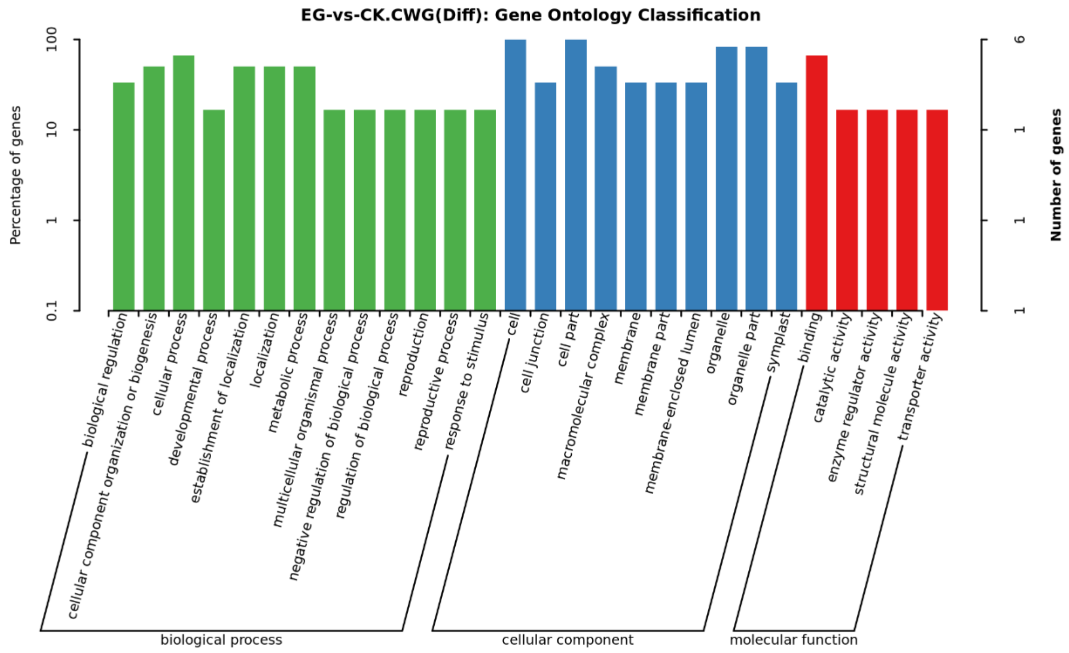
EG-vs-CK.CWG(Diff): Gene Ontology Classification
Percentage of genes
Number of genes
100
10
1
0.1
6
1
1
1
biological regulation
cellular component organization or biogenesis
cellular process
developmental process
establishment of localization
localization
metabolic process
multicellular organismal process
negative regulation of biological process
regulation of biological process
reproduction
reproductive process
response to stimulus
cell
cell junction
cell part
macromolecular complex
membrane
membrane part
membrane-enclosed lumen
organelle
organelle part
symplast
binding
catalytic activity
enzyme regulator activity
structural molecule activity
transporter activity
biological process
cellular component
molecular function

E

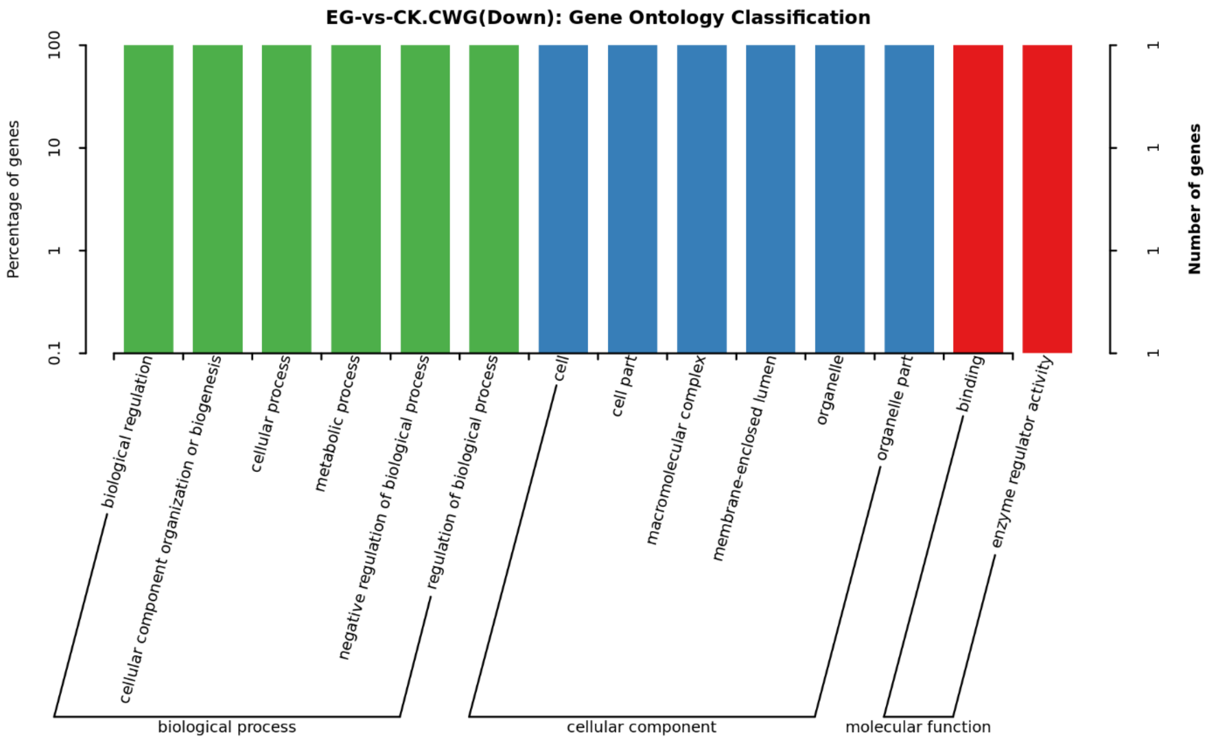
EG-vs-CK.CWG(Down): Gene Ontology Classification
Percentage of genes
Number of genes
100
10
1
0.1
1
1
1
1
biological regulation
cellular component organization or biogenesis
cellular process
metabolic process
negative regulation of biological process
regulation of biological process
cell
cell part
macromolecular complex
membrane-enclosed lumen
organelle
organelle part
binding
enzyme regulator activity
biological process
cellular component
molecular function

F

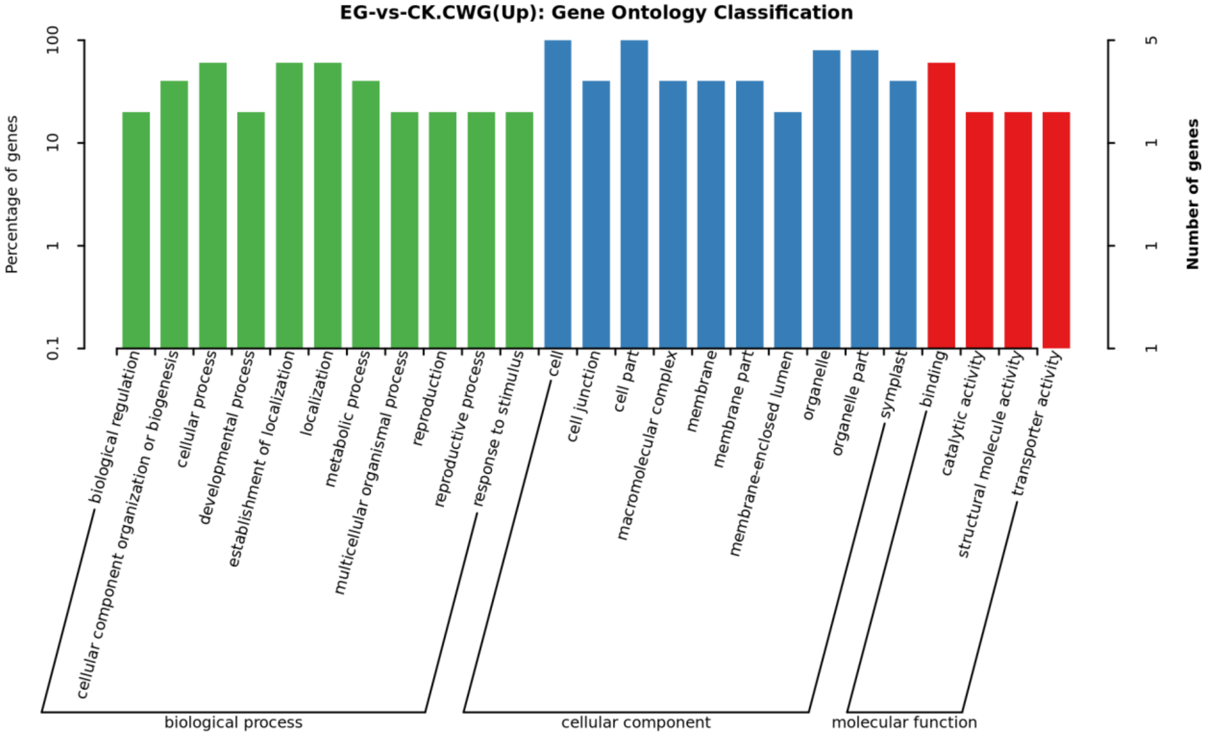

Figure S23. Comparison of the GO classification of the DMPGs at the mCG and mCWG sites. (**A**) GO classification of the DMPGs at the mCG sites; (**B**,**C**) The DMPGs in the GO terms at the mCG sites that were down and upregulated; (**D**) GO classification of the DMPGs at the mCWG sites; (**E**, **F**) The DMPGs in the GO terms at the mCWG sites that were down and upregulated

A

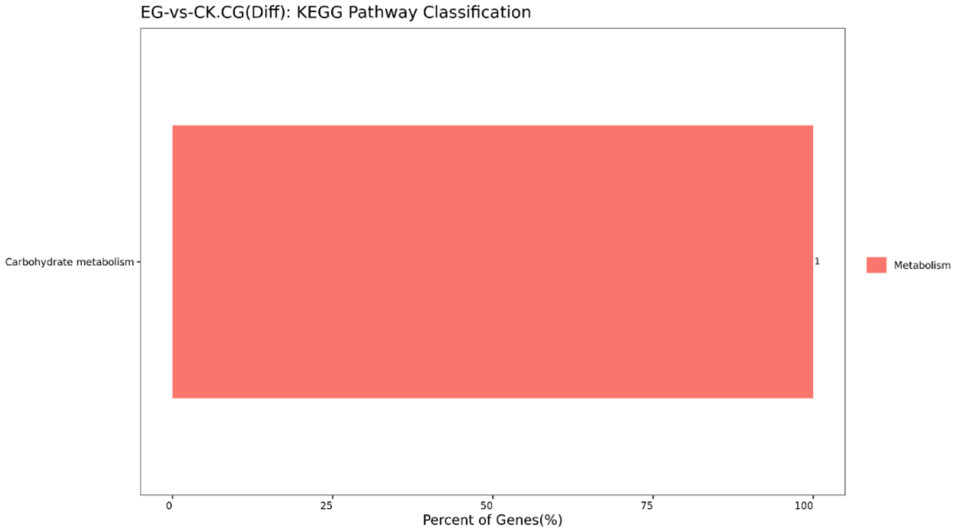

B

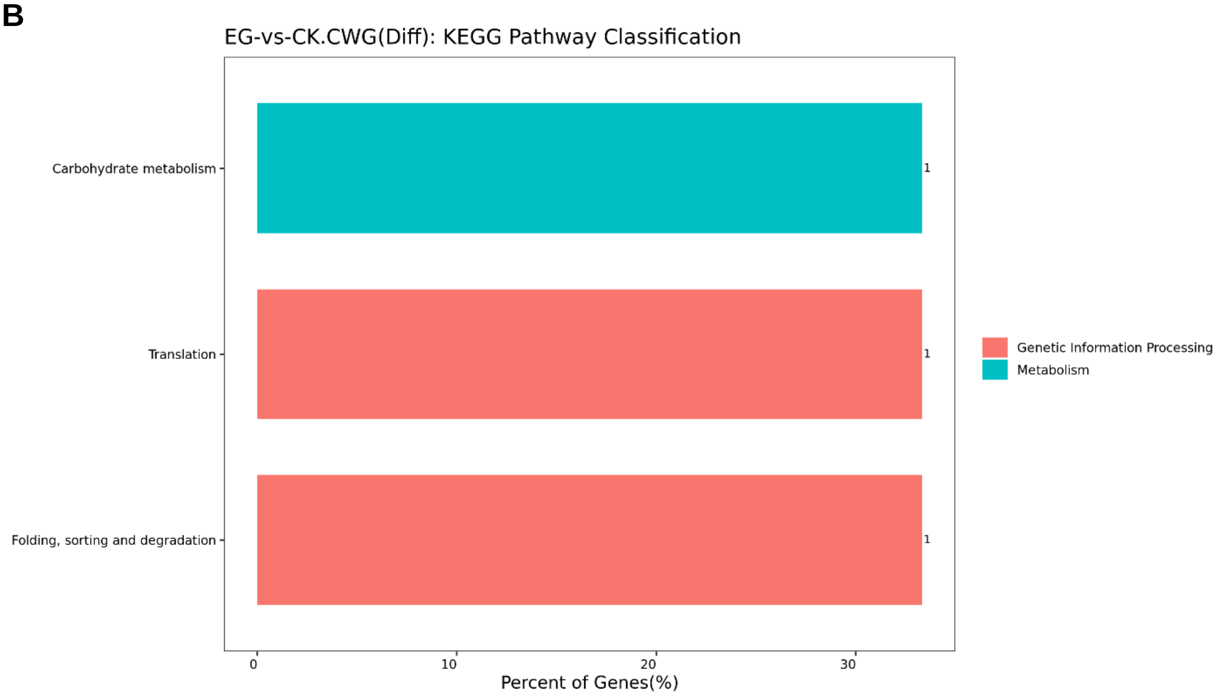

Figure S24. KEGG functional analysis of the DMPGs. (**A**) classification at the mCG sites; (**B**) classification at the mCWG sites
